# Normative Modeling of Brain Morphometry in Clinical High Risk for Psychosis

**DOI:** 10.1001/jamapsychiatry.2023.3850

**Published:** 2023-10-11

**Authors:** ENIGMA Clinical High Risk for Psychosis Working Group, Shalaila S. Haas, Ruiyang Ge, Ingrid Agartz, G. Paul Amminger, Ole A. Andreassen, Peter Bachman, Inmaculada Baeza, Sunah Choi, Tiziano Colibazzi, Vanessa L. Cropley, Camilo de la Fuente-Sandoval, Bjørn H. Ebdrup, Adriana Fortea, Paolo Fusar-Poli, Birte Yding Glenthøj, Louise Birkedal Glenthøj, Kristen M. Haut, Rebecca A. Hayes, Karsten Heekeren, Christine I. Hooker, Wu Jeong Hwang, Neda Jahanshad, Michael Kaess, Kiyoto Kasai, Naoyuki Katagiri, Minah Kim, Jochen Kindler, Shinsuke Koike, Tina D. Kristensen, Jun Soo Kwon, Stephen M. Lawrie, Irina Lebedeva, Jimmy Lee, Imke L. J. Lemmers-Jansen, Ashleigh Lin, Xiaoqian Ma, Daniel H. Mathalon, Philip McGuire, Chantal Michel, Romina Mizrahi, Masafumi Mizuno, Paul Møller, Ricardo Mora-Durán, Barnaby Nelson, Takahiro Nemoto, Merete Nordentoft, Dorte Nordholm, Maria A. Omelchenko, Christos Pantelis, Jose C. Pariente, Jayachandra M. Raghava, Francisco Reyes-Madrigal, Jan I. Røssberg, Wulf Rössler, Dean F. Salisbury, Daiki Sasabayashi, Ulrich Schall, Lukasz Smigielski, Gisela Sugranyes, Michio Suzuki, Tsutomu Takahashi, Christian K. Tamnes, Anastasia Theodoridou, Sophia I. Thomopoulos, Paul M. Thompson, Alexander S. Tomyshev, Peter J. Uhlhaas, Tor G. Værnes, Therese A. M. J. van Amelsvoort, Theo G. M. van Erp, James A. Waltz, Christina Wenneberg, Lars T. Westlye, Stephen J. Wood, Juan H. Zhou, Dennis Hernaus, Maria Jalbrzikowski, René S. Kahn, Cheryl M. Corcoran, Sophia Frangou

**Affiliations:** 1 Department of Psychiatry, Icahn School of Medicine at Mount Sinai, New York, New York; 2 Djavad Mowafaghian Centre for Brain Health, Department of Psychiatry, University of British Columbia, Vancouver, British Columbia, Canada; 3 Department of Psychiatry, University of British Columbia, Vancouver, British Columbia, Canada; 4 Department of Psychiatric Research, Diakonhjemmet Hospital, Oslo, Norway; 5 Centre for Psychiatry Research, Department of Clinical Neuroscience, Karolinska Institutet and Stockholm Health Care Services, Stockholm County Council, Stockholm, Sweden; 6 KG Jebsen Center for Neurodevelopmental Disorders, University of Oslo, Oslo, Norway; 7 Norwegian Centre for Mental Disorders Research, Institute of Clinical Medicine, University of Oslo, Oslo, Norway; 8 Centre for Youth Mental Health, University of Melbourne, Melbourne, Victoria, Australia; 9 Orygen, Melbourne, Victoria, Australia; 10 NORMENT, Division of Mental Health and Addiction, Oslo University Hospital and Institute of Clinical Medicine, University of Oslo, Oslo, Norway; 11 Department of Psychiatry and Behavioral Sciences, Boston Children’s Hospital, Boston, Massachusetts; 12 Centro de Investigación Biomédica en Red de Salud Mental (CIBERSAM)-ISCIII, Madrid Spain; 13 Institut d’Investigacions Biomèdiques August Pi i Sunyer (FCRB-IDIBAPS), Barcelona, Spain; 14 Child and Adolescent Psychiatry and Psychology Department, 2021SGR01319, Institute of Neuroscience, Hospital Clínic de Barcelona, Barcelona, Spain; 15 Department of Medicine, University of Barcelona, Barcelona, Spain; 16 Department of Brain and Cognitive Sciences, Seoul National University College of Natural Sciences, Seoul, Republic of Korea; 17 Department of Psychiatry, Columbia University, New York, New York; 18 New York State Psychiatric Institute, New York; 19 Melbourne Neuropsychiatry Centre, Department of Psychiatry, University of Melbourne and Western Health, Carlton South, VIC, Australia; 20 Laboratory of Experimental Psychiatry, Instituto Nacional de Neurología y Neurocirugía, Mexico City, Mexico; 21 Centre for Neuropsychiatric Schizophrenia Research and Centre for Clinical Intervention and Neuropsychiatric Schizophrenia Research, Mental Health Centre Glostrup, University of Copenhagen, Glostrup, Denmark; 22 Department of Clinical Medicine, Faculty of Health and Medical Sciences, University of Copenhagen, Copenhagen, Denmark; 23 Department of Psychiatry and Psychology, Hospital Clínic de Barcelona, Barcelona, Spain; 24 Fundació Clínic Recerca Biomèdica, Universitat de Barcelona, Barcelona, Spain; 25 Department of Psychosis Studies, Early Psychosis: Interventions and Clinical-Detection Lab, Institute of Psychiatry, Psychology and Neuroscience, King’s College London, London, United Kingdom; 26 Department of Brain and Behavioral Sciences, University of Pavia, Pavia, Italy; 27 Copenhagen Research Center for Mental Health, Mental Health Center Copenhagen, University of Copenhagen, Copenhagen, Denmark; 28 Department of Psychiatry and Behavioral Sciences, Rush University Medical Center, Chicago, Illinois; 29 Department of Psychiatry and Psychotherapy, LVR-Hospital Cologne, Cologne, Germany; 30 Department of Psychiatry, Psychotherapy and Psychosomatics, Psychiatric University Hospital Zurich, University of Zurich, Zurich, Switzerland; 31 Catholic Kwandong University College of Medicine, Gangneung, Republic of Korea; 32 Imaging Genetics Center, Mark and Mary Stevens Neuroimaging and Informatics Institute, Keck School of Medicine, University of Southern California, Marina del Rey; 33 Department of Child and Adolescent Psychiatry, University of Heidelberg, Heidelberg, Germany; 34 University Hospital of Child and Adolescent Psychiatry and Psychotherapy, University of Bern, Bern, Switzerland; 35 Department of Neuropsychiatry, Graduate School of Medicine, The University of Tokyo, Tokyo, Japan; 36 The University of Tokyo Institute for Diversity and Adaptation of Human Mind, The University of Tokyo, Tokyo, Japan; 37 The International Research Center for Neurointelligence at The University of Tokyo Institutes for Advanced Study, The University of Tokyo, Tokyo, Japan; 38 Department of Neuropsychiatry, Toho University School of Medicine, Tokyo, Japan; 39 Department of Neuropsychiatry, Seoul National University Hospital, Seoul, Republic of Korea; 40 Department of Psychiatry, Seoul National University College of Medicine, Seoul, Republic of Korea; 41 Center for Evolutionary Cognitive Sciences, Graduate School of Art and Sciences, The University of Tokyo, Tokyo, Japan; 42 Centre for Neuropsychiatric Schizophrenia Research, Mental Health Centre Glostrup, Copenhagen University Hospital, Glostrup, Denmark; 43 Division of Psychiatry, University of Edinburgh, Edinburgh, United Kingdom; 44 Laboratory of Neuroimaging and Multimodal Analysis, Mental Health Research Center, Moscow, Russian Federation; 45 Department of Psychosis, Institute of Mental Health, Singapore; 46 Lee Kong Chian School of Medicine, Nanyang Technological University, Singapore; 47 Faculty of Behavioural and Movement Sciences, Department of Clinical, Neuro and Developmental Psychology, Vrije Universiteit Amsterdam, Amsterdam, the Netherlands; 48 Department of Psychosis Studies, Institute of Psychiatry, Psychology and Neuroscience, King’s College London, London, United Kingdom; 49 Telethon Kids Institute, The University of Western Australia, Perth, Western Australia, Australia; 50 National Clinical Research Center for Mental Disorders and Department of Psychiatry, The Second Xiangya Hospital of Central South University, Changsha, China; 51 Department of Psychiatry and Behavioral Sciences, University of California, San Francisco, San Francisco; 52 San Francisco Veterans Affairs Health Care System, San Francisco, California; 53 Department of Psychiatry, University of Oxford, Oxford, United Kingdom; 54 Douglas Research Center, McGill Univesity, Montreal, Quebec, Canada; 55 Department of Psychiatry, McGill University, Montreal, Quebec, Canada; 56 Tokyo Metropolitan Matsuzawa Hospital, Tokyo, Japan; 57 Department for Mental Health Research and Development, Division of Mental Health and Addiction, Vestre Viken Hospital Trust, Drammen, Norway; 58 Emergency Department, Hospital Fray Bernardino Álvarez, Mexico City, Mexico; 59 Department of Youth Psychiatry, Mental Health Research Center, Moscow, Russian Federation; 60 Florey Institute of Neuroscience and Mental Health, Center for Mental Health, Parkville, Victoria, Australia; 61 Magnetic Resonance Imaging Core Facility, Institut d’Investigacions Biomèdiques August Pi i Sunyer, Barcelona, Spain; 62 Functional Imaging Unit, Department of Clinical Physiology, Nuclear Medicine and PET, University of Copenhagen, Glostrup, Denmark; 63 Oslo University Hospital and University of Oslo, Institute of Clinical Medicine, University of Oslo, Oslo, Norway; 64 Department of Psychiatry and Psychotherapy, Charité Universitätsmedizin, Berlin, Germany; 65 Department of Psychiatry, Psychotherapy and Psychosomatics, University of Zurich, Zurich, Switzerland; 66 Department of Psychiatry, University of Pittsburgh, Pittsburgh, Pennsylvania; 67 Department of Neuropsychiatry, University of Toyama Graduate School of Medicine and Pharmaceutical Sciences, Toyama, Japan; 68 Research Center for Idling Brain Science, University of Toyama, Toyama, Japan; 69 Priority Centre for Brain and Mental Health Research, The University of Newcastle, Newcastle, New South Wales, Australia; 70 Priority Research Centre Grow Up Well, The University of Newcastle, Newcastle, New South Wales, Australia; 71 Department of Child and Adolescent Psychiatry, Psychiatric University Hospital Zurich, University of Zurich, Zurich, Switzerland; 72 PROMENTA Research Center, Department of Psychology, University of Oslo, Oslo, Norway; 73 Department of Child and Adolescent Psychiatry, Charité Universitätsmedizin, Berlin, Germany; 74 Institute of Neuroscience and Psychology, University of Glasgow, Glasgow, United Kingdom; 75 Early Intervention in Psychosis Advisory Unit for South-East Norway, TIPS Sør-Øst, Division of Mental Health and Addiction, Oslo University Hospital, Oslo, Norway; 76 Department of Psychiatry and Neuropsychology, School for Mental Health and Neuroscience, Faculty of Health Medicine and Life Sciences, Maastricht University, Maastricht, the Netherlands; 77 Clinical Translational Neuroscience Laboratory, Department of Psychiatry and Human Behavior, University of California, Irvine, Irvine; 78 Center for the Neurobiology of Learning and Memory, University of California, Irvine, Irvine; 79 Maryland Psychiatric Research Center, University of Maryland School of Medicine, Baltimore; 80 Department of Psychology, University of Oslo, Oslo, Norway; 81 School of Psychology, University of Birmingham, Birmingham, United Kingdom; 82 Center for Sleep and Cognition, Yong Loo Lin School of Medicine, National University of Singapore, Singapore; 83 Center for Translational Magnetic Resonance Research, Yong Loo Lin School of Medicine, National University of Singapore, Singapore; 84 Department of Psychiatry, Harvard Medical School, Boston, Massachusetts; 85 Mental Illness Research, Education and Clinical Center, James J. Peters VA Medical Center, New York, New York

## Abstract

**Importance:**

The lack of robust neuroanatomical markers of psychosis risk has been traditionally attributed to heterogeneity. A complementary hypothesis is that variation in neuroanatomical measures in individuals at psychosis risk may be nested within the range observed in healthy individuals.

**Objective:**

To quantify deviations from the normative range of neuroanatomical variation in individuals at clinical high risk for psychosis (CHR-P) and evaluate their overlap with healthy variation and their association with positive symptoms, cognition, and conversion to a psychotic disorder.

**Design, Setting, and Participants:**

This case-control study used clinical-, IQ-, and neuroimaging software (FreeSurfer)–derived regional measures of cortical thickness (CT), cortical surface area (SA), and subcortical volume (SV) from 1340 individuals with CHR-P and 1237 healthy individuals pooled from 29 international sites participating in the Enhancing Neuroimaging Genetics Through Meta-analysis (ENIGMA) Clinical High Risk for Psychosis Working Group. Healthy individuals and individuals with CHR-P were matched on age and sex within each recruitment site. Data were analyzed between September 1, 2021, and November 30, 2022.

**Main Outcomes and Measures:**

For each regional morphometric measure, deviation scores were computed as *z* scores indexing the degree of deviation from their normative means from a healthy reference population. Average deviation scores (ADS) were also calculated for regional CT, SA, and SV measures and globally across all measures. Regression analyses quantified the association of deviation scores with clinical severity and cognition, and 2-proportion *z* tests identified case-control differences in the proportion of individuals with infranormal (*z* < −1.96) or supranormal (*z* > 1.96) scores.

**Results:**

Among 1340 individuals with CHR-P, 709 (52.91%) were male, and the mean (SD) age was 20.75 (4.74) years. Among 1237 healthy individuals, 684 (55.30%) were male, and the mean (SD) age was 22.32 (4.95) years. Individuals with CHR-P and healthy individuals overlapped in the distributions of the observed values, regional z scores, and all ADS values. For any given region, the proportion of individuals with CHR-P who had infranormal or supranormal values was low (up to 153 individuals [<11.42%]) and similar to that of healthy individuals (<115 individuals [<9.30%]). Individuals with CHR-P who converted to a psychotic disorder had a higher percentage of infranormal values in temporal regions compared with those who did not convert (7.01% vs 1.38%) and healthy individuals (5.10% vs 0.89%). In the CHR-P group, only the ADS SA was associated with positive symptoms (β = −0.08; 95% CI, −0.13 to −0.02; *P* = .02 for false discovery rate) and IQ (β = 0.09; 95% CI, 0.02-0.15; *P* = .02 for false discovery rate).

**Conclusions and Relevance:**

In this case-control study, findings suggest that macroscale neuromorphometric measures may not provide an adequate explanation of psychosis risk.

## Key Points

**Question:**

Are brain morphometric changes that deviate significantly from healthy variation associated with the risk of psychosis?

**Findings:**

In this case-control study of 1340 individuals at clinical high risk for psychosis and 1237 healthy participants, individual-level variation in macroscale neuromorphometric measures in the group at clinical high risk for psychosis was largely nested within healthy variation and was not associated with the severity of positive symptoms or conversion to a psychotic disorder.

**Meaning:**

The findings suggest the macroscale neuromorphometric measures have limited utility as diagnostic biomarkers of psychosis risk.

## Teaser

This case-control study assesses the association of brain morphometric changes that deviate from the normative range and with positive symptoms, cognition, and conversion to a psychotic disorder among individuals at high risk for psychosis.

## Introduction

Schizophrenia is a mental disorder characterized by psychotic and cognitive symptoms^1^ and substantial psychosocial disability.^2^ Similar abnormalities are also present in individuals at clinical high risk for psychosis (CHR-P) who experience attenuated or brief psychotic symptoms,^3^ cognitive difficulties, and elevated risk of developing psychosis at rates of 20% at 2 years and 35% at 10 years.^4^ A better understanding of the neurobiological features of CHR states holds the promise of improving early detection and preventive strategies.^5^

Multiple magnetic resonance imaging (MRI) studies have reported neuroanatomical alterations in individuals with CHR-P compared with healthy individuals. Two meta-analyses^6,7^ and a mega-analysis^8^ of brain morphometric data from 1792 individuals with CHR-P and 1377 healthy individuals from the CHR-P Working Group of the Enhancing Neuroimaging Genetics Through Meta-analysis (ENIGMA) Consortium have highlighted cortical thickness (CT) reductions of small effect size (range, −0.18 to −0.09) in individuals with CHR-P.

Psychiatric neuroimaging has turned to normative modeling, which quantifies individual-level deviation in brain-derived phenotypes relative to a normative reference population.^9^ The advantage of this approach is that it can test whether psychiatric disorders are associated with substantial deviation from healthy variation in measures of brain organization. Normative modeling has yet to be applied to CHR-P states, but there are 2 studies^10,11^ on patients with established schizophrenia that are of direct relevance. In both studies,^10,11^ brain morphometric measures with values below the 5th percentile or above the 95th percentile of the normative range were respectively considered infranormal and supranormal. Lv et al^10^ calculated normative models of CT from 195 healthy individuals and applied them to 322 individuals with schizophrenia; 10% to 15% of patients had infranormal CT values in temporal and ventromedial frontal regions, and 3% of patients had supranormal values mainly in the paracentral lobule. Wolfers and colleagues^11^ developed normative models from voxel-based morphometric data from 3 samples of healthy individuals (sample 1: n = 400; sample 2: n = 312; sample 3: n = 256) and applied them to data from corresponding samples of patients with schizophrenia (sample 1: n = 94; sample 2: n = 105; sample 3: n = 163). Only a low percentage of voxels (<2%) had extreme values in patients across samples; voxels with infranormal values were mostly located within temporal, medial frontal, and posterior cingulate regions.^11^

It is currently unknown whether regional deviations from healthy variation in brain morphometry are present in individuals with CHR-P and whether they are associated with clinical status, positive symptoms, or cognition. One study^12^ has suggested that normative deviation scores are better than raw regional brain volumes in estimating psychotic symptoms. Addressing these questions is important for 2 reasons. First, vulnerabilities during brain development, as inferred from the presence of deviations from normative neuroanatomical trajectories, may set the scene for the brain changes observed in established cases of schizophrenia. Second, deviation from healthy variation in neuroanatomy may aid the identification of those individuals with CHR-P who convert to or experience more severe clinical presentations. To test these hypotheses, the current study applied normative modeling to regional neuromorphometric measures derived from the ENIGMA CHR-P Working Group sample, which represents the largest available data set of individual-level morphometric measures from individuals with CHR-P.^8^

## Methods

### Study Sample

This case-control study was approved by Icahn School of Medicine at Mount Sinai. Ethical approval and written informed consent for data collection and sharing were obtained from the institutional review board and study participants at each site. Participant data were shared after all identifying information was removed.

The study sample was derived from the pooled data set of individuals with CHR-P and healthy individuals held by the ENIGMA CHR-P Working Group (eMethods and eTable 1 in Supplement 1). Healthy individuals and individuals with CHR-P were matched on age and sex within each recruitment site (eTable 1 in Supplement 1). At each site, CHR-P status was ascertained using either the Structured Interview for Psychosis-Risk Syndromes (SIPS)^13,14^ or the Comprehensive Assessment of At-Risk Mental States (CAARMS)^15^ (eMethods and eTable 2 in Supplement 1). Additional site-specific eligibility criteria are shown in eTable 1 in Supplement 1. Data were analyzed between September 1, 2021, and November 30, 2022.

At each site, whole brain T1-weighted MRI data obtained from each participant (eMethods and eTable 3 in Supplement 1) were parcellated and segmented using standard neuroimaging software (FreeSurfer; Laboratory for Computational Neuroimaging, Athinoula A. Martinos Center for Biomedical Imaging)^16^ to yield estimates of total intracranial volume, regional measures of CT (n = 68), surface area (SA) (n = 68), and subcortical volume (SV) (n = 14). These measures were then assessed using the ENIGMA Consortium quality assessment pipeline.^17,18,19,20^

The current study sample comprised participants who had both high-quality brain morphometric data and complete SIPS or CAARMS ratings at the time of their scan (eMethods and eFigure 1 in Supplement 1 for study sample selection flowchart). Based on these criteria, we included 1340 individuals with CHR-P and 1237 healthy individuals (Table; eTables 1-5 and eFigure 2 in Supplement 1). Conversion status at a mean (SD) follow-up time of 19.71 (13.97) months was available for 1097 individuals with CHR-P (Table; eTable 4 in Supplement 1). Individuals with CHR-P who converted to a psychotic disorder (CHR-PC) (n = 157) had significantly higher positive symptoms at the time of scanning (mean [SD] *z* score, 0.21 [1.08]) than those who did not convert to a psychotic disorder (CHR-PNC) (n = 940; mean [SD] *z* score, −0.05 [1.01]; *t* = 2.99; *P* = .003), but the 2 groups did not differ in age, sex, or IQ.

**Table.** Characteristics of the Sample at Clinical High Risk for Psychosis

Characteristic	Participants
**All individuals with CHR-P (n = 1340)**	
Age, mean (SD), y	20.75 (4.74)
Sex, No. (%)	
Female	631 (47.09)
Male	709 (52.91)
SIPS positive symptoms score, mean (SD)^a^^,^^b^	10.93 (4.66)
CAARMS positive symptoms score, mean (SD)^a^^,^^c^	10.37 (4.03)
IQ, mean (SD) *z* score^d^	−0.21 (1.00)
Prescribed antipsychotic medication, No. (%)^e^	243 (18.63)
Follow-up, mean (SD), mo^f^	19.71 (13.97)
Individuals who converted to a psychotic disorder, No. (%)^f^	157 (14.31)
**Individuals with CHR-P who converted to a psychotic disorder (n = 157)**	
Age, mean (SD), y	20.09 (4.68)
Sex, No. (%)	
Female	64 (40.76)
Male	93 (59.24)
SIPS positive symptoms score, mean (SD)^g^	12.12 (5.06)
CAARMS positive symptoms score, mean (SD)^h^	10.71 (4.24)
IQ, mean (SD) *z* score^i^	−0.29 (1.03)
Prescribed antipsychotic medication, No. (%)^j^	32 (20.38)

Abbreviations: CAARMS, Comprehensive Assessment of At-Risk Mental States; CHR-P, clinical high risk for psychosis; SIPS, Structured Interview for Psychosis-Risk Syndromes.

^a^ Positive symptom ratings at the time of scanning were available for the entire sample of individuals with CHR-P (n = 1340), assessed either with the SIPS or the CAARMS.

^b^ The SIPS was used to assess positive symptoms in 806 participants with CHR-P.

^c^ The CAARMS was used to assess positive symptoms in 534 participants with CHR-P.

^d^ Estimates of IQ were available for 924 participants with CHR-P; *z* scores were used to accommodate site differences in the instruments used (eTable 1 in Supplement 1).

^e^ Medication status at the time of scanning was available for 1304 individuals with CHR-P.

^f^ Conversion status was known for 1097 participants with CHR-P, but information about the length of the follow-up period was available for only 975 individuals with CHR-P.

^g^ The SIPS was used to assess 115 individuals with CHR-P who converted to a psychotic disorder.

^h^ The CAARMS was used to assess 42 individuals with CHR-P who converted to a psychotic disorder.

^i^ Estimates of IQ were available for 109 individuals with CHR-P who converted to a psychotic disorder.

^j^ Medication status was available for 157 individuals with CHR-P who converted to a psychotic disorder.

### Clinical Data

The ratings of CAARMS and SIPS converged only for positive symptoms (eMethods and eTable 2 in Supplement 1); these ratings were converted to *z* scores to enable cross-site harmonization. Similarly, IQ estimates were converted to *z* scores to accommodate the different instruments used across sites (eTable 1 in Supplement 1). Information was also available on medication exposure at the time of scanning.

### Normative Modeling of Brain Morphometry

Sex-specific normative models for each neuroimaging software (FreeSurfer)–derived regional CT, SA, and SV measure (eTable 6 in Supplement 1) were generated using a normative modeling framework for neuroimaging measures that belongs to the category of standard life charts (CentileBrain)^21^ and was developed by the ENIGMA Lifespan Group. The code and the models are publicly available as a web portal in the context of open science^22^; the normative models for each regional measure in the framework were developed using data from an independent multisite sample of 37 407 healthy individuals (53.3% female; aged 3-90 years) (eMethods in Supplement 1).^21^ In the normative modeling framework, the optimal models were defined following benchmarking of 8 different algorithms (ordinary least squares regression; bayesian linear regression; generalized additive models for location, scale, and shape; parametric λ, μ, and ς method; multivariable fractional polynomial regression; gaussian process regression; warped bayesian linear regression; and hierarchical bayesian regression) and covariate optimization by comparative evaluation of improvements in model accuracy with the addition of 10 covariates (alone or in combinations) pertaining to site, acquisition features, parcellation software version, and global neuroimaging measures.^21^ Through this pipeline, we identified multivariable fractional polynomial regression as the optimal algorithm; the optimal covariate combination involved site harmonization (ComBat-GAM [combatting batch effects–generalized additive model])^23^ and the inclusion of intracranial volume, mean CT, and mean SA in the models of the regional measures of SV, CT, and SA, respectively.

### Computing Deviation Scores of Regional Morphometric Measures

The model parameters in the normative framework (CentileBrain) were then applied to each regional CT, SA, and SV measure of the individuals with CHR-P and healthy individuals in the ENIGMA sample. For each measure in each participant, we estimated the degree of normative deviation from the reference population mean as a *z* score computed by subtracting the estimated value from the raw value of that measure, and then dividing the difference by the root mean square error of the model (eFigure 3 in Supplement 1).^24,25^ A positive or negative *z* score indicated that the value of the corresponding morphometric measure was higher or lower, respectively, than the normative mean. Per previous literature,^18,19^ we defined regional *z* scores as infranormal when below −1.96 or supranormal when above 1.96, corresponding to the 5th percentile and 95th percentile, respectively. Intermediate values (ie, *z* scores between −1.96 and 1.96) were designated as within normal range.

### Computation of Average Deviation Scores

We averaged the regional *z* scores in each participant to generate an average deviation score (ADS) for CT, SA, and SV. The ADS values were not weighted for the size of the region to enhance reproducibility. Positive or negative ADS values indicate a general pattern of deviations that are above or below the normative reference values. The ADS scores were further averaged to generate a global ADS. Using the same criteria as for the *z* scores, each ADS was also designated as infranormal, supranormal, or within the normal range. In supplemental analyses, we also explored multiple alternate definitions of ADS (eMethods in Supplement 1).

### Supplemental and Sensitivity Analyses

A number of additional analyses were undertaken to establish the robustness of the results. Specifically, we conducted traditional case-control comparisons of the observed neuromorphometric measures and repeated key analyses using observed data (in addition to *z* scores) to test for potential differential performance of these measures. We tested the robustness of the results to the spatial resolution of the input data and algorithm by repeating the normative analyses using the Schaeffer 400-parcels atlas or gray and white matter maps as input features and gaussian process regression or generalized additive models for location, scale, and shape as alternate algorithms (eMethods in Supplement 1). We addressed the potential heterogeneity of the CHR-P sample by repeating the analyses in subsets with specific subsyndromes (ie, attenuated psychotic symptoms syndrome, brief intermittent psychotic symptoms syndrome, and genetic risk and functional deterioration syndrome) (eMethods in Supplement 1). We tested associations with IQ and positive symptoms with observed neuromorphometric measures’ alternate ADS definitions using a leave-one-site-out approach to account for confounding effects of site and medication status.

### Statistical Analysis

Statistical significance across all tests performed was set at 2-sided *P* < .05 per the Benjamini-Hochberg false discovery rate (FDR) correction for multiple comparisons. Five main analyses were conducted. In the first analysis, we calculated the percentage of individuals with CHR-P and healthy individuals from the ENIGMA sample who had supranormal or infranormal *z* scores in any regional measure and in any ADS. Group differences in the proportion of individuals with supra- or infranormal *z* scores were examined using the 2-proportions *z* test implemented in R software, version 4.1.2 (R Foundation for Statistical Computing). In the second analysis, we used linear support vector classification with 10-fold cross-validation implemented in Python, version 3.8 (Python Software Foundation), to estimate diagnostic status (individuals with CHR-P vs healthy individuals and individuals with CHR-PC vs healthy individuals) using all of the regional *z* scores as input data. In the third analysis, within the CHR-P group, we used linear regression models (implemented with the *lm* function in R software, version 4.1.2) to assess associations between positive symptoms and IQ with each regional *z* score and each ADS. Age was included as a variable in all regression models due to its association with positive symptoms and IQ (*P* < .05 for FDR). Analyses were conducted with and without site as a random effect. In the fourth analysis, we used the brain basis set (BBS)^26^ method to identify multivariate patterns of associations of regional *z* scores with IQ and positive symptoms. The BBS is a multivariate estimation modeling method that decomposes neuroimaging measures into specific components before modeling (eMethods in Supplement 1). In the fifth analysis, we repeated the first 4 analyses separately for individuals with CHR-PC and CHR-PNC and for healthy individuals.

## Results

Among 1340 individuals with CHR-P, 709 (52.91%) were male and 631 (47.09%) were female; the mean (SD) age was 20.75 (4.74) years. Among 1237 healthy individuals, 684 (55.30%) were male and 553 (44.70%) were female; the mean (SD) age was 22.32 (4.95) years.

### Infra- and Supranormal Deviations in Brain Morphometry in Individuals With CHR-P and Healthy Individuals

**Figure 1.**
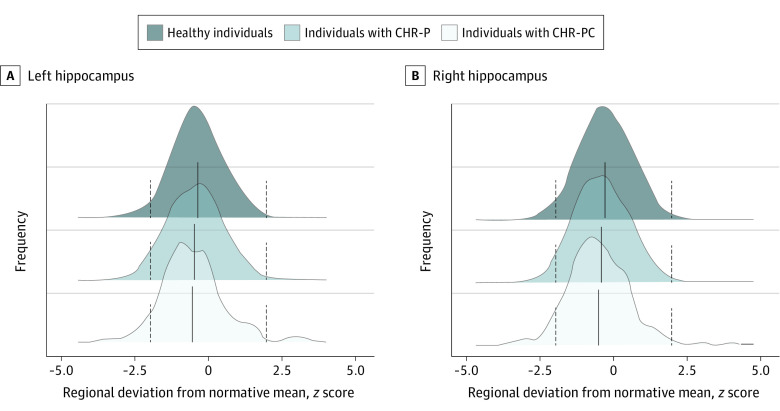
Distribution of Hippocampal Subcortical Volume Normative *z* Scores in the Study Sample The hippocampus is used as an exemplar because the same pattern was observed for all regions (eFigures 4 and 5 in Supplement 1). The dotted vertical lines represent the cutoffs for infranormal (*z* < −1.96) and supranormal (*z* > 1.96) values. CHR-P indicates clinical high risk for psychosis; and CHR-PC, clinical high risk for psychosis converted to a psychotic disorder.

**Figure 2.**
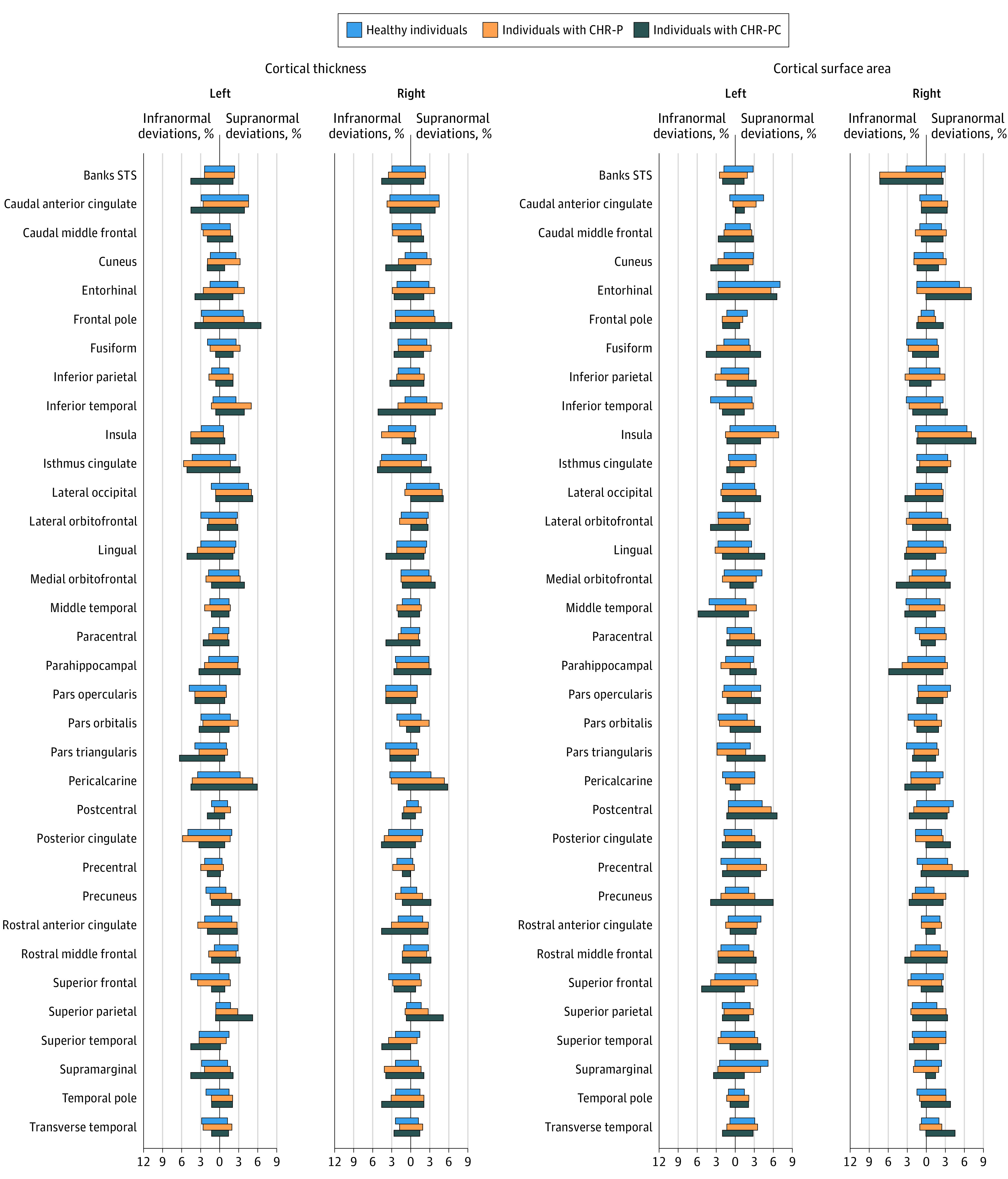
Percentage of Participants With Infra- or Supranormal Regional Normative *z* Scores Banks STS indicates banks of the superior temporal sulcus; CHR-P, clinical high risk for psychosis; and CHR-PC, clinical high risk for psychosis converted to a psychotic disorder.

The distributions of the *z* scores and observed values of all regional neuromorphometric measures of individuals with CHR-P and healthy individuals had complete overlap, both within each site and in the entire study sample, independent of the normative modeling method used (Figure 1; eFigures 4-7 and eResults in Supplement 1). The percentages of individuals with CHR-P and healthy individuals who had supra- or infranormal *z* scores in each morphometric measure are shown in Figure 2 and eFigure 8 in Supplement 1. Infranormal regional CT *z* scores were noted in 0.52% to 5.67% of individuals with CHR-P and 0.49% to 5.01% of healthy individuals; the corresponding ranges for supranormal *z* scores were 0.37% to 5.15% in individuals with CHR-P and 0.32% to 5.50% in healthy individuals. Infranormal regional SA *z* scores were noted in 0.30% to 3.66% of individuals with CHR-P and 0.65% to 3.88% of healthy individuals; the corresponding ranges for supranormal *z* scores were 1.12% to 7.01% in individuals with CHR-P and 1.21% to 6.95% in healthy individuals. Infranormal regional SV *z* scores were noted in 3.73% to 11.42% of individuals with CHR-P and 2.67% to 9.30% of healthy individuals; the corresponding ranges for supranormal *z* scores were 0.07% to 2.01% in individuals with CHR-P and 0.08% to 1.37% in healthy individuals. There were no significant group differences in the percentage of individuals with supra- or infranormal regional values (eTable 7 in Supplement 1).

**Figure 3.**
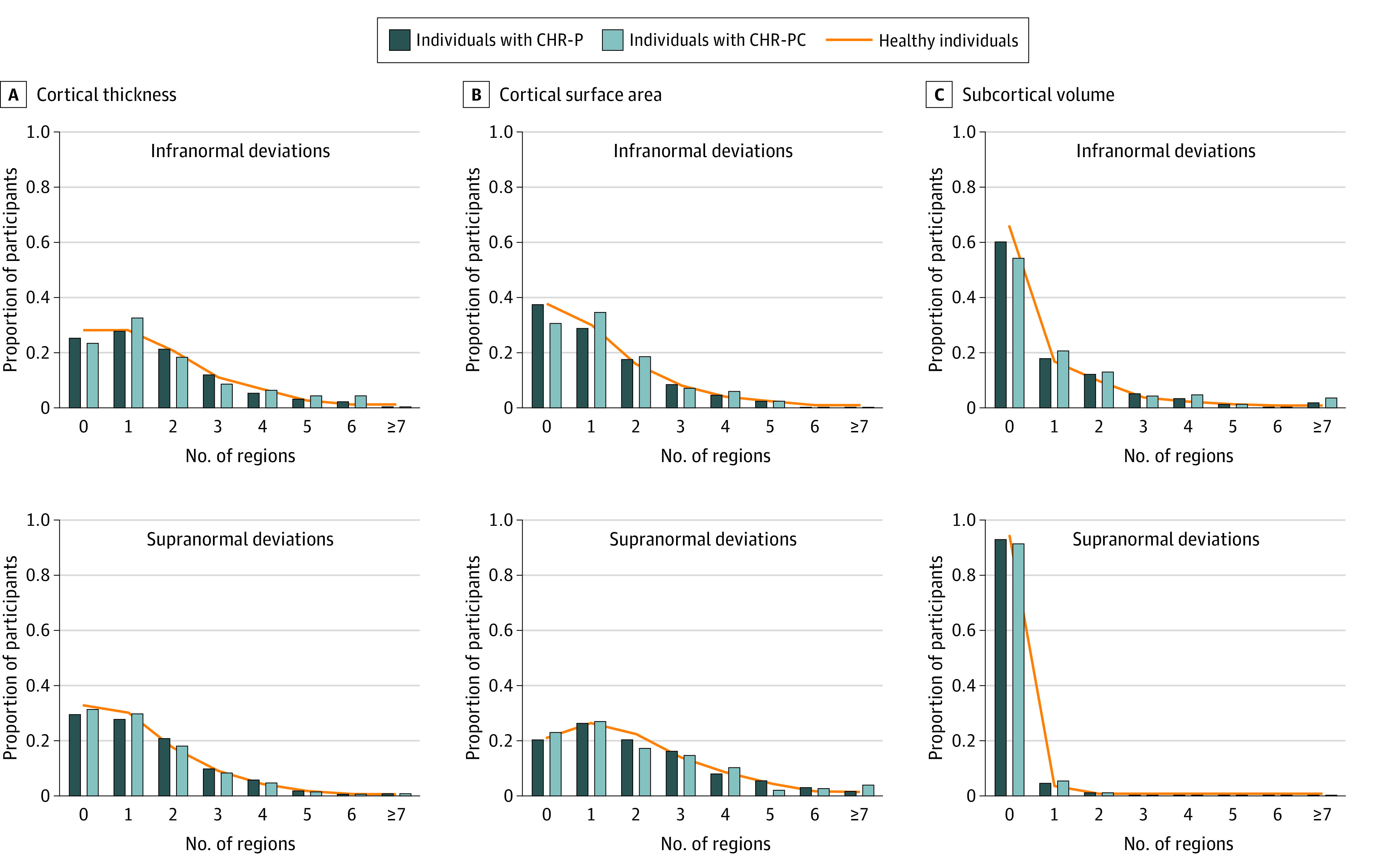
Distribution of the Total Number of Regions With Infra- or Supranormal Regional Normative *z* Scores CHR-P indicates clinical high risk for psychosis; CHR-PC, clinical high risk for psychosis converted to a psychotic disorder.

Among those with CHR-P (n = 1340), infranormal *z* scores in any regional CT were observed in 1000 individuals (74.63%), in any regional SA in 841 individuals (62.76%), and in any regional SV in 536 individuals (40.00%); the corresponding proportions of healthy individuals (n = 1237) with infranormal *z* scores were 887 (71.71%) for CT, 770 (62.25%) for SA, and 422 (34.11%) for SV (Figure 3). Supranormal *z* scores in those with CHR-P for any regional CT were observed in 943 individuals (70.37%), for any regional SA in 1068 individuals (79.70%), and for any regional SV in 89 individuals (6.64%) for any regional SV; the corresponding proportions of healthy individuals with supranormal *z* scores were 824 (66.61%) for CT, 980 (79.22%) for SA, and 69 (5.58%) for SV (Figure 3). Compared with unmedicated individuals with CHR-P, those medicated had a greater proportion with supranormal regional *z* scores for the surface area of the left lateral occipital lobe (χ^2^ = 13.92; 95% CI, −0.08 to −0.01; *P* = .03 for FDR) but no other differences (eTable 8 in Supplement 1).

In voxel-based modeling, we found that, on average, 30 548 of 439 977 voxels (6.94%) in the CHR-P sample had supranormal values, and 14 700 of 439 977 (3.34%) had infranormal *z* scores. In healthy individuals, the corresponding percentages were 9166 of 439 977 (2.08%) for supranormal *z* scores and 2441 of 439 977 (0.55%) for infranormal *z* scores. These differences were not statistically significant.

### Estimation Value of Regional *z* Scores and Observed Regional Brain Morphometric Data

**Figure 4.**
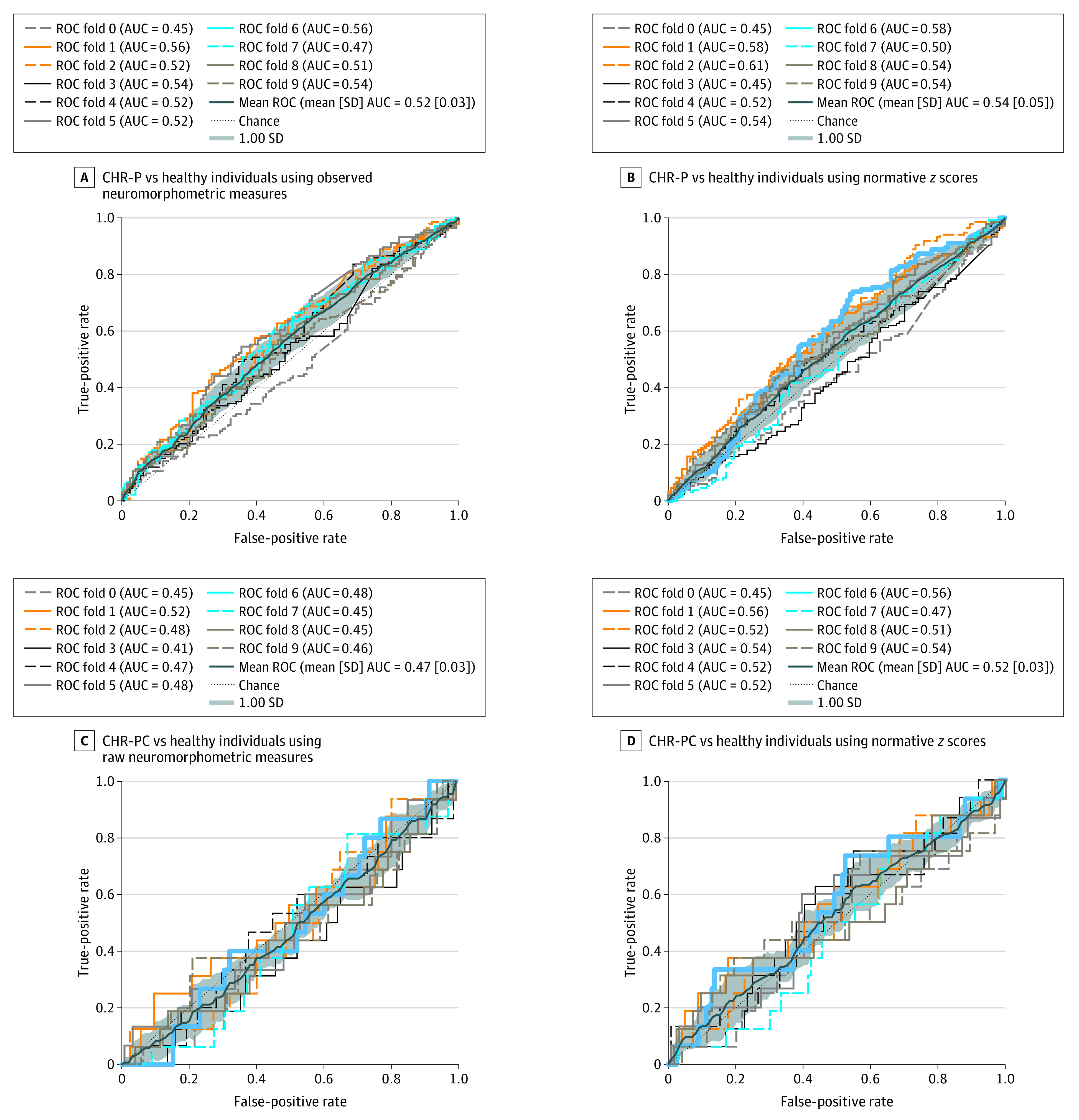
Multivariate Estimation of Group Status Using Normative Deviation Scores and Observed Values for Regional Brain Morphometric Measures Linear support vector classification was used to estimate group status. AUC indicates area under the curve; CHR-P, clinical high risk for psychosis; CHR-PC, clinical high risk for psychosis converted to a psychotic disorder; and ROC, receiver operating characteristic curve.

Linear support vector classifiers using either the observed neuromorphometric measures or the normative *z* scores were unable to distinguish healthy individuals from individuals with CHR-P (observed data area under the curve [AUC], 0.56; *z*-scores AUC, 0.53) (Figure 4A-B) or individuals with CHR-PC (observed data AUC, 0.47; *z*-scores AUC, 0.52) (Figure 4C-D).

Traditional group comparisons of the observed morphometric data identified cortical thickness decrements in individuals with CHR-P compared with healthy individuals of very small effect size (<0.22) (eTable 9 in Supplement 1). This result replicates the findings of Jalbrzikowski et al,^8^ who also used the ENIGMA CHR-P Working Group sample and reported case-control reductions of similarly small effect size in cortical thickness.

### Supra- and Infranormal Average Deviation Scores

The percentage of individuals with CHR-P and healthy individuals who had supra- or infranormal ADS values are shown in eFigure 9A and eTable 10 in Supplement 1. The distributions in both groups revealed a nearly complete overlap (eFigure 9B in Supplement 1). A significantly higher percentage of individuals with CHR-P had infranormal global ADS (χ^2^ = 12.82; 95% CI, −0.04 to −0.01; *P* = 6.85 × 10^−4^ for FDR), SV ADS (χ^2^ = 5.68; 95% CI, −0.03 to −2.71 × 10^−3^; *P* = .02 for FDR), and SA ADS (χ^2^ = 16.01; 95% CI, −0.04 to −0.01; *P* = 2.53 × 10^−4^ for FDR) (eTable 10 in Supplement 1). These findings were consistent when using alternate definitions of ADS (eTable 10 in Supplement 1). There were no differences in the percentage of individuals with CHR-P who had infra- or supranormal ADS depending on their medication status (eTable 11 in Supplement 1).

### Associations of Regional *z* Scores and Observed Values With Positive Symptoms and IQ

Within the CHR-P group, the linear models did not identify associations with positive symptoms. There were positive associations between IQ and the *z* scores of the left caudate volume (β = 0.11; 95% CI, 0.05-0.18; *P* = .05 for FDR) and surface area of the left cuneus (β = 0.11; 95% CI, 0.05-0.18; *P* = .05 for FDR) (eFigure 10A-B and eTable 12 in Supplement 1). When analyses were repeated using the observed regional morphometric values, there were no associations with IQ or positive symptoms (eTable 13 in Supplement 1). The same pattern of results for *z* scores and observed values was observed when individuals with CHR-P who were exposed to medication were excluded. In healthy individuals, no associations were noted either between IQ and *z* scores or between IQ and the observed values (eTable 14 in Supplement 1). This pattern of associations was robust to including site as a random effect in linear mixed-effects models (eTable 15 in Supplement 1).

Multivariate estimation modeling with BBS using visual examination of the scree plot to estimate the number of components yielded similar findings. The coefficient of determination (*R*^2^) and mean squared error (MSE) for positive symptoms or IQ revealed that the models using regional *z* scores were not significant (positive symptoms: *R*^2^ = −0.01; MSE = 1.00; *P* = .39; IQ: *R*^2^ = 0.03; MSE = 0.98; *P* = .64). The same was the case for models with the observed regional measures (positive symptoms: *R*^2^ = −0.01; MSE = 1.00; *P* = .42; IQ: *R*^2^ = 0.01; MSE = 1.01; *P* = .50).

### Associations of Average Deviation Scores With Positive Symptoms and IQ

Within the CHR-P group, positive symptoms were negatively associated with SA ADS (β = −0.08; 95% CI, −0.13 to −0.02; *P* = .02 for FDR) (eFigure 10C in Supplement 1); this association did not persist with the inclusion of site as a random effect (estimated β = −0.05; 95% CI, −0.11 to 4.48 × 10^−3^; *P* = .29 for FDR) (eTable 15 in Supplement 1). IQ was positively associated with SA ADS (β = 0.09; 95% CI, 0.02-0.15; *P* = .02 for FDR) and global ADS (β = 0.10; 95% CI, 0.03-0.16; *P* = .01 for FDR) (eFigure 10D-E and eTable 16 in Supplement 1). This pattern of associations was robust to medication status, leave-one-site-out analysis (eFigure 11 in Supplement 1), and inclusion of site as a random effect (SA ADS: β = 0.08 [95% CI, 0.02-0.15; *P* = .03 for FDR]; global ADS: β = 0.09 [95% CI, 0.03-0.16; *P* = .03 for FDR]) (eTable 15 in Supplement 1). In healthy individuals, no association was present between IQ and SA ADS and global ADS (eTable 16 in Supplement 1).

### Individuals With CHR-P Who Converted to a Psychotic Disorder

The percentage of individuals with CHR-PC and CHR-PNC who had infranormal and supranormal regional *z* scores and ADS are shown in eFigure 9 and eTable 17 in Supplement 1. There was a significantly greater percentage of individuals with CHR-PC (8 of 157 [5.10%]) than healthy individuals (11 of 1237 [0.89%]) with infranormal *z* scores for the thickness of the right inferior temporal lobe (χ^2^ = 15.34; 95% CI, −0.08 to −3.68 × 10^−3^; *P* = .01 for FDR), and a significantly greater percentage of individuals with CHR-PC (11 of 157 [7.01%]) than CHR-PNC (13 of 940 [1.38%]) with infranormal *z* scores for the surface area of the right bank of the superior temporal sulcus (χ^2^ = 17.34; 95% CI, 0.01-0.10; *P* = 4.69 × 10^−3^ for FDR). In individuals with CHR-PC, IQ was positively associated with SA ADS (β = 0.26; 95% CI, 0.08-0.44; *P* = .02 for FDR) and global ADS (β = 0.21; 95% CI, 0.02-0.40; *P* = .05 for FDR), even after the inclusion of site as a random effect (SA ADS: β = 0.26 [95% CI, 0.08-0.44; *P* = .02 for FDR]; global ADS: β = 0.22 [95% CI, 0.03-0.41; *P* = .04 for FDR]). No other associations were found between regional *z* scores or ADS and IQ or positive symptoms in the CHR-PC or CHR-PNC subsamples (eTable 18 in Supplement 1).

## Discussion

This case-control study found that variation in regional neuromorphometric measures in individuals with CHR-P was nested within the healthy distribution, while extreme deviations were present in a minority of individuals with CHR-P and at proportions similar to those observed in healthy individuals. However, a greater proportion of individuals with CHR-P had infranormal CT ADS, SA ADS, and SV ADS values. Additionally, a higher percentage of individuals with CHR-PC had infranormal values in temporal regions, but none of the regional *z* scores had meaningful associations with the severity of positive symptoms.

Prior case-control studies,^6,7,8^ including a study by Jalbrzikowski and colleagues,^8^ who also used the ENIGMA CHR-P Working Group data set, have reported subtle decrements in regional brain morphometry in individuals with CHR-P. Studies in patients with syndromal schizophrenia have also established the presence of reduction in measures of brain morphometry in patients compared with healthy individuals.^6,17,18^ These findings are aligned with the observation that a higher proportion of individuals with CHR-P had infranormal values for ADS in the current study. When case-control differences were reported in regional brain morphometry, either in CHR-P states or syndromal schizophrenia, their effect size was typically small (<0.3).^6,7,8,17,18^ The current findings support the notion that brain morphological changes in CHR-P states are minimal by revealing that in the majority of individuals with CHR-P, variation in regional brain morphological features was nested within the normal range. However, a small minority of patients with CHR-PC had pronounced decrements in cortical thickness and surface area of temporal regions, which may account for case-control differences in these regions in patients with psychosis risk^6,7,8^ and syndromal schizophrenia.^10,11,18^

Also using the ENIGMA CHR-P Working Group data set, Baldwin and colleagues^27^ found that individual-level heterogeneity was similar in individuals with CHR-P and healthy individuals and was not associated with increased clinical severity. In the current study, regional deviation from normative patterns in the individuals with CHR-P did not reveal associations with the severity of positive symptoms, both in linear models and multivariate estimation models. The SA ADS was positively associated with IQ both in individuals with CHR-P and in healthy individuals. The coefficient of these associations was low (β < 0.20). Nevertheless, these findings resonate with previous reports associating higher IQ with greater SA expansion^28,29^ and may reflect the integrity of neurite remodeling and intracortical myelination that determine SA expansion during early adulthood.^30,31^

### Limitations

The study includes the largest neuroimaging data set of individuals with CHR-P and robust normative models derived from an independent reference sample. As is common with large-scale studies, the data were collected at multiple sites using different scanners and protocols. Although we accounted for site effects using MRI data harmonization and tested the robustness of the results using leave-one-site-out analyses, residual effects cannot be fully excluded but are unlikely to have influenced the overall pattern of the results. The neuroimaging data of the individuals with CHR are cross-sectional and do not capture potential longitudinal changes that may be more informative.^32^

## Conclusions

In this case-control study, regional variation in the neuroanatomy of individuals with CHR-P was nested within the normal variation and had negligible estimation value for diagnosis, cognition, positive symptoms, and conversion status.

## Notes

Supplement 1. **eMethods.** Determination of Clinical High-Risk Status for Psychosis in the ENIGMA Clinical High-Risk for Psychosis Working Group, Study Sample Section and Characteristics, Structural Neuroimaging, Normative Modeling Using FreeSurfer-Derived Regional Brain Morphometric Measures, Proportion of Subjects With Infra- and Supranormal Regional Normative *z* Scores and Average Deviation Scores, Alternate Definitions of the Average Deviation Scores, Alternate Normative Models, and Prediction of IQ and Psychotic Symptoms From Regional Neuroimaging Features Using Brain Basis Set **eResults.** Associations Between Average Deviation Scores With Positive Symptoms and IQ, CHR-P Subsyndromes, Alternate Average Deviation Scores, and Prediction of IQ and Psychotic Symptoms From Regional Neuroimaging Features Using Brain Basis Set **eTable 1.** Information on the Samples Contributed by Site to the Clinical High-Risk for Psychosis Working Group of the ENIGMA Consortium **eTable 2.** SIPS and the CAARMS Items and Clinical High-Risk Criteria **eTable 3.** Neuroimaging Acquisition Protocols and FreeSurfer Version per Site **eTable 4.** Demographic Characteristics of Study Participants per Site **eTable 5.** Characteristics of Clinical High-Risk Individuals Defined With Either the SIPS or CAARMS Criteria **eTable 6.** FreeSurfer-Derived Morphometric Measures **eTable 7.** Percentage of Individuals With Infra- or Supranormal Normative Regional *z* Scores Based on Group **eTable 8.** Percentage of CHR-P With Infra- or Supranormal Normative Regional *z* Scores According to Medication Status **eTable 9.** Effect Size (Cohen *d*) of Group Differences in Observed Brain Morphometric Data **eTable 10.** Percentage of Individuals With Infra- or Supranormal Normative Average Deviation Scores by Group **eTable 11.** Percentage of CHR-P With Infra- or Supranormal Normative Average Deviation Scores According to Medication Status **eTable 12.** Associations Between Regional Normative *z* Scores and Positive Symptoms and IQ in CHR-P Individuals **eTable 13.** Associations Between Observed Brain Morphometric Measures With Positive Symptoms and IQ in CHR-P **eTable 14.** Associations Between Either Regional *z* Scores or Observed Brain Morphometric Measures With IQ in Healthy Individuals **eTable 15.** Associations Between Regional *z* Scores and Average Deviation Scores With Positive Symptoms and IQ in CHR-P Adding Site as a Random Effect **eTable 16.** Associations Between Average Deviation Scores With Positive Symptoms and IQ in CHR-P and Healthy Individuals **eTable 17.** Percentage of CHR-P With Infra- or Supranormal Normative Regional *z* Score According to Conversion Status **eTable 18.** Associations Between Regional *z* Scores and Average Deviation Scores With Positive Symptoms and IQ Based on Conversion Status **eFigure 1.** Flow Diagram for Study Sample Selection of Individuals at Clinical High-Risk for Psychosis (CHR-P) and Healthy Individuals (HI) From the Sample Available Through the ENIGMA CHR-P Working Group **eFigure 2.** Age Distribution in the Entire Sample and Within Each Site **eFigure 3.** Computation of Regional Normative Deviation Scores (*z* Scores) **eFigure 4.** Distribution of Normative Regional *z* Scores for FreeSurfer-Derived Brain Morphometric Measures in CHR-P and Healthy Individuals in the Entire Study Sample **eFigure 5.** Distribution of Normative Regional *z* Scores and Average Deviation Scores of Freesurfer-Derived Brain Morphometric Measures in CHR-P and Healthy Individuals by Site **eFigure 6.** Distribution of Normative Regional *z* Scores for Cortical Thickness Based on the Schaefer 400 Parcellation Distribution for the Left (Panel A) and the Right Hemisphere (Panel B) **eFigure 7.** Distribution of Normative Regional *z* Scores for Cortical Surface Area Based on the Schaefer 400 Parcellation for the Left (Panel A) and the Right Hemisphere (Panel B) **eFigure 8.** Percentage of subjects with infra- or supranormal regional normative *z* -scores for subcortical volume **eFigure 9.** Distributions of the Average Deviation Scores and Percentage of Subjects With Infra- or Supranormal Regional Normative *z* Scores **eFigure 10.** Associations Between Regional and Average Deviation Scores and Clinical Measures in CHR-P Individuals **eFigure 11.** Associations Between Average Deviation Scores With the Positive Symptoms and IQ Based on Medication Exposure and Removing 1 Site at a Time
eReferences

Supplement 2. **Nonauthor Collaborators.** The ENIGMA Clinical High Risk for Psychosis Working Group Collaborators

Supplement 3.
Data Sharing Statement
