# Supplementary material for: Normative Modeling of Brain Morphometry in Clinical High Risk for Psychosis
Source: JAMA Psychiatry. 2023 Oct 11;81(1):77–88. doi: 10.1001/jamapsychiatry.2023.3850 (PMC10568447; doi:10.1001/jamapsychiatry.2023.3850)
Supplement: Supplement 1. — eMethods. Determination of Clinical High-Risk Status for Psychosis in the ENIGMA Clinical High-Risk for Psychosis Working Group, Study Sample Section and Characteristics, Structural Neuroimaging, Normative Modeling Using FreeSurfer-Derived Regional Brain Morphometric Measures, Proportion of Subjects With Infra- and Supranormal Regional Normative z Scores and Average Deviation Scores, Alternate Definitions of the Average Deviation Scores, Alternate Normative Models, and Prediction of IQ and Psychotic Symptoms From Regional Neuroimaging Features Using Brain Basis Set eResults. Associations Between Average Deviation Scores With Positive Symptoms and IQ, CHR-P Subsyndromes, Alternate Average Deviation Scores, and Prediction of IQ and Psychotic Symptoms From Regional Neuroimaging Features Using Brain Basis Set eTable 1. Information on the Samples Contributed by Site to the Clinical High-Risk for Psychosis Working Group of the ENIGMA Consortium eTable 2. SIPS and the CAARMS Items and Clinical High-Risk Criteria eTable 3. Neuroimaging Acquisition Protocols and FreeSurfer Version per Site eTable 4. Demographic Characteristics of Study Participants per Site eTable 5. Characteristics of Clinical High-Risk Individuals Defined With Either the SIPS or CAARMS Criteria eTable 6. FreeSurfer-Derived Morphometric Measures eTable 7. Percentage of Individuals With Infra- or Supranormal Normative Regional z Scores Based on Group eTable 8. Percentage of CHR-P With Infra- or Supranormal Normative Regional z Scores According to Medication Status eTable 9. Effect Size (Cohen d) of Group Differences in Observed Brain Morphometric Data eTable 10. Percentage of Individuals With Infra- or Supranormal Normative Average Deviation Scores by Group eTable 11. Percentage of CHR-P With Infra- or Supranormal Normative Average Deviation Scores According to Medication Status eTable 12. Associations Between Regional Normative z Scores and Positive Symptoms and IQ in CHR-P Individuals eTable 13. Associatio [file jamapsychiatry-e233850-s001.pdf]

Supplemental Online Content

ENIGMA Clinical High Risk for Psychosis Working Group. Normative modeling of brain morphometry in clinical high risk for psychosis. *JAMA Psychiatry*. Published online October 11, 2023.
doi:10.1001/jamapsychatry.2023.3850

eMethods. Determination of Clinical High-Risk Status for Psychosis in the ENIGMA Clinical High-Risk for Psychosis Working Group, Study Sample Section and Characteristics, Structural Neuroimaging, Normative Modeling Using FreeSurfer-Derived Regional Brain Morphometric Measures, Proportion of Subjects With Infra- and Supranormal Regional Normative *z* Scores and Average Deviation Scores, Alternate Definitions of the Average Deviation Scores, Alternate Normative Models, and Prediction of IQ and Psychotic Symptoms From Regional Neuroimaging Features Using Brain Basis Set

eResults. Associations Between Average Deviation Scores With Positive Symptoms and IQ, CHR-P Subsyndromes, Alternate Average Deviation Scores, and Prediction of IQ and Psychotic Symptoms From Regional Neuroimaging Features Using Brain Basis Set

eTable 1. Information on the Samples Contributed by Site to the Clinical High-Risk for Psychosis Working Group of the ENIGMA Consortium

eTable 2. SIPS and the CAARMS Items and Clinical High-Risk Criteria

eTable 3. Neuroimaging Acquisition Protocols and FreeSurfer Version per Site

eTable 4. Demographic Characteristics of Study Participants per Site

eTable 5. Characteristics of Clinical High-Risk Individuals Defined With Either the SIPS or CAARMS Criteria

eTable 6. FreeSurfer-Derived Morphometric Measures

eTable 7. Percentage of Individuals With Infra- or Supranormal Normative Regional *z* Scores Based on Group

eTable 8. Percentage of CHR-P With Infra- or Supranormal Normative Regional *z* Scores According to Medication Status

eTable 9. Effect Size (Cohen *d*) of Group Differences in Observed Brain Morphometric Data

eTable 10. Percentage of Individuals With Infra- or Supranormal Normative Average Deviation Scores by Group

eTable 11. Percentage of CHR-P With Infra- or Supranormal Normative Average Deviation Scores According to Medication Status

eTable 12. Associations Between Regional Normative *z* Scores and Positive Symptoms and IQ in CHR-P Individuals

eTable 13. Associations Between Observed Brain Morphometric Measures With Positive Symptoms and IQ in CHR-P

eTable 14. Associations Between Either Regional *z* Scores or Observed Brain Morphometric Measures With IQ in Healthy Individuals

eTable 15. Associations Between Regional *z* Scores and Average Deviation Scores With Positive Symptoms and IQ in CHR-P Adding Site as a Random Effect

eTable 16. Associations Between Average Deviation Scores With Positive Symptoms and IQ in CHR-P and Healthy Individuals

© 2023 American Medical Association. All rights reserved.

eTable 17. Percentage of CHR-P With Infra- or Supranormal Normative Regional *z* Score According to Conversion Status

eTable 18. Associations Between Regional *z* Scores and Average Deviation Scores With Positive Symptoms and IQ Based on Conversion Status

eFigure 1. Flow Diagram for Study Sample Selection of Individuals at Clinical High-Risk for Psychosis (CHR-P) and Healthy Individuals (HI) From the Sample Available Through the ENIGMA CHR-P Working Group

eFigure 2. Age Distribution in the Entire Sample and Within Each Site

eFigure 3. Computation of Regional Normative Deviation Scores (*z* Scores)

eFigure 4. Distribution of Normative Regional *z* Scores for FreeSurfer-Derived Brain Morphometric Measures in CHR-P and Healthy Individuals in the Entire Study Sample

eFigure 5. Distribution of Normative Regional *z* Scores and Average Deviation Scores of Freesurfer-Derived Brain Morphometric Measures in CHR-P and Healthy Individuals by Site

eFigure 6. Distribution of Normative Regional *z* Scores for Cortical Thickness Based on the Schaefer 400 Parcellation Distribution for the Left (Panel A) and the Right Hemisphere (Panel B)

eFigure 7. Distribution of Normative Regional *z* Scores for Cortical Surface Area Based on the Schaefer 400 Parcellation for the Left (Panel A) and the Right Hemisphere (Panel B)

eFigure 8. Percentage of subjects with infra- or supranormal regional normative z-scores for subcortical volume

eFigure 9. Distributions of the Average Deviation Scores and Percentage of Subjects With Infra- or Supranormal Regional Normative *z* Scores

eFigure 10. Associations Between Regional and Average Deviation Scores and Clinical Measures in CHR-P Individuals

eFigure 11. Associations Between Average Deviation Scores With the Positive Symptoms and IQ Based on Medication Exposure and Removing 1 Site at a Time

eReferences

This supplementary material has been provided by the authors to give readers additional information about their work.

© 2023 American Medical Association. All rights reserved.

eMethods. Determination of Clinical High-Risk Status for Psychosis in the ENIGMA Clinical High-Risk for Psychosis Working Group, Study Sample Section and Characteristics, Structural Neuroimaging, Normative Modeling Using FreeSurfer-Derived Regional Brain Morphometric Measures, Proportion of Subjects With Infra- and Supranormal Regional Normative *z* Scores and Average Deviation Scores, Alternate Definitions of the Average Deviation Scores, Alternate Normative Models, and Prediction of IQ and Psychotic Symptoms From Regional Neuroimaging Features Using Brain Basis Set

For the current study sample, both CHR-P and healthy individuals were selected from the total sample available through the sites participating in the ENIGMA Clinical High Risk for Psychosis Working Group.

Determination of Clinical High-Risk status for Psychosis in the ENIGMA Clinical High-Risk for Psychosis Working Group

The criteria for participant selection at each site are showin in eTable 1. Clinical high-risk (CHR-P) status for Psychosis in each recruitment site was determined using either the Comprehensive Assessment of At-Risk Mental States (CAARMS)[1] or the Structured Interview for Prodromal Syndromes/Psychosis-Risk Syndromes (SIPS)[2,3]. Both are semi-structured interviews conducted by trained clinicians. The items of the instruments and the criteria required to meet CHR status are described in eTable 2.

The CAARMS consists of seven subscales: positive symptoms, cognitive change, emotional disturbances, negative symptoms, behavioral change, motor change, and general psychopathology. The positive symptoms subscale and an assessment of social and occupational functioning are used to determine CHR-P eligibility and syndrome subgroup. The positive symptoms subscale consists of four positive symptoms. Each symptom is rated on a scale from 0 (absent) to 6 (endorsed at a fully psychotic level). Based on different criteria (eTable 2) a CHR-P participant could be classified as having either attenuated psychotic symptoms (APS) or brief limited intermittent psychotic symptoms (BIPS) or a "trait/vulnerability" syndrome.

The SIPS has four subscales: positive, negative, disorganization, and general symptoms. The positive symptoms subscale was used to determine CHR-P eligibility and consists of five positive symptoms. Each symptom is rated on a scale from 0-6 as for the CAARMS. Based on different criteria (eTable 2) a participant could be classified as having APS, BIPS or a "genetic risk and deterioration" (GRD) syndrome (equivalent to the trait/vulnerability syndrome).

Study Sample Section and Characteristics

Participants that were in the current study sample were selected based on the availability of high-quality imaging data (<5% of missing regional measures or intracranial volume values per individual scan) and completed CAARMS or SIPS ratings. The flow diagram in eFigure 1 shows the selection process for the current study sample. eTable 3 presents the demographic breakdown of the study sample by site and eFigure2 the age distribution in the entire study sample and in each site separately. Of the 1340 CHR individuals, 806 (60.15%) were assessed using SIPS and 534 (39.85%) using CAARMS. Their clinical characteristics are presented in eTable 4.

Structural Neuroimaging

At each recruitment site, participants underwent structural magnetic resonance imaging (sMRI) to obtain T1-weighted scans. Details of the acquisition protocols and FreeSurfer version per site are shown in eTable 5. Images were parcellated and segmented at each respective site using standard pipelines implemented using FreeSurfer analysis software (https://surfer.nmr.mgh.harvard.edu/) to yield regional measures of cortical thickness (CT), cortical surface area (SA), and subcortical volume (SV) (eTable 6). Quality assessment of the FreeSurfer output used standardized ENIGMA procedures (http://enigma.ini.usc.edu/protocols/imaging-protocols/).

Normative modeling using FreeSurfer-derived regional brain morphometric measures

We used the CentileBrain framework (https://centilebrain.org/) to generate sex-specific normative models for each of the FreeSurfer-extracted regional measure of cortical thickness (CT), Surface Area (SA), and Subcortical Volume (SV) (eTable 4) estimated using Multivariate Fractional Polynomial Regression (MFPR)[4] in a sample of 37,407 healthy individuals (53.3% female) with an age range of 3-90 years pooled from 81 datasets. Normative models for each morphometric measures were estimated using the following procedures: (i) data preparation: Sex-specific subsamples of each pooled sample were randomly split into a training subset (80%) and a test subset (20%) stratified by scanning site. In each subset, data were mean-centered after extreme values in each site-dataset were identified and removed using the interquartile range (IQR) method; (ii) site harmonization was implemented using ComBat-GAM[5]; (iii) normative model of each neuroimaging measure were generated using MFPR implemented using the "mfp" package in R and the closed test

© 2023 American Medical Association. All rights reserved.

procedure (known as RA2) to select the most appropriate fractional polynomial. Initially, models were trained using 5-fold cross-validation (5F-CV) in the corresponding sex-specific training subset with age being the only explanatory variable and then model parameters were tested in the corresponding sex-specific test subset. Model performance was evaluated using the Mean Absolute Error (MAE) and Root Mean Square Error (RMSE); the MAE is the average of the absolute differences (i.e., errors) between the predicted and the actual value of the outcome variable and the RMSE is the standard deviation of the prediction errors (iv) model optimization involves the inclusion in the models of covariates pertaining to acquisition and global neuroimaging features (i.e., ICV for SV models, mean cortical thickness for CT models and mean cortical surface area for SA models.

For each FreeSurfer-extracted measure, individualized z-scores were generated by subtracting the predicted value ($\hat{Y}$) from the observed value (Y_o) of that measure divided by the RMSE of the model (eFigure 3)[6-8].

Proportion of subjects with infra- and supranormal regional normative z-scores and average deviation scores

In order to identify the proportion of CHR-P with infra- and supranormal regional z-scores, for example, the percentage of CHR-P individuals with supranormal values for a region X was computed as: [(N of CHR-P individuals with supranormal values in X/N of CHR-P individuals)*100]. The same method was applied to identify the proportion of CHR-P with infranormal regional z-scores. Similarly, in order to identify the proportion of CHR-P with infra- or supranormal average deviation scores (ADS), for example, the percentage of CHR-P individuals with supranormal ADS_{CT} was computed as: [(N of CHR-P individuals with supranormal ADSCT values/N of CHR-P individuals)*100]. This method was applied to identify both infra- and supranormal ADS for CT (ADS_{CT}), SA (ADS_{SA}), SV(ADS_{SV}), and globally across all measures (ADS_{G}). The same was repeated in for all measures in healthy individuals.

Alternate definitions of the average deviation scores

We computed alternate average deviation scores for each neuroimaging phenotype and a global index based on the sign of the regional z-scores. Accordingly, regions with positive z-scores for cortical thickness were averaged to form the positive ASD_{CT} (P-ASD_{CT}), and regions with negative z-scores for cortical thickness were averaged to form the negative ASD_{CT} (N-ASD_{CT}). The same process was used to compute the P-ASD_{SA} and N-ASD_{SA} for cortical surface area, the P-ASD_{SV} and N-ASD_{SV} for the subcortical volumes, and P-ADS_{G} and N-ADS_{G} globally across all measures. Four additional alternate measures we computed using the sum of the |absolute values of z-scores which we termed $|ASD_{G}|$, $|ASD_{CT}|$, $|ASD_{SA}|$. and $|ASD_{SV}|$.

Alternate normative models

MFPR models with Schaeffer Parcellation: These normative models were generated using the same procedures described in the main analyses in a subset with raw T1-weighted structural images available but the FreeSurfer-derived metrics were substituted by the Schaefer scheme parcels[9] (https://github.com/ThomasYeoLab/CBIG/tree/master/stable_projects/brain_parcellation/Schaefer2018_LocalGlobal/Parcellations/project_to_individual) and using the *mri_anatomical_stats function* to extract cortical values. We used the 400-parcel resolution (i.e., 400 cortical thickness and 400 cortical surface area parcels) which has been shown to have good signal-to-noise ratio and correspondence to histology[10-11].

GAMLSS models with FreeSurfer-derived measures: The Generalized Additive Models for Location, Scale, and Shape can model heteroskedasticity, non-linear effects of variables, and hierarchical structure of the data. This algorithm was chosen because of its popularity in normative modeling studies and was implemented using the "caret" package in R following prior recommendations[12]. The procedures for generating these models were the same as for the MFPR models.

Voxel-Based model with Gaussian Process Regression: This is a nonparametric regression model that follows Bayesian principles. GPR models have been advocated by other researchers. Implementation followed the procedures described by Wolfers et al, 2018[13]. Briefly, in a subset of the sample with available raw structural MRI, images were preprocessed using the computational analysis toolbox version 12 (CAT12; http://www.neuro.uni-jena.de/software/). The gray and white matter VBM maps were used as input features in a GPR model implemented using the "kernlab" package in R and the "sigest" function for estimating the hyperparameter sigma with 10-fold cross-validation. Across all alternative models infra-normal and supernormal values were defined based on $|z|=1.96$ as in the main analyses.

© 2023 American Medical Association. All rights reserved.

Prediction of IQ and psychotic symptoms from regional neuroimaging features using Brain Basis Set

The Brain Basis Set[14] is a multivariate predictive model that has been previously used to study the association of cognitive measures with neuroimaging features[15]. It decomposes neuroimaging features (here regional z-scores and observed regional brain morphometry measures) into components that serve as input features to the predictive model. Here we estimated the number of components using two methods: 1) the visual examination of a scree plot, identifying the elbow or the point at which the proportion of variance explained by each subsequent principal component drops off (results reported in main manuscript), and 2) Kaiser's rule (Kaiser-Guttman criterion) in which only principal components whose variances exceed one were retained (results reported in the supplement). For the visualization of the scree plot method, we identified 3 components for observed data and 5 components for normative data (z-scores). Using Kaiser's rule, we identified 25 components for observed and 47 components for normative data. Predictive modeling implemented in Python v3.8 were conducted using all plausible component numbers. Model performance metrics included the coefficient of determination (R^2 score), which measures the proportion of the variance in the dependent variable that is predictable from the independent variables and Mean Squared Error which measures the average squared difference between the actual (observed) values and the predicted values.

© 2023 American Medical Association. All rights reserved.

eResults. Associations Between Average Deviation Scores With Positive Symptoms and IQ, CHR-P Subsyndromes, Alternate Average Deviation Scores, and Prediction of IQ and Psychotic Symptoms From Regional Neuroimaging Features Using Brain Basis Set

Associations between average deviation scores with positive symptoms and IQ

The association between ADSs with positive symptoms and IQ in CHR-P individuals was not influenced by medication status (eFigure 11A and B). The leave-one-site-out analyses replicated these findings with two exceptions: the association between ADS_{SA} with positive symptoms was not present after excluding the Copenhagen site (11.79% of the CHR sample) and the association between ADS_{SA} and IQ was not present after excluding the UCSF site (4.8% of the CHR sample) (eFigure 11C and D).

CHR-P subsyndromes

Of the 1340 CHR-P individuals, 1057 were classified as having attenuated symptoms (APS), 28 as having brief limited intermittent symptoms (BIPS) and 53 as having genetic risk and deterioration syndrome (GRD). As the sample size for BIPS and GRD was too small, the analyses described in the main manuscript were repeated for those with APS. The results recapitulated those reported for the total sample.

Alternate average deviation scores

We computed alternate average deviations scores by averaging separately positive, negative, or the absolute value for regional z-scores for the imaging phenotype (eMethods). We compared the proportion of HI and CHR-P with infranormal and supranormal deviations using the alternative definitions (eTable 10) and computed associations between each of these ADS and the positive symptom scores and IQ (eTable 15).

Prediction of IQ and psychotic symptoms from regional neuroimaging features using Brain Basis Set

Multivariate predictive modeling with BBS using Kaiser's rule to estimate the number of components yielded similar findings. The coefficient of determination (R2) and Mean Squared Error (MSE) for positive symptoms or IQ indicated the models using regional z-scores were negligible (positive symptoms: R2 = -0.04; MSE = 1.03; IQ: R2 = 0.07; MSE = 0.95, both P>0.05). The same was the case for models with the observed regional measures (positive symptoms: R2 = -0.03; MSE = 1.02; IQ: R2 = 0.03; MSE = 0.99, both P>0.05).

© 2023 American Medical Association. All rights reserved.

eTable 1. Information on the Samples Contributed by Site to the Clinical High-Risk for Psychosis Working Group of the ENIGMA Consortium

#	Full Site Name	Abbreviated Site Name	Major Eligibility Criteria for Healthy and CHR-P participants	Site sample (N) and mean age (SD) in years	Criteria for CHR-P status	Inclusion Criteria for Healthy Participants	IQ Method
**1**	Vrije Universiteit Amsterdam, The Netherlands	Amsterdam	• 16-31 years<br>• Sufficient command of the Dutch language<br>• IQ ≥80	Healthy=23; 23.45 (2.80) CHR-P=16; 23.64 (2.51)	• Met CAARMS criteria	• CAARMS and SOFAS score <55<br>• No family history of psychiatric disorders	NA
**2**	New York State Psychiatric Institute, Columbia University, USA	Columbia1	• 15-35 years<br>• No major medical or neurological disorder diagnosis<br>• IQ >70<br>• No lifetime substance use disorder<br>• No lifetime history of traumatic head injury<br>• No imminent risk of harm to self or others	Healthy=9; 24.35 (4.05) CHR-P=17; 22.89 (4.96)	• Met SIPS criteria	• No SCID Axis I and/or Axis II Cluster C disorder diagnosis<br>• Participants Not adopted	NA
**3**	New York State Psychiatric Institute, Columbia University, USA	Columbia3	• 14-30 years<br>• No lifetime major medical or neurological disorders<br>• IQ >70	Healthy=17; 22.91 (3.68) CHR-P=53; 21.27 (4.01)	• Met SIPS criteria<br>• CHR symptoms do not occur solely in the context of substance use	• No lifetime psychiatric disorder diagnosis<br>• No lifetime history of meeting criteria for psychosis-risk syndrome via the SIPS<br>• No current substance abuse	NA
**4**	Mental Health Center Copenhagen and CINS, Mental Health Center Glostrup, University of Copenhagen, Denmark	Copenhagen_1 Copenhagen_2	• 18-40 years<br>• No organic brain disorder diagnosis (e.g., epilepsy, inflammatory brain disease)<br>• Fluent in Danish<br>• IQ >70<br>• Not currently pregnant	Healthy=58; 24.78 (3.30) CHR-P=158; 24.21 (4.22)	• Met CAARMS criteria<br>• No psychiatric symptoms that could be explained by a physical illness or acute drug intoxication<br>• No diagnosis of a serious developmental disorder (e.g. Asperger's syndrome)<br>• Lifetime neuroleptic	• No lifetime DSM-IV psychiatric disorder diagnosis	WAIS-III

© 2023 American Medical Association. All rights reserved.

#	Full Site Name	Abbreviated Site Name	Major Eligibility Criteria for Healthy and CHR-P participants	Site sample (N) and mean age (SD) in years	Criteria for CHR-P status	Inclusion Criteria for Healthy Participants	IQ Method
					exposure equivalents less than haloperidol dose of >50 mg<br>• No current mood stabilizer use or recreational use of ketamine		
**5**	Central South University, China	CSU	• 13-30 years<br>• No lifetime neurological disease or organic brain abnormalities (examined by neuroradiologist blinded to group allocation)<br>• Fluent in Chinese<br>• IQ >70<br>• Not currently pregnant<br>• No lifetime drug or alcohol dependence	Healthy=55; 21.47 (3.20)<br>CHR=49; 19.49 (5.05)	• Met SIPS criteria<br>• No lifetime psychotropic medication use		WISC-I+PC
**6**	University Hospital of Child and Adolescent Psychiatry and Psychotherapy, University of Bern, Switzerland	FETZ Bern	• 8-40 years<br>• No current neurological disorder diagnosis<br>• Fluent in German, French or English<br>• IQ >70 (via clinical impression)	Healthy=15; 20.11 (6.02)<br>CHR-P=39; 19.14 (4.81)	• Met SIPS criteria	• No lifetime psychotic disorder diagnosis	NA
**7**	Institute of Neuroscience and Psychology, University of Glasgow, Scotland	Glasgow	• 16-35 years<br>• No organic cause for presentation<br>• Fluent in English<br>• IQ >70	Healthy=45; 22.89 (3.61)<br>CHR-P=75; 22.45 (4.82)	• Met CAARMS criteria	• No current psychiatric disorder diagnosis<br>• No current substance abuse<br>• No first-degree relative with a psychotic disorder diagnosis	NART

© 2023 American Medical Association. All rights reserved.

#	Full Site Name	Abbreviated Site Name	Major Eligibility Criteria for Healthy and CHR-P participants	Site sample (N) and mean age (SD) in years	Criteria for CHR-P status	Inclusion Criteria for Healthy Participants	IQ Method
**8**	Heidelberg University Hospital, Germany	Heidelberg	• 14-18 years<br>• No lifetime neurological disorder diagnosis<br>• Fluent in German<br>• IQ >85<br>• No illegal drug use in past 12 months	Healthy=31; 15.71 (0.90)<br>CHR-P=22; 15.14 (1.08)	• Met SIPS criteria<br>• No lifetime DSM-IV Axis I diagnosis<br>• No psychotropic medication exposure	• No lifetime DSM-IV Axis-I disorder diagnosis<br>• No psychotropic medication prescription<br>• Does not meet APS psychosis risk syndrome via SIPS	WISC-IV
**9**	August Pi i Sunyer Biomedical Research Institute, Barcelona, Spain	IDIBAPS	• 10-17 years<br>• No lifetime neurological disorder or traumatic brain injury diagnosis<br>• Fluent in Spanish or Catalan<br>• IQ >70	Healthy=44; 16.00 (1.53)<br>CHR-P=60; 15.32 (1.80)	• Met SIPS criteria<br>• No autism spectrum disorder	• No current psychiatric diagnosis<br>• No current psychotropic medication use<br>• No lifetime psychotic disorder<br>• No first- or second-degree relative with psychotic disorder diagnosis	WAIS-III<br>WAIS-IV<br>WISC-IV<br>WISC-V
**10**	Icahn School of Medicine at Mount Sinai, USA	ISMMS	15-35 years<br>• No major medical or neurological disorder diagnosis<br>• No lifetime traumatic head injury<br>• Fluent in English<br>• IQ >70<br>• No MRI contraindications<br>• No lifetime substance use disorder<br>• No imminent risk of harm to self or others	Healthy=11; 27.89 (3.95)<br>CHR-P=24; 23.43 (5.55)	• Met SIPS criteria	• No SCID Axis I diagnosis, Axis II Cluster C disorder<br>• Participants not adopted	NA

© 2023 American Medical Association. All rights reserved.

#	Full Site Name	Abbreviated Site Name	Major Eligibility Criteria for Healthy and CHR-P participants	Site sample (N) and mean age (SD) in years	Criteria for CHR-P status	Inclusion Criteria for Healthy Participants	IQ Method
**11**	Institute of Psychiatry, Psychology & Neuroscience, King's College London, UK	London	• 18-35 years<br>• No lifetime neurological disorder diagnosis<br>• Fluent in English<br>• IQ within normal range<br>• No drug or alcohol dependence, as specified in DSM-IV<br>• No recreational drug use in past two weeks	Healthy=12; 26.42 (4.78)<br>CHR-P=46; 23.52 (4.64)	• Met CAARMS criteria	• No lifetime psychiatric disorder diagnosis<br>• No current psychotropic medication use	NA
**12**	Maastricht University, The Netherlands	Maastricht	• 12-55 years<br>• No lifetime neurological disorder diagnosis<br>• Fluent in Dutch<br>• IQ >70	Healthy=37; 25.52 (5.73)<br>CHR-P=39; 20.13 (4.18)	• Met SIPS criteria<br>• No lifetime psychotic disorder for more than one week, based on DSM-IV criteria<br>• Clear evidence that the psychosis-risk syndrome is not due to a non-schizophrenia-spectrum psychiatric disorder	• No DSM-IV psychiatric disorder diagnosis (lifetime)<br>• No psychotropic medication prescription (lifetime)<br>• No first- or second-degree relative with a psychotic disorder diagnosis	DART
**13**	University of Melbourne, Australia	Melbourne	• 14-30 years<br>• No history of substantial head injury, seizures, neurologic diseases, impaired thyroid function, corticosteroid use<br>• Fluent in English<br>• No alcohol or substance abuse or dependence	Healthy=90; 21.76 (3.59)<br>CHR-P=18; 18.39 (2.87)	• Met CAARMS criteria<br>• No known organic cause for presentation<br>• Lifetime neuroleptic exposure is less than total lifetime haloperidol use of >15mg	• No lifetime psychiatric disorder diagnosis<br>• No family history of psychotic illness	WASI<br>WASI-II

© 2023 American Medical Association. All rights reserved.

#	Full Site Name	Abbreviated Site Name	Major Eligibility Criteria for Healthy and CHR-P participants	Site sample (N) and mean age (SD) in years	Criteria for CHR-P status	Inclusion Criteria for Healthy Participants	IQ Method
**14**	Instituto Nacional de Neurología y Neurocirugía, Mexico City, Mexico	Mexico City	• 13-34 years<br>• No lifetime neurological disorder or traumatic brain injury diagnosis<br>• No lifetime drug or alcohol dependence	Healthy=37; 20.97 (3.4)<br>CHR-P=30; 19.7 (4.22)	• Met SIPS criteria	• No lifetime diagnosis of any psychiatric disorder<br>• No first- or second-degree relative with psychotic disorder	NA
**15**	Mental Health Research Center Moscow, Russia	MHRC	• 16-28 years<br>• Male<br>• Right-handed<br>• No lifetime neurological disorder diagnosis<br>• No lifetime drug or alcohol dependence	Healthy=33; 22.52 (2.52)<br>CHR=18; 20.03 (2.61)	• Met SIPS criteria<br>• No lifetime mental or behavioural disorders due to psychoactive substance use	• No lifetime psychiatric disorder<br>• No lifetime psychotropic medication prescription<br>• No family history of psychiatric or neurological disorders	NA
**16**	Maryland Psychiatric Research Center, University of Maryland School of Medicine, USA	MPRC	• 15-35 years<br>• No major medical or neurological disorder diagnosis<br>• No lifetime traumatic head injury<br>• Fluent in English<br>• IQ >70<br>• No MRI contraindications<br>• No lifetime substance use disorder<br>• No imminent risk of harm to self or others	Healthy=19; 18.00 (4.28)<br>CHR=29; 17.28 (3.21)	• Met SIPS criteria	• No lifetime psychiatric disorder	WASI<br>WASI-II
**17**	University of Newcastle, Australia	Newcastle	• 13-25 years<br>• No antipsychotic pharmacotherapy<br>• IQ ≥70<br>• No lifetime drug abuse or dependence diagnosis	Healthy=17; 20.25 (1.91)<br>CHR-P=43; 19.53 (2.12)	• Met CAARMS criteria<br>• Drop of 30% in GAF in the past 12 months	• No psychiatric disorder diagnosis<br>• No lifetime treatment for depression or anxiety<br>• No first-degree family member with schizophrenia	WASI-II

© 2023 American Medical Association. All rights reserved.

#	Full Site Name	Abbreviated Site Name	Major Eligibility Criteria for Healthy and CHR-P participants	Site sample (N) and mean age (SD) in years	Criteria for CHR-P status	Inclusion Criteria for Healthy Participants	IQ Method
			• No head injury with loss of consciousness (>15 mins)<br>• No organic brain impairment<br>• No hearing impairment (>20dB SPL)<br>• No history of nasal trauma.				
**18**	NORMENT, University of Oslo and Oslo University Hospital, Norway	Oslo Region	• 15-30 years<br>• No organic cause for presentation<br>• Fluent in Norwegian<br>• IQ >70	Healthy=62; 19.90 (3.62)<br>CHR-P=20; 20.08 (3.61)	• Met SIPS criteria<br>• No antipsychotic medication equivalent to a dose of ≥5 mg Olanzapine per day (current or ≥4 weeks lifetime)<br>• CHR symptoms not substance-induced	• No lifetime psychiatric disorder diagnosis<br>• No lifetime psychotropic medication use<br>• No lifetime psychotic disorder diagnosis<br>• No first-degree relative with psychotic disorder diagnosis<br>• No lifetime substance abuse disorder diagnosis	WASI
**19**	University of Pittsburgh, USA	Pitt	• 12-40 years<br>• No lifetime neurological disorder diagnosis<br>• Fluent in English<br>• IQ >80	Healthy=60; 22.93 (5.53)<br>CHR-P=25; 20.87 (5.4)	• Met SIPS criteria<br>• psychosis-risk syndrome not due to a non-schizophrenia-spectrum psychiatric disorder	• No lifetime disorder diagnosis<br>• No lifetime psychotropic medication exposure<br>• No lifetime psychosis risk syndrome (SIPS criteria)<br>• No first-degree relative with a psychotic disorder diagnosis	WASI
**20**	Rush University Medical Center, Chicago, IL USA	RUMC	• 12-35 years<br>• No lifetime major medical illness or neurological disorder diagnosis that could confound behavioral or	Healthy=29; 23.88 (3.22)<br>CHR-P=62; 22.74 (3.93)	• Met SIPS criteria<br>• Psychosis-risk syndrome not due to a non-schizophrenia-spectrum psychiatric disorder	• No current Axis I/II disorders<br>• Positive symptoms rated 1 or lower on the SIPS	WASI<br>WRAT

© 2023 American Medical Association. All rights reserved.

#	Full Site Name	Abbreviated Site Name	Major Eligibility Criteria for Healthy and CHR-P participants	Site sample (N) and mean age (SD) in years	Criteria for CHR-P status	Inclusion Criteria for Healthy Participants	IQ Method
			neural measurements, incl. diabetes, epilepsy, multiple sclerosis, or traumatic brain injury with severity 7+ on TBI scale<br>• IQ >70				
**21**	Institute of Mental Health, Singapore and National University of Singapore, Singapore	Singapore	• 14-29 years<br>• No lifetime neurological disorder diagnosis<br>• Fluent in English<br>• No history of intellectual disability<br>• No lifetime history of illicit substance use	Healthy=52; 22.01 (4.21) CHR-P=99; 21.93 (3.59)	• Met CAARMS criteria<br>• No current antipsychotic or mood stabilizer use<br>• No medical cause associated with psychotic symptoms	• No lifetime psychiatric disorder diagnosis<br>• No first-degree relative with a psychotic disorder diagnosis	NA
**22**	Seoul National University Hospital, South Korea	SNUH	• 15-34 years<br>• Female or male<br>• No lifetime neurological disorder diagnosis or traumatic brain injury<br>• Fluent in Korean<br>• IQ >70<br>• No MRI contraindications	Healthy=72; 21.25 (2.51) CHR-P=71; 20.72 (3.81)	• Met SIPS criteria	• No lifetime psychiatric disorder diagnosis, including psychotic disorder diagnosis<br>• No current psychotropic medication prescription<br>• No first- or second-degree relative with a psychotic disorder diagnosis<br>• No lifetime neurological disorder diagnosis	K-WAIS
**23**	Stavanger University Hospital, Norway	Stavanger	• 13-65 years<br>• Female, male<br>• No known neurological or endocrine disorders related to CHR symptoms<br>• Able to speak a	Healthy=33; 17.03 (3.10) CHR=P=35; 16.26 (1.88)	• Met SIPS criteria<br>• Symptoms not better accounted for by major Axis I, Axis II , or substance use disorder<br>• No current antipsychotic medication use<br>• Not more than 4 weeks of	• No lifetime psychiatric disorder diagnosis<br>• No lifetime psychotropic medication exposure<br>• No lifetime psychosis risk syndrome (SIPS criteria)<br>• No first-degree relatives with a psychotic disorder	WAIS-IV

© 2023 American Medical Association. All rights reserved.

#	Full Site Name	Abbreviated Site Name	Major Eligibility Criteria for Healthy and CHR-P participants	Site sample (N) and mean age (SD) in years	Criteria for CHR-P status	Inclusion Criteria for Healthy Participants	IQ Method
			Scandinavian language<br>• IQ >70<br>• No MRI contraindications		lifetime antipsychotic medication use	diagnosis<br>• No current substance use disorder	
**24**	Department of Neuro-psychiatry, Toho University School of Medicine, Japan	Toho	• 16-35 years<br>• No lifetime neurological disorder diagnosis<br>• Fluent in Japanese<br>• IQ >70<br>• No MRI contraindications	Healthy=15; 22.87 (2.64)<br>CHR=P=34; 23.71 (6.9)	• Met SIPS criteria	• No lifetime psychiatric disorder diagnosis<br>• No psychotropic medication exposure	WAIS-III
**25**	Department of Neuropsychiatry Graduate School of Medicine, The University of Tokyo	Tokyo	• 15-30 years old<br>• No neurological disorder diagnosis or traumatic brain injury with known cognitive consequences and/or loss of consciousness >5 mins<br>• Fluent in Japanese<br>• IQ ≥70<br>• No substance use disorder or substance abuse<br>• No history of electroconvulsive therapy	Healthy=25; 22.08 (2.84)<br>CHR-P=38; 20.92 (3.55)	• Met SIPS criteria	• No psychiatric condition<br>• No psychotropic medication use<br>• No first-degree relatives with a psychotic disorder diagnosis	JART25

© 2023 American Medical Association. All rights reserved.

#	Full Site Name	Abbreviated Site Name	Major Eligibility Criteria for Healthy and CHR-P participants	Site sample (N) and mean age (SD) in years	Criteria for CHR-P status	Inclusion Criteria for Healthy Participants	IQ Method
**26**	Centre for Addiction and Mental Health, University of Toronto, Canada	Toronto	• 18-40 years<br>• No current neurological disorder diagnosis<br>• Fluent in English or French<br>• IQ >70 (via clinical impression)<br>• Not currently pregnant<br>• No current drug or alcohol abuse<br>• No lifetime history of severe head trauma	Healthy=36; 25.37 (5.1)<br>CHR-P=25; 20.84 (1.9)	• Met SIPS criteria	• No lifetime psychiatric disorder diagnosis<br>• No psychotropic medication prescription<br>• No relatives with a psychiatric disorder diagnosis	RBANS
**27**	Graduate School of Medicine and Pharmaceutical Sciences, University of Toyama, Japan	Toyama	• 12-42 years<br>• No lifetime neurological disorder diagnosis<br>• Fluent in Japanese<br>• No MRI contraindications	Healthy =139; 25.04 (4.24)<br>CHR-P=73; 18.59 (4.11)	• Met CAARMS criteria	• No lifetime psychiatric disorder diagnosis<br>• No lifetime psychotropic medication exposure<br>• No first-degree relative with a psychiatric disorder diagnosis<br>• Physically healthy at time of MRI scanning<br>• No history of severe obstetric complications, serious head trauma, serious medical disease (e.g. neurological illness, thyroid dysfunction, diabetes, and hypertension), steroid use, or substance abuse.	JART50

© 2023 American Medical Association. All rights reserved.

#	Full Site Name	Abbreviated Site Name	Major Eligibility Criteria for Healthy and CHR-P participants	Site sample (N) and mean age (SD) in years	Criteria for CHR-P status	Inclusion Criteria for Healthy Participants	IQ Method
**28**	University of California, San Francisco, USA	UCSF	• 11-30 years<br>• No lifetime neurological disorder diagnosis<br>• Fluent in English<br>• No MRI contraindications<br>• No substance dependence (past 12 months, excluding nicotine)<br>• No head injury<br>• Good physical health	Healthy=100<br>;<br>23.9 (7.6)<br>CHR-P=65;<br>19.55 (4.46)	• Met SIPS criteria	• No lifetime DSM-IV Axis I disorder diagnosis<br>• No first-degree relatives with a psychotic disorder diagnosis	WASI-II
**29**	Psychiatric Hospital, University of Zurich, Switzerland	Zurich	• 13-35 years<br>• No neurological disorder diagnosis, no organic brain abnormalities (confirmed by neuroradiologist blinded to group allocation)<br>• Fluent in German<br>• IQ ≥80<br>• No pregnancy<br>• No drug or alcohol dependence	Healthy=43;<br>22.23 (5.56)<br>CHR-P=57;<br>19.37 (4.97)	• Met SIPS criteria<br>• No other psychiatric disorder diagnosis	• No lifetime psychiatric diagnosis<br>• No psychotropic medication use<br>• No first-degree relatives with a psychotic disorder diagnosis	MWT-B<br>WISC

© 2023 American Medical Association. All rights reserved.

eTable 2. SIPS and the CAARMS Items and Clinical High-Risk Criteria

	**SIPS**	**CAARMS**
Subscales	P1: Unusual Thought Content/Delusional Ideas	P1: Unusual Thought Content
	P2: Suspiciousness/Persecutory Ideas	P2: Non-Bizarre Ideas
	P3: Grandiose Ideas	
	P4: Perceptual Abnormalities/Hallucinations	P3: Perceptual Abnormalities
	P5: Disorganized Communication	P4: Disorganized Speech
Frequency	1: at least several minutes per day at least 1/month<br>2: several minutes/day at least once/week in the past month<br>3: at least 1 hour/day for at least 4 days/week over 1 month	0: absent<br>1: less than 1/month<br>2: 1/month to 2/weeks, <1 hour per occasion<br>3: 1/month to 2/weeks, >1 hour per occasion, OR 3 to 6/weeks <1 h per occasion<br>4: 3 to 6/week, > 1 hour per occasion, OR daily, <1 hour per occasion<br>5: daily, >1 hour per occasion, OR several times/ day<br>6: continuous
Substance use	Exclusion criterion if strongly intertwined with symptoms	0: no relation to substance use noted<br>1: occurs in relation to substance use and at other times as well<br>2: noted only in relation to substance use
Distress	Subjective qualifier<br>Not used to determine an individual's status	Rated on a scale of 0–100<br>Not used to determine an individual's status
**Attenuated psychotic symptoms**		
Inclusion criteria	Severity score of 3–5 on at least one of P1–P5<br>PLUS<br>Frequency score of 2 on P1, P2, P3, P4, and/or P5	Subthreshold intensity<br>Severity score of 3–5 on P1, 3–5 on P2, 3-4 on P3, and/or 4-5 on P4<br>PLUS<br>Frequency score of 3–6 on P1, P2, P3, and/or P4<br>Subthreshold frequency<br>Severity score of 6 on at least one of P1, P2, and P4 and/or 5-6 on P3<br>PLUS<br>Frequency score of 3 on P1, P2, P3, and/or P4
Onset	Symptoms should have begun within the past year OR currently rate one or more scale points higher compared to 12 months before<br>Symptoms that occurred over the past month only are rated	Symptoms should have been present in the previous 12 months and for not longer than 5 years
Level of functioning	No social/occupational dysfunction requirement	30% drop in SOFAS score from premorbid level, sustained for a month, within the past 12 months OR SOFAS score <50 for the past 12 months or more
Exclusion criteria	Symptoms are strongly intertwined temporally with substance use episodes (substance-induced psychosis may be considered)<br>Symptoms are better accounted for by another DSM diagnosis	Symptoms occur only during peak intoxication from a substance known to be associated with psychotic experiences (e.g., hallucinogens, amphetamines, and cocaine)<br>—<br>The person has had a previous psychotic episode (treated or untreated)

© 2023 American Medical Association. All rights reserved.

	SIPS	CAARMS
	Past psychosis was ruled in according to information obtained through the initial screen and evaluated using the POPS	
**Brief intermittent psychotic symptoms**		
Inclusion criteria	Severity score of 6 on at least one of P1–P5<br>PLUS<br>Frequency score of 1 on P1, P2, P3, P4, and/or P5	Severity score of 6 on at least one of P1, P2, and P4 and/or 5-6 on P3<br>PLUS<br>Frequency score of 4–6 on P1, P2, P3, and/or P4
Onset	Symptoms should have reached a psychotic level of intensity in the previous 3 month	Symptoms should have been present in the previous 12 months and for not longer than 5 years
Duration	Up to 3 months	Up to 7 days
Level of functioning	No social/occupational dysfunction requirement	30% drop in SOFAS score from premorbid level, sustained for a month, within the past 12 months<br>OR<br>SOFAS score <50 for the past 12 months or more
Exclusion criteria	Symptoms are strongly intertwined temporally with substance use episodes (substance-induced psychosis may be considered)<br>Symptoms are better accounted for by another DSM diagnosis<br>Past psychosis was ruled in according to information obtained through the initial screen and evaluated using the POPS<br>Symptoms are seriously disorganizing and dangerous	Symptoms occur only during peak intoxication from a substance known to be associated with psychotic experiences (e.g., hallucinogens, amphetamines, and cocaine)<br>—<br>Previous psychotic episode (treated or untreated)<br>—<br>Symptoms do not resolve spontaneously (without antipsychotic medication)
**Genetic risk and deterioration syndrome**		
Inclusion criteria	Meets criteria for Schizotypal Personality Disorder<br>OR<br>Has a first-degree relative with a psychotic disorder	Schizotypal Personality Disorder in identified patient<br>OR<br>Family history of psychosis in a first-degree relative
Level of functioning	30% drop in GAF score over the last month as compared to 12 months before	30% drop in SOFAS score from premorbid level, sustained for a month, within the past 12 months OR SOFAS score <50 for the past 12 months or more

CAARMS=Comprehensive Assessment of At-Risk Mental States; d=day; GAF=Global Assessment of Functioning; SIPS=Structured Interview for Psychosis-Risk Syndrome; SOFAS=Social and Occupational Functioning Assessment Scale

© 2023 American Medical Association. All rights reserved.

eTable 3. Neuroimaging Acquisition Protocols and FreeSurfer Version per Site

Site	Scanner Vendor	Scanner Model	Magnet Strength (Tesla)	Repetition Time (ms)	Echo Time (ms)	Flip Angle	Voxel Size (mm)	FreeSurfer version
Amsterdam	Philips	Image MR Series	3	8280	3.8	•	•	6.0.0
Columbia1	GE	Discovery MR750	3	7840	3.1	12°	0.8x0.8x0.8	6.0.0
Columbia3	GE	Signa	3	•	•	11°	0.98x0.98x1.0	5.3
Copenhagen	Philips	Achieva	3	10.028	4.6	8°	0.75x0.75x0.80	6.0.0
		Achieva	3	10.01	4.6	8°	0.75x0.75x0.80	6.0.0
CSU	Siemens	Skyra	3	2530	2.33	7°	1.0x1.0x1.0	6.0.0
Fetz Bern	Siemens	Magnetom Verio	3	7920	2.48	16°	1.0x1.0x1.0	6.0.0
Glasgow	Siemens	Trio	3	2250	2.6	9°	1.0x1.0x1.0	6.0.0
Heidelberg	Siemens	PET/MR	3	2300	2.98	9°	1.0x1.0x1.0	6.0.0
		Tim Trio	3	2300	2.98	9°	1.0x1.0x1.0	6.0.0
IDIBAPS	Siemens	Tim Trio	3	2300	3.01	9°	0.94x0.94x1.0	6.0.0
		Prisma fit	3	2300	3.01	9°	0.94x0.94x1.0	6.0.0
		Prisma fit	3	2300	2.98	9°	1.0x1.0x1.2	6.0.0
London	GE	Signa HDx	3	7.144	2.85	20°	1.1x1.1.x1.1	6.0.0
Maastricht	Philips	Intera	3	2250	4.6	8°	1.17×1.17×1.20	6.0.0
		Achieva	3	2250	4.6	8°	1.2x0.8x0.8	6.0.0
Melbourne	Siemens	Trio	3	1900	2.15	90°	0.5x0.5x1.0	6.0.0
		Trio	3	2300	2.98	9°	1.0x1.0x1.2	6.0.0
	GE	Signa	1.5	14.3	3.3	30°	0.94x0.94x1.5	6.0.0

© 2023 American Medical Association. All rights reserved.

Site	Scanner Vendor	Scanner Model	Magnet Strength (Tesla)	Repetition Time (ms)	Echo Time (ms)	Flip Angle	Voxel Size (mm)	FreeSurfer version
		LX Horizon	3	36	9	30°	0.49x0.49x2	6.0.0
Mexico City	GE	Signa HDxt	3	1340	5.7	20°	1.2x1.2x1.2	6.0.0
MHRC	Philips	Achieva	3	8.2	3.7	8°	0.83×0.83x1.0	6.0.0
MPRC	Siemens	Trio	3	2400	2.2	8°	0.8x0.8x0.8	6.0.0
		Prisma	3	2400	2.2	8°	0.8x0.8x0.8	6.0.0
ISMMS	Siemens	Skyra	3	2400	2.07	8°	0.8x0.8x0.8	6.0.0
Newcastle	Siemens	Avanto	1.5	1980	4.3	15°	0.98x0.98x1	6.0.0
Oslo Region	GE	Signa HDxt	3	7800	2.96	12°	1.0×1.0×1.2	5.3
		Discovery MR750	3	8.16	3.18	12°	1.0x1.0x1.0	5.3
Pitt	Siemens	Prisma	3	2400	2.2	8°	0.8x0.8x0.8	6.0.0
RUMC	Siemens	Verio	3	2530	2.27	7°	1.0x1.0x1.0	6.0.0
Singapore	Siemens	Tim Trio	3	2300	3	9°	1.0x1.0x1.0	6.0.0
SNUH	Siemens	Magnetom TrioTim	3	1670	1.89	9°	1x0.98x0.98	6.0.0
Stavanger	GE	Discovery 450	1.5	7.9	3.1	12°	•	5.3
Toho	Toshiba	Excelart Vantage	1.5	•	•	35°	0.98×0.98×1.0	5.2
Tokyo	GE	SIGNA HDx	3	6.8	1.94	20°	1.0x1.0x1.0	6.0.0
		Discovery MR750W	3	8.46	3.25	20°	1.0x1.0x1.0	6.0.0
Toronto	GE	Discovery MR750	3	6736	2.99	8°	0.9x0.9x0.9	6.0.0
Toyama	Siemens	Magnetom Vision	1.5	2400	5	40°	1.0x1.0x1.0	6.0.0

© 2023 American Medical Association. All rights reserved.

Site	Scanner Vendor	Scanner Model	Magnet Strength (Tesla)	Repetition Time (ms)	Echo Time (ms)	Flip Angle	Voxel Size (mm)	FreeSurfer version
		Magnetom Verio	3	2300	2.9	9°	1.0x1.0x1.2	6.0.0
UCSF	Siemens	Magnetom TrioTim	3	2300	2.95	9°	1.0x1.0x1.2	5.1
Zurich	Philips	Achieva TX	3	8.3	3.8	8°	1.0x1.0x1.0	6.0.0

Unavailable information left blank

© 2023 American Medical Association. All rights reserved.

eTable 4. Demographic Characteristics of Study Participants per Site

#	Site	Healthy Individuals (N = 1237)			Clinical High-Risk for Psychosis (N = 1340)		
		N	Age (years) Mean (SD)	Sex # Male (%)	N	Age (years) Mean (SD)	Sex # Male (%)
**1**	Amsterdam	23	23.45 (2.80)	10 (43.5 %)	16	23.64 (2.51)	6 (37.5 %)
**2**	Columbia1	9	24.35 (4.05)	7 (77.8 %)	17	22.89 (4.96)	8 (47.1 %)
**3**	Columbia3	35	22.91 (3.68)	23 (65.7 %)	53	21.27 (4.01)	39 (73.6 %)
**4**	Copenhagen	58	24.78 (3.30)	29 (50.0 %)	158	24.21 (4.22)	72 (45.6 %)
**5**	CSU	55	21.47 (3.20)	30 (54.5 %)	49	19.49 (5.05)	26 (53.1 %)
**6**	Bern	15	20.11 (6.02)	10 (66.7 %)	39	19.14 (4.81)	20 (51.3 %)
**7**	Glasgow	45	22.89 (3.61)	15 (33.3 %)	75	22.45 (4.82)	15 (20.0 %)
**8**	Heidelberg	31	15.71 (0.90)	15 (48.4 %)	22	15.14 (1.08)	9 (40.9 %)
**9**	IDIBAPS	44	16.00 (1.53)	14 (31.8 %)	60	15.32 (1.80)	20 (33.3 %)
**10**	ISMMS	11	27.89 (3.95)	6 (54.5 %)	24	23.43 (5.55)	11 (45.8 %)
**11**	London_1	12	26.42 (4.78)	7 (58.3 %)	46	23.52 (4.64)	35 (76.1 %)
**12**	Maastricht	37	25.52 (5.73)	26 (70.3 %)	39	20.13 (4.18)	27 (69.2 %)
**13**	Melbourne	90	21.76 (3.59)	48 (53.3 %)	18	18.39 (2.87)	7 (38.9 %)
**14**	Mexico City	37	20.97 (3.40)	28 (75.7 %)	30	19.70 (4.22)	24 (80.0 %)
**15**	MHRC	33	22.52 (2.52)	33 (100.0 %)	18	20.03 (2.61)	18 (100.0 %)
**16**	MPRC	19	18.00 (4.28)	11 (57.9 %)	29	17.28 (3.21)	14 (48.3 %)
**17**	Newcastle	17	20.25 (1.91)	4 (23.5 %)	43	19.53 (2.12)	20 (46.5 %)
**18**	Oslo Region	62	19.90 (3.62)	39 (62.9 %)	20	20.08 (3.61)	12 (60.0 %)
**19**	Pitt	60	22.93 (5.53)	35 (58.3 %)	25	20.87 (5.40)	11 (44.0 %)
**20**	RUMC	29	23.88 (3.22)	10 (34.5 %)	62	22.74 (3.93)	33 (53.2 %)
**21**	Singapore	52	22.01 (4.21)	27 (51.9 %)	99	21.93 (3.59)	68 (68.7 %)
**22**	SNUH	72	21.25 (2.51)	49 (68.1 %)	71	20.72 (3.81)	52 (73.2 %)
**23**	Stavanger	33	17.03 (3.10)	17 (51.5 %)	35	16.26 (1.88)	13 (37.1 %)
**24**	Toho	15	22.87 (2.64)	8 (53.3 %)	34	23.71 (6.90)	7 (20.6 %)
**25**	Tokyo	25	22.08 (2.84)	13 (52.0 %)	38	20.92 (3.55)	20 (52.6 %)
**26**	Toronto	36	25.37 (5.10)	20 (55.6 %)	25	20.84 (1.90)	14 (56.0 %)
**27**	Toyama	139	25.04 (4.24)	72 (51.8 %)	73	18.59 (4.11)	40 (54.8 %)
**28**	UCSF	100	23.90 (7.60)	57 (57.0 %)	65	19.55 (4.46)	35 (53.8 %)
**29**	Zurich	43	22.23 (5.56)	21 (48.8 %)	57	19.37 (4.97)	33 (57.9 %)

© 2023 American Medical Association. All rights reserved.

eTable 5. Characteristics of Clinical High-Risk for Psychosis (CHR-P) Individuals Defined With Either the SIPS or CAARMS Criteria

	**CHR-P individuals meeting SIPS criteria**		**CHR-P individuals meeting CAARMS criteria**	
	**N**		**N**	
**Age in years, mean (SD)**	806	19.90 (4.69)	534	22.03 (4.52)
**Sex, number male (%)**	806	444 (55.09%)	534	265 (49.63%)
**Positive symptoms score, mean (SD)**	806	10.93 (4.66)	534	10.37 (4.03)
**Prescribed typical antipsychotics, number (%)**	744	21 (2.82%)	526	1 (0.19%)
**Prescribed atypical antipsychotics, number (%)**	748	169 (22.59%)	526	62 (11.79)
**Converters, N (%)**	641	115 (17.94%)	456	42 (9.21%)
**Follow-up period in months, mean (SD)[a]**	597	19.07 (10.67)	392	20.60 (17.54)

[a]The duration of follow-up was unavailable in 108 CHR-P individuals.

© 2023 American Medical Association. All rights reserved.

eTable 6. FreeSurfer-Derived Morphometric Measures

Measure	Side	Regions
**Cortical Thickness**	**L**	left_bankssts, left_caudalanteriorcingulate, left_caudalmiddlefrontal, left_cuneus, left_entorhinal, left_fusiform, left_inferiorparietal, left_inferiortemporal, left_isthmuscingulate, left_lateraloccipital, left_lateralorbitofrontal, left_lingual, left_medialorbitofrontal, left_middletemporal, left_parahippocampal, left_paracentral, left_parsopercularis, left_parsorbitalis, left_parstriangularis, left_pericalcarine, left_postcentral, left_posteriorcingulate, left_precentral, left_precuneus, left_rostralanteriorcingulate, left_rostralmiddlefrontal, left_superiorfrontal, left_superiorparietal, left_superiortemporal, left_supramarginal, left_frontalpole, left_temporalpole, left_transversetemporal, left_insula
	**R**	right_bankssts, right_caudalanteriorcingulate, right_caudalmiddlefrontal, right_cuneus, right_entorhinal, right_fusiform, right_inferiorparietal, right_inferiortemporal, right_isthmuscingulate, right_lateraloccipital, right_lateralorbitofrontal, right_lingual, right_medialorbitofrontal, right_middletemporal, right_parahippocampal, right_paracentral, right_parsopercularis, right_parsorbitalis, right_parstriangularis, right_pericalcarine, right_postcentral, right_posteriorcingulate, right_precentral, right_precuneus, right_rostralanteriorcingulate, right_rostralmiddlefrontal, right_superiorfrontal, right_superiorparietal, right_superiortemporal, right_supramarginal, right_frontalpole, right_temporalpole, right_transversetemporal, right_insula
**Surface Area**	**L**	left_bankssts, left_caudalanteriorcingulate, left_caudalmiddlefrontal, left_cuneus, left_entorhinal, left_fusiform, left_inferiorparietal, left_inferiortemporal, left_isthmuscingulate, left_lateraloccipital, left_lateralorbitofrontal, left_lingual, left_medialorbitofrontal, left_middletemporal, left_parahippocampal, left_paracentral, left_parsopercularis, left_parsorbitalis, left_parstriangularis, left_pericalcarine, left_postcentral, left_posteriorcingulate, left_precentral, left_precuneus, left_rostralanteriorcingulate, left_rostralmiddlefrontal, left_superiorfrontal, left_superiorparietal, left_superiortemporal, left_supramarginal, left_frontalpole, left_temporalpole, left_transversetemporal, left_insula
	**R**	right_bankssts, right_caudalanteriorcingulate, right_caudalmiddlefrontal, right_cuneus, right_entorhinal, right_fusiform, right_inferiorparietal, right_inferiortemporal, right_isthmuscingulate, right_lateraloccipital, right_lateralorbitofrontal, right_lingual, right_medialorbitofrontal, right_middletemporal, right_parahippocampal, right_paracentral, right_parsopercularis, right_parsorbitalis, right_parstriangularis, right_pericalcarine, right_postcentral, right_posteriorcingulate, right_precentral, right_precuneus, right_rostralanteriorcingulate, right_rostralmiddlefrontal, right_superiorfrontal, right_superiorparietal, right_superiortemporal, right_supramarginal, right_frontalpole, right_temporalpole, right_transversetemporal, right_insula
**Subcortical Volumes**	**L**	Left Thalamus, Left Caudate, Left Putamen, Left Pallidum, Left Hippocampus, Left Amygdala, Left Accumbens area
	**R**	Right Thalamus, Right Caudate, Right Putamen, Right Pallidum, Right Hippocampus, Right Amygdala, Right Accumbens area

© 2023 American Medical Association. All rights reserved.

eTable 7. Percentage of Individuals With Infra- or Supranormal Normative Regional *z* Scores Based on Group

		Normative z-scores in HI		Normative z-scores in CHR-P	
**Region**	**Hemi**	**infranormal z-scores (%)**	**supranormal z-scores (%)**	**infranormal z-scores (%)**	**supranormal z-scores (%)**
**Subcortical Volume**					
nucleus accumbens	L	4.85	0.49	5.82	0.52
	R	5.17	0.40	7.01	0.07
amygdala	L	3.31	0.40	3.73	0.60
	R	2.67	1.37	3.73	2.01
caudate	L	3.31	0.89	5.97	0.60
	R	2.75	0.81	4.93	1.12
hippocampus	L	3.07	0.24	4.78	0.75
	R	3.07	0.65	3.88	0.67
pallidum	L	3.96	0.40	5.67	0.37
	R	4.20	0.81	6.04	0.97
putamen	L	8.57	0.40	10.00	0.37
	R	9.30	0.24	11.42	0.22
thalamus	L	6.95	0.08	8.28	0.82
	R	6.22	0.57	8.58	0.30
**Cortical Thickness**					
banks superior temporal sulcus	L	2.26	2.34	2.31	1.57
	R	2.75	2.18	3.43	2.24
caudal anterior cingulate	L	2.67	4.45	2.54	3.13
	R	3.15	4.37	3.66	4.40
caudal middle frontal	L	2.83	1.37	2.46	1.12
	R	2.83	1.54	2.84	1.64
cuneus	L	1.37	1.94	1.87	2.31
	R	0.81	2.51	1.87	3.06
entorhinal cortex	L	1.46	3.31	2.46	4.18
	R	2.02	2.67	2.69	3.66
fusiform gyrus	L	1.78	2.43	1.42	2.99
	R	1.94	2.43	1.87	3.13
inferior parietal	L	1.21	2.34	1.57	2.61
	R	1.94	1.21	2.09	1.94
inferior temporal	L	1.13	2.75	1.27	3.28
	R	0.89	2.51	1.94	4.85
isthmus cingulate	L	4.20	1.37	5.52	1.64
	R	4.61	2.43	4.85	1.49
lateral occipital	L	1.21	5.50	0.60	4.78
	R	0.57	4.45	0.90	4.85
lateral orbitofrontal	L	2.91	1.94	1.64	1.79
	R	1.37	2.59	1.64	2.31
lingual gyrus	L	2.99	2.59	3.51	2.09

© 2023 American Medical Association. All rights reserved.

Region	Hemi	Normative z-scores in HI		Normative z-scores in CHR-P	
		infranormal z-scores (%)	supranormal z-scores (%)	infranormal z-scores (%)	supranormal z-scores (%)
	R	2.18	2.34	2.16	2.24
medial orbitofrontal	L	1.62	2.34	2.09	3.28
	R	1.46	2.67	1.34	3.13
middle temporal	L	1.54	0.97	2.24	1.79
	R	1.29	1.21	2.01	1.57
parahippocampal	L	1.62	1.94	2.31	1.87
	R	2.43	2.75	2.16	2.84
paracentral	L	0.97	1.54	1.57	1.94
	R	1.46	1.21	1.94	1.12
pars opercularis	L	4.77	0.81	3.81	1.19
	R	3.80	0.97	3.81	0.97
pars orbitalis	L	2.75	2.26	2.61	1.79
	R	2.18	1.54	1.64	2.76
pars triangularis	L	3.80	0.65	3.13	1.87
	R	3.88	0.81	3.28	1.12
pericalcarine	L	3.48	3.64	4.25	3.58
	R	3.23	3.07	2.99	5.15
postcentral	L	1.13	1.94	0.82	2.31
	R	0.49	1.13	1.12	1.57
posterior cingulate	L	5.01	1.70	5.67	2.01
	R	3.31	1.78	4.18	1.49
precentral	L	2.34	0.81	2.84	0.90
	R	2.02	0.32	2.76	0.52
precuneus	L	2.18	1.21	1.42	2.09
	R	1.46	0.81	2.31	1.72
rostral anterior cingulate	L	2.26	1.70	3.43	2.54
	R	1.86	1.86	2.91	2.54
rostral middle frontal	L	0.81	1.86	1.64	2.01
	R	1.05	2.67	1.27	2.39
superior frontal	L	4.53	0.89	3.36	1.57
	R	3.40	1.21	2.76	1.64
superior parietal	L	0.57	2.51	0.52	2.91
	R	0.57	1.62	0.82	2.61
superior temporal	L	3.15	0.49	3.13	0.60
	R	2.34	1.29	3.36	0.90
supramarginal gyrus	L	2.99	1.21	2.39	1.04
	R	2.43	1.13	4.10	1.49
frontal pole	L	2.83	2.99	2.46	3.88
	R	2.43	3.56	2.31	3.66
temporal pole	L	2.02	2.34	1.27	2.54
	R	2.26	1.29	3.06	1.94

© 2023 American Medical Association. All rights reserved.

		Normative z-scores in HI		Normative z-scores in CHR-P	
**Region**	**Hemi**	**infranormal z-scores (%)**	**supranormal z-scores (%)**	**infranormal z-scores (%)**	**supranormal z-scores (%)**
transverse temporal	L	2.83	1.37	2.61	1.57
	R	2.43	1.13	1.64	1.87
insula	L	2.99	1.21	4.48	0.60
	R	3.48	0.57	4.48	0.37
**Surface Area**					
banks superior temporal sulcus	L	1.54	2.75	2.39	1.79
	R	2.99	2.75	2.01	2.24
caudal anterior cingulate	L	0.65	4.28	0.30	3.21
	R	0.89	2.34	0.60	3.13
caudal middle frontal	L	1.46	2.26	1.64	2.39
	R	0.89	2.26	1.49	2.99
cuneus	L	1.54	2.75	2.46	2.69
	R	1.70	2.59	1.72	2.91
entorhinal cortex	L	2.51	6.95	2.61	5.52
	R	1.37	5.01	1.34	5.07
fusiform gyrus	L	1.70	2.02	2.91	2.24
	R	2.91	1.70	2.61	1.87
inferior parietal	L	2.02	1.94	3.13	1.87
	R	2.43	2.10	3.06	2.84
inferior temporal	L	3.64	2.59	2.31	2.76
	R	2.99	2.59	2.46	2.16
isthmus cingulate	L	1.05	3.23	0.75	3.06
	R	1.29	3.23	0.82	3.81
lateral occipital	L	1.94	2.99	2.09	3.06
	R	1.46	2.43	1.64	2.61
lateral orbitofrontal	L	2.67	1.21	2.46	2.24
	R	2.51	2.26	2.84	3.28
lingual gyrus	L	2.51	2.51	2.99	2.01
	R	2.75	2.59	2.84	3.06
medial orbitofrontal	L	1.70	3.96	1.79	3.21
	R	1.94	2.99	2.46	2.69
middle temporal	L	3.88	1.62	3.06	3.21
	R	2.91	2.18	2.54	2.69
parahippocampal	L	1.46	2.59	2.09	2.16
	R	2.67	2.75	3.58	3.13
paracentral	L	1.13	2.34	0.82	2.91
	R	1.54	2.83	0.82	3.06
pars opercularis	L	1.54	3.72	1.94	2.39
	R	1.05	3.80	0.97	3.28
pars orbitalis	L	2.51	1.70	2.39	2.84
	R	2.67	1.62	1.72	2.24

© 2023 American Medical Association. All rights reserved.

		Normative z-scores in HI		Normative z-scores in CHR-P	
**Region**	**Hemi**	**infranormal z-scores (%)**	**supranormal z-scores (%)**	**infranormal z-scores (%)**	**supranormal z-scores (%)**
pars triangularis	L	2.83	2.18	2.84	1.49
	R	2.91	1.70	1.72	1.94
pericalcarine	L	1.86	2.99	1.49	2.99
	R	2.26	2.51	2.24	2.01
postcentral	L	0.97	4.12	0.90	5.45
	R	1.21	4.28	1.79	3.43
posterior cingulate	L	1.62	2.51	1.42	2.91
	R	1.54	2.34	1.49	2.46
precentral	L	2.10	3.88	1.27	4.70
	R	1.21	3.23	0.45	3.96
precuneus	L	1.37	2.02	2.24	2.91
	R	1.54	1.21	1.94	2.91
rostral anterior cingulate	L	0.97	3.88	1.34	3.28
	R	0.73	2.02	0.60	2.24
rostral middle frontal	L	2.18	1.86	2.69	2.61
	R	1.54	2.10	2.31	3.13
superior frontal	L	2.99	3.07	3.66	3.28
	R	2.26	2.67	2.69	2.31
superior parietal	L	1.86	2.10	1.64	2.61
	R	1.94	1.62	2.24	3.06
superior temporal	L	2.10	2.91	2.54	3.43
	R	2.02	3.07	1.79	2.99
supramarginal gyrus	L	2.43	5.09	2.61	3.81
	R	1.62	2.26	1.79	1.87
frontal pole	L	1.29	1.78	1.79	1.12
	R	0.65	1.21	1.12	1.42
temporal pole	L	1.05	1.29	1.12	1.94
	R	1.29	2.99	0.90	2.99
transverse temporal	L	0.81	2.83	1.19	3.36
	R	0.73	1.94	0.75	2.31
insula	L	0.81	6.22	1.34	6.72
	R	1.54	6.31	1.12	7.01

[a] significant two-proportion z-tests difference in infranormal z-scores between CHR-P and HI at $P_{FDR} < 0.05$; [b] significant two-proportion z-tests difference in supranormal z-scores between CHR-P and HI at $P_{FDR} < 0.05$; CHR-P = clinical high-risk for psychosis; Hemi = hemisphere; HI = healthy individuals; L = left; R = right.

© 2023 American Medical Association. All rights reserved.

eTable 8. Percentage of CHR-P With Infra- or Supranormal Normative Regional *z* Scores According to Medication Status

Region	Hemi	Unmediated CHR-P Individuals (N = 1061)		Mediated CHR-P Individuals (N = 243)	
		infranormal z-scores (%)	supranormal z-scores (%)	infranormal z-scores (%)	supranormal z-scores (%)
**Subcortical Volume**					
nucleus accumbens	L	5.00	0.57	8.64	0.00
	R	6.03	0.00	11.11	0.41
amygdala	L	3.49	0.57	4.53	0.82
	R	3.30	2.17	5.76	1.65
caudate	L	5.56	0.57	7.82	0.82
	R	4.43	0.94	7.41	2.06
hippocampus	L	4.05	0.75	7.82	0.82
	R	3.49	0.66	5.76	0.82
pallidum	L	5.00	0.28	8.64	0.82
	R	5.18	1.13	9.47	0.41
putamen	L	9.61	0.47	11.93	0.00
	R	11.50	0.28	11.93	0.00
thalamus	L	7.35	0.66	11.52	1.65
	R	7.35	0.38	14.40	0.00
**Cortical Thickness**					
banks superior temporal sulcus	L	2.26	1.60	2.06	1.23
	R	3.49	2.07	2.88	2.88
caudal anterior cingulate	L	2.64	3.02	2.47	4.12
	R	3.86	4.52	2.88	4.53
caudal middle frontal	L	2.64	1.04	1.65	1.65
	R	2.83	1.79	2.47	1.23
cuneus	L	1.79	2.73	2.47	0.82
	R	1.98	2.92	1.65	3.70
entorhinal cortex	L	2.73	4.71	1.23	2.06
	R	2.73	4.05	2.88	2.47
fusiform gyrus	L	1.51	2.83	1.23	4.12
	R	1.98	2.73	1.65	4.94
inferior parietal	L	1.23	2.92	3.29	1.23
	R	1.89	2.17	2.88	0.82
inferior temporal	L	1.23	3.20	1.65	3.70
	R	1.70	5.66	3.29	1.65
isthmus cingulate	L	5.28	1.51	4.53	2.47
	R	4.62	1.51	5.35	1.23
lateral occipital	L	0.66	4.62	0.41	4.53
	R	0.85	4.52	1.23	5.76
lateral orbitofrontal	L	1.89	1.79	0.82	2.06
	R	1.79	2.17	1.23	2.06

© 2023 American Medical Association. All rights reserved.

		Unmediated CHR-P Individuals (N = 1061)		Mediated CHR-P Individuals (N = 243)	
Region	Hemi	infranormal z-scores (%)	supranormal z-scores (%)	infranormal z-scores (%)	supranormal z-scores (%)
lingual gyrus	L	3.02	1.89	5.76	3.29
	R	2.17	2.17	1.65	2.88
medial orbitofrontal	L	1.98	3.30	2.47	3.29
	R	1.41	3.20	1.23	2.88
middle temporal	L	1.98	1.89	3.29	1.65
	R	1.79	1.70	2.88	1.23
parahippocampal	L	1.79	1.51	4.94	2.47
	R	1.98	2.92	3.29	2.88
paracentral	L	1.41	2.07	2.06	0.82
	R	1.89	1.04	1.65	0.41
pars opercularis	L	3.77	1.13	4.12	0.82
	R	3.86	1.13	4.12	0.41
pars orbitalis	L	2.36	1.89	3.70	0.82
	R	1.51	2.83	2.06	2.06
pars triangularis	L	3.30	1.98	2.47	1.23
	R	3.58	1.13	2.47	1.23
pericalcarine	L	4.34	3.68	3.70	3.29
	R	2.92	5.56	2.88	3.29
postcentral	L	0.94	2.64	0.41	1.23
	R	1.41	1.51	0.00	1.65
posterior cingulate	L	5.47	2.17	6.58	1.65
	R	4.24	1.51	3.29	1.65
precentral	L	2.83	0.85	2.88	0.82
	R	3.11	0.47	1.65	0.82
precuneus	L	1.32	1.98	1.65	1.65
	R	2.26	1.89	2.88	1.23
rostral anterior cingulate	L	3.49	2.26	3.29	3.70
	R	3.02	3.02	2.88	0.41
rostral middle frontal	L	1.70	1.89	1.23	2.06
	R	1.32	2.26	0.82	2.88
superior frontal	L	3.49	1.60	2.88	1.65
	R	3.30	1.60	0.82	1.65
superior parietal	L	0.57	2.83	0.41	2.88
	R	0.85	2.36	0.82	3.29
superior temporal	L	2.64	0.75	4.53	0.00
	R	3.30	1.04	3.29	0.41
supramarginal gyrus	L	2.45	0.85	2.47	2.06
	R	3.58	1.41	6.58	2.06
frontal pole	L	2.73	3.58	1.65	4.53
	R	2.73	3.77	0.82	3.70

© 2023 American Medical Association. All rights reserved.

		Unmediated CHR-P Individuals (N = 1061)		Mediated CHR-P Individuals (N = 243)	
**Region**	**Hemi**	**infranormal z-scores (%)**	**supranormal z-scores (%)**	**infranormal z-scores (%)**	**supranormal z-scores (%)**
temporal pole	L	1.23	2.54	1.65	2.47
	R	3.30	1.89	2.06	1.65
transverse temporal	L	2.73	1.70	2.06	0.82
	R	1.70	2.07	1.65	1.23
insula	L	4.62	0.47	3.70	0.82
	R	4.15	0.19	5.35	1.23
**Surface Area**					
banks superior temporal sulcus	L	2.73	1.60	0.82	2.88
	R	2.17	2.07	1.23	2.88
caudal anterior cingulate	L	0.19	3.02	0.41	4.53
	R	0.66	3.11	0.00	3.70
caudal middle frontal	L	1.51	2.36	2.47	2.47
	R	1.60	2.92	0.82	2.88
cuneus	L	2.54	2.83	2.47	1.65
	R	1.60	3.11	2.06	2.06
entorhinal cortex	L	2.92	5.47	1.23	6.17
	R	1.32	5.09	1.65	4.12
fusiform gyrus	L	2.83	2.36	3.29	2.06
	R	2.36	1.79	3.70	2.06
inferior parietal	L	3.30	1.89	2.88	2.06
	R	3.02	2.83	3.29	3.29
inferior temporal	L	2.17	2.45	2.88	3.70
	R	2.07	2.07	3.29	2.88
isthmus cingulate	L	0.75	2.54	0.82	5.76
	R	0.57	3.20	2.06	6.58
lateral occipital	L[b]	2.26	2.17	0.82	7.00
	R	1.23	2.26	3.29	2.88
lateral orbitofrontal	L	2.83	2.07	0.82	2.47
	R	2.73	3.11	3.29	3.70
lingual gyrus	L	2.92	1.98	3.70	2.06
	R	2.54	3.11	4.53	2.47
medial orbitofrontal	L	1.79	3.58	2.06	1.23
	R	2.36	2.26	3.29	4.53
middle temporal	L	3.20	2.83	2.47	4.53
	R	2.83	2.73	1.65	2.47
parahippocampal	L	2.07	1.89	2.06	3.29
	R	3.68	3.11	3.70	3.29
paracentral	L	0.85	2.73	0.82	3.70
	R	0.94	3.20	0.41	2.47
pars opercularis	L	1.70	2.07	3.29	3.70
	R	0.85	3.30	1.65	3.29

© 2023 American Medical Association. All rights reserved.

		Unmediated CHR-P Individuals (N = 1061)		Mediated CHR-P Individuals (N = 243)	
**Region**	**Hemi**	**infranormal z-scores (%)**	**supranormal z-scores (%)**	**infranormal z-scores (%)**	**supranormal z-scores (%)**
pars orbitalis	L	2.36	2.73	2.06	3.29
	R	1.60	2.54	2.06	1.23
pars triangularis	L	3.02	1.51	1.65	1.65
	R	1.70	2.07	1.65	1.23
pericalcarine	L	1.32	2.83	2.06	3.29
	R	2.26	2.07	1.65	2.06
postcentral	L	0.75	5.37	1.65	5.76
	R	1.98	3.30	1.23	3.70
posterior cingulate	L	1.41	2.92	1.65	2.47
	R	1.41	2.45	2.06	2.88
precentral	L	0.94	4.43	2.47	5.76
	R	0.47	3.96	0.41	4.12
precuneus	L	2.26	2.73	2.06	3.29
	R	2.07	2.73	1.65	2.88
rostral anterior cingulate	L	1.13	3.30	2.47	3.70
	R	0.75	2.54	0.00	1.23
rostral middle frontal	L	2.92	2.73	1.65	2.47
	R	2.26	2.73	2.47	4.94
superior frontal	L	3.96	3.49	2.47	2.06
	R	2.54	2.17	3.29	2.88
superior parietal	L	1.60	2.64	2.06	2.47
	R	1.98	3.02	3.29	2.88
superior temporal	L	2.36	3.49	3.29	3.70
	R	1.98	2.64	1.23	4.12
supramarginal gyrus	L	2.83	3.86	1.65	4.12
	R	1.60	2.17	2.88	0.41
frontal pole	L	1.70	1.04	2.06	1.65
	R	1.23	1.32	0.41	2.06
temporal pole	L	0.75	2.17	1.65	0.41
	R	0.94	3.11	0.82	2.88
transverse temporal	L	1.32	3.39	0.82	3.29
	R	0.75	2.73	0.82	0.41
insula	L	1.32	6.03	1.65	9.88
	R	0.94	6.88	2.06	7.82

[a] significant two-proportion z-tests difference in infranormal z-scores between CHR-P with and without antipsychotic medication exposure at $P_{FDR} < 0.05$; [b] significant two-proportion z-tests difference in supranormal z-scores between CHR-P with and without antipsychotic medication exposure at $P_{FDR} < 0.05$; CHR-P = clinical high-risk for psychosis; Hemi = hemisphere; L = left; R = right.

© 2023 American Medical Association. All rights reserved.

eTable 9. Effect Size (Cohen *d*) of Group Differences in Observed Brain Morphometric Data

Region	Hemi	Cohen's *d*<br><br>All CHR-P vs Healthy individuals	Cohen's *d*<br><br>CHR-PC vs Healthy individuals	Cohen's *d*<br><br>medicated CHR-P vs unmedicated CHR-P
**Subcortical Volumes**				
nucleus accumbens	L	-3.42E-03	-0.11	-0.20
	R	-0.01	-0.06	-0.14
amygdala	L	-0.03	-0.08	-0.17
	R	-0.07	-0.10	-0.15
caudate	L	0.01	-0.02	-0.06
	R	-0.02	-0.04	-0.07
hippocampus	L	-0.11	-0.21	-0.14
	R	-0.15	-0.26	-0.14
pallidum	L	-0.05	-0.13	-0.02
	R	-0.04	-0.20	-0.01
putamen	L	-0.02	-0.03	-0.06
	R	-0.05	-0.08	-0.01
thalamus	L	-0.03	-0.20	-0.05
	R	-0.09	-0.22	-0.14
**Cortical Thickness**				
banks superior temporal sulcus	L	-0.04	-0.09	0.01
	R	-0.03	-0.12	-0.05
caudal anterior cingulate	L	-0.03	0.02	-0.03
	R	-0.04	-0.04	0.08
caudal middle frontal	L	0.03	-0.06	-0.04
	R	-0.04	0.02	-0.02
cuneus	L	0.02	-0.03	-0.13
	R	-0.01	-0.10	-0.08
entorhinal cortex	L	0.02	-0.04	0.01
	R	0.01	-0.10	0.11
fusiform gyrus	L	-0.06	-0.15	0.06
	R	-0.02	-0.14	0.01

© 2023 American Medical Association. All rights reserved.

Region	Hemi	Cohen’s *d*<br><br>All CHR-P vs Healthy individuals	Cohen’s *d*<br><br>CHR-PC vs Healthy individuals	Cohen’s *d*<br><br>medicated CHR-P vs unmedicated CHR-P
inferior parietal	L	-0.05	-0.06	0.03
	R	0.04	0.12	-0.04
inferior temporal	L	0.01	0.03	0.03
	R	-0.01	-4.70E-03	-0.06
isthmus cingulate	L	-1.84E-03	-0.01	-0.05
	R	-3.40E-03	0.03	-1.90E-03
lateral occipital	L	-0.01	-0.11	-1.43E-03
	R	-0.04	-0.10	0.02
lateral orbitofrontal	L	-0.03	-0.03	-0.06
	R	4.36E-03	0.13	0.01
lingual gyrus	L	-0.05	-0.14	-0.02
	R	4.83E-03	-0.07	-0.05
medial orbitofrontal	L	-0.05	0.08	0.04
	R	3.58E-03	0.07	0.06
middle temporal	L	0.03	0.06	-0.01
	R	-0.01	-0.07	-0.02
parahippocampal	L	-0.07	-0.10	-0.07
	R	-0.03	-0.05	-0.10
paracentral	L	-0.05	-0.21	0.03
	R	-0.01	-0.17	-2.78E-03
pars opercularis	L	0.06	0.19	-0.05
	R	0.02	1.11E-03	0.03
pars orbitalis	L	0.02	0.04	-0.03
	R	0.10	0.04	-0.01
pars triangularis	L	0.10	0.09	-0.03
	R	0.05	0.03	0.05
pericalcarine	L	0.01	-0.04	-0.03
	R	0.02	0.01	0.03
postcentral	L	0.03	-0.01	-0.13
	R	-0.03	-0.06	-0.20
posterior cingulate	L	-0.04	0.02	-0.10

© 2023 American Medical Association. All rights reserved.

Region	Hemi	Cohen's *d*<br><br>All CHR-P vs Healthy individuals	Cohen's *d*<br><br>CHR-PC vs Healthy individuals	Cohen's *d*<br><br>medicated CHR-P vs unmedicated CHR-P
	R	-0.07	-0.10	0.06
precentral	L	0.03	0.10	0.02
	R	-3.97E-03	-0.05	0.03
precuneus	L	-8.14E-04	0.08	0.06
	R	-0.04	0.04	-0.13
rostral anterior cingulate	L	-0.02	-0.04	0.02
	R	-1.61E-03	-0.05	0.03
rostral middle frontal	L	-0.01	0.05	-0.24
	R	1.62E-03	0.03	-0.09
superior frontal	L	0.07	0.09	0.04
	R	0.07	0.01	0.01
superior parietal	L	0.03	0.09	0.02
	R	0.02	0.07	-0.04
superior temporal	L	1.95E-03	-0.15	-0.05
	R	-0.01	-0.22	-0.11
supramarginal gyrus	L	0.04	0.07	0.04
	R	0.01	0.12	0.05
frontal pole	L	0.04	-0.01	0.06
	R	-0.01	0.03	0.01
temporal pole	L	1.44E-03	-0.04	-0.09
	R	-0.02	-0.05	-0.01
transverse temporal	L	0.03	0.03	0.02
	R	-0.02	-0.10	0.12
insula	L	-0.10	-0.03	-0.01
	R	-0.07	-0.04	-0.05
**Cortical Surface Area**				
banks superior temporal sulcus	L	-0.07	-0.05	0.17
	R	-0.04	-0.21	0.04
caudal anterior cingulate	L	-0.01	-0.06	-0.06
	R	-0.03	-0.05	-0.01
caudal middle frontal	L	1.92E-03	0.06	0.05

© 2023 American Medical Association. All rights reserved.

Region	Hemi	Cohen's *d*<br><br>All CHR-P vs Healthy individuals	Cohen's *d*<br><br>CHR-PC vs Healthy individuals	Cohen's *d*<br><br>medicated CHR-P vs unmedicated CHR-P
	R	0.01	0.07	2.71E-03
cuneus	L	-0.06	-0.05	0.06
	R	-0.04	-0.05	-0.08
entorhinal cortex	L	-0.08	-0.13	0.03
	R	-0.03	0.08	0.21
fusiform gyrus	L	-0.03	-0.03	0.01
	R	-0.05	-0.01	-0.01
inferior parietal	L	-0.06	0.07	-0.07
	R	-0.06	-0.05	0.03
inferior temporal	L	3.78E-03	0.04	0.04
	R	-0.04	0.02	0.04
isthmus cingulate	L	0.03	-0.04	-0.09
	R	0.08	0.08	0.12
lateral occipital	L	0.01	-0.03	-1.50E-03
	R	0.02	-0.04	-0.02
lateral orbitofrontal	L	0.02	-0.04	0.04
	R	-0.02	-0.02	-0.05
lingual gyrus	L	-0.03	-0.08	-0.02
	R	0.02	-0.07	-0.06
medial orbitofrontal	L	-0.06	-0.01	0.01
	R	-0.10	-0.09	0.05
middle temporal	L	0.06	0.07	0.03
	R	-0.01	-0.19	-0.06
parahippocampal	L	-0.02	0.09	-0.05
	R	0.04	0.03	-0.04
paracentral	L	0.04	0.18	-0.01
	R	0.07	0.02	-0.04
pars opercularis	L	-0.06	-0.02	-0.02
	R	0.02	0.03	0.05
pars orbitalis	L	0.06	0.12	0.05
	R	0.07	0.04	0.05

© 2023 American Medical Association. All rights reserved.

Region	Hemi	Cohen’s *d*<br><br>All CHR-P vs Healthy individuals	Cohen’s *d*<br><br>CHR-PC vs Healthy individuals	Cohen’s *d*<br><br>medicated CHR-P vs unmedicated CHR-P
pars triangularis	L	-0.04	2.41E-03	0.06
	R	0.04	0.16	-0.01
pericalcarine	L	-0.04	-0.10	-0.04
	R	-0.06	-0.15	-0.01
postcentral	L	0.06	0.12	0.03
	R	-0.02	-0.02	-0.01
posterior cingulate	L	-0.02	0.06	0.08
	R	-0.03	0.14	-0.01
precentral	L	0.09	0.04	-0.02
	R	0.07	0.13	-0.04
precuneus	L	0.03	0.06	0.01
	R	0.01	0.04	0.08
rostral anterior cingulate	L	-0.09	-0.11	-0.07
	R	-0.04	0.04	0.03
rostral middle frontal	L	1.31E-03	0.17	0.03
	R	0.04	0.13	0.02
superior frontal	L	0.01	0.01	-0.06
	R	-0.08	-0.05	0.02
superior parietal	L	0.05	0.07	-0.01
	R	0.06	0.12	-0.01
superior temporal	L	-2.16E-05	-0.08	-0.05
	R	-0.02	-0.17	0.04
supramarginal gyrus	L	-0.05	-0.11	0.02
	R	-0.03	-0.02	0.08
frontal pole	L	0.04	0.11	-0.05
	R	0.04	0.11	-0.10
temporal pole	L	-0.01	-0.04	-0.02
	R	0.02	0.05	-0.03
transverse temporal	L	-0.03	-0.02	0.02
	R	0.01	0.10	-0.10
insula	L	-0.01	-0.12	-0.05

© 2023 American Medical Association. All rights reserved.

Region	Hemi	Cohen's *d*<br><br>All CHR-P vs Healthy individuals	Cohen's *d*<br><br>CHR-PC vs Healthy individuals	Cohen's *d*<br><br>medicated CHR-P vs unmedicated CHR-P
	R	-0.03	-0.12	0.04

ASD_{CT}=average deviation score-cortical thickness; ADS_G=Average deviation score-global; ADS_{SA}=average deviation score-surface area; ADS_{SV}=average deviation score-subcortical volume; CHR-P = clinical high-risk for psychosis; CHR-PC = clinical high-risk for psychosis converters; L=left; R=right

© 2023 American Medical Association. All rights reserved.

eTable 10. Percentage of Individuals With Infra- or Supranormal Normative Average Deviation Scores by Group

	Normative z-scores in HI (N = 1237)		Normative z-scores in CHR-P (N = 1340)	
**Region**	**infranormal z-scores (%)**	**supranormal z-scores (%)**	**infranormal z-scores (%)**	**supranormal z-scores (%)**
**Average Deviation Score (ADS) in the main manuscript**				
**ADS_G[a]**	1.70	2.18	4.18	2.01
**ADS_{SV}[a]**	1.62	2.59	3.13	2.69
**ADS_{CT}**	1.94	2.67	3.21	2.31
**ADS_{SA}[a]**	1.05	2.83	3.51	2.69
**Alternative ADS definitions**				
**P-ADS_G[b]**	1.29	1.94	1.12	3.96
**P-ADS_{SV}**	0	4.03	0	4.62
**P-ADS_{CT}**	1.13	2.51	1.19	3.58
**P-ADS_{SA}[b]**	1.37	1.70	1.72	3.81
**N-ADS_G[a]**	2.43	1.70	4.25	1.19
**N-ADS_{SV}[a]**	2.35	1.22	4.42	1.35
**N-ADS_{CT}[a]**	2.18	2.51	4.40	1.34
**N-ADS_{SA}**	2.99	0.65	4.25	0.90
**$\|ADS_G\|$[b]**	1.70	2.34	1.12	4.03
**$\|ADS_{SV}\|$[b]**	0.08	3.31	0.07	5.75
**$\|ADS_{CT}\|$[b]**	1.78	2.51	1.19	4.55
**$\|ADS_{SA}\|$[b]**	1.21	1.94	1.27	3.96

[a] significant two-proportion z-tests difference in infranormal z-scores between CHR-P and HI at $P_{FDR} < 0.05$; [b] significant two-proportion z-tests difference in supranormal z-scores between CHR-P and HI at $P_{FDR} < 0.05$; ADS_G=Average deviation score-global; ADS_{SA}=average deviation score-surface area; ADS_{SV}=average deviation score-subcortical volume; CHR-P = clinical high-risk for psychosis; ASD values preceded by P denoted sums of positive z-scores, ASD values preceded by N denote sums of negative z-scores and |ADS| denotes the sum of the absolute values of the z-score

© 2023 American Medical Association. All rights reserved.

eTable 11. Percentage of CHR-P With Infra- or Supranormal Normative Average Deviation Scores According to Medication Status

	Unmediated CHR-P Individuals (N = 1061)		Mediated CHR-P Individuals (N = 243)	
**Region**	**infranormal z-scores (%)**	**supranormal z-scores (%)**	**infranormal z-scores (%)**	**supranormal z-scores (%)**
**Average Deviation Score (ADS)**				
ADS_G	2.92	1.60	5.35	2.06
ADS_{SV}	1.98	2.07	4.11	2.88
ADS_{CT}	2.83	2.17	2.47	2.06
ADS_{SA}	2.83	2.36	2.47	2.88

[a] significant two-proportion z-tests difference in infranormal ADS between CHR-P with and without antipsychotic medication exposure at $P_{FDR} < 0.05$; [b] significant two-proportion z-tests difference in supranormal ADS between CHR-P with and without antipsychotic medication exposure at $P_{FDR} < 0.05$; ASD_{CT}=average deviation score-cortical thickness; ADS_G=Average deviation score-global; ADS_{SA}=average deviation score-surface area; ADS_{SV}=average deviation score-subcortical volume; CHR-P = clinical high-risk for psychosis; Hemi = hemisphere; L = left; R = right.

© 2023 American Medical Association. All rights reserved.

eTable 12. Associations Between Regional Normative *z* Scores and Positive Symptoms and IQ in CHR-P Individual

		Positive Symptoms		IQ	
**Region**	**Hemi**	**β Estimate**	**Uncorrected P values**	**β Estimate**	**Uncorrected P values**
**Subcortical Volume**					
nucleus accumbens	L	1.53E-03	0.96	0.02	0.64
	R	-0.02	0.46	0.01	0.73
amygdala	L	-0.01	0.73	0.03	0.32
	R	1.81E-03	0.95	0.02	0.55
caudate	L	-0.02	0.53	0.11	6.00E-04[a]
	R	-0.03	0.24	0.10	2.60E-03
hippocampus	L	-0.04	0.20	-0.01	0.73
	R	-0.03	0.21	-0.01	0.79
pallidum	L	-0.01	0.66	0.03	0.43
	R	-0.02	0.49	-0.01	0.79
putamen	L	0.01	0.78	-0.02	0.58
	R	-0.01	0.61	1.92E-03	0.95
thalamus	L	-0.03	0.32	0.07	0.02
	R	-0.02	0.38	0.06	0.07
**Cortical Thickness**					
banks superior temporal sulcus	L	0.01	0.82	0.01	0.85
	R	0.01	0.73	-0.03	0.40
caudal anterior cingulate	L	-0.02	0.57	-0.01	0.68
	R	-0.01	0.73	0.03	0.36
caudal middle frontal	L	-0.04	0.19	0.05	0.15
	R	0.01	0.64	0.05	0.15
cuneus	L	-0.03	0.28	0.06	0.06
	R	0.01	0.69	0.04	0.25
entorhinal cortex	L	-0.02	0.49	0.05	0.14
	R	0.03	0.22	0.04	0.26
fusiform gyrus	L	-0.04	0.16	0.03	0.30
	R	-0.02	0.46	-3.01E-03	0.93
inferior parietal	L	0.01	0.63	-0.06	0.07
	R	0.01	0.82	-0.07	0.04
inferior temporal	L	-0.02	0.58	-3.98E-03	0.90
	R	-0.03	0.25	0.03	0.37
isthmus cingulate	L	-5.49E-05	1.00	-0.02	0.51
	R	-0.03	0.23	0.03	0.37
lateral occipital	L	-0.07	0.02	-0.06	0.10
	R	-0.04	0.11	-0.02	0.47
lateral orbitofrontal	L	-0.03	0.30	-0.02	0.53
	R	-3.76E-03	0.89	0.03	0.35

© 2023 American Medical Association. All rights reserved.

		Positive Symptoms		IQ	
Region	Hemi	β Estimate	Uncorrected P values	β Estimate	Uncorrected P values
lingual gyrus	L	0.02	0.52	0.08	0.02
	R	-0.01	0.74	0.05	0.13
medial orbitofrontal	L	1.69E-03	0.95	-0.04	0.20
	R	0.02	0.45	-0.02	0.57
middle temporal	L	-3.07E-03	0.91	-0.01	0.73
	R	0.02	0.43	-0.04	0.18
parahippocampal	L	0.01	0.71	0.04	0.19
	R	-1.02E-03	0.97	-0.01	0.80
paracentral	L	-0.01	0.79	-0.01	0.86
	R	0.04	0.11	-0.05	0.10
pars opercularis	L	0.02	0.46	3.03E-03	0.93
	R	-0.01	0.80	0.08	0.02
pars orbitalis	L	-0.01	0.73	-0.02	0.50
	R	-0.03	0.30	-0.03	0.30
pars triangularis	L	0.02	0.48	0.02	0.59
	R	0.04	0.17	-0.03	0.42
pericalcarine	L	0.01	0.67	0.03	0.33
	R	0.01	0.81	0.02	0.50
postcentral	L	-0.02	0.47	0.05	0.18
	R	0.06	0.04	0.05	0.14
posterior cingulate	L	0.04	0.12	-0.01	0.84
	R	-0.02	0.54	-0.02	0.64
precentral	L	-0.01	0.60	0.05	0.11
	R	0.01	0.74	0.02	0.64
precuneus	L	0.02	0.38	0.06	0.09
	R	0.02	0.37	-0.03	0.29
rostral anterior cingulate	L	0.02	0.57	-0.01	0.68
	R	3.15E-03	0.91	-0.03	0.43
rostral middle frontal	L	-0.03	0.25	-0.02	0.60
	R	-0.01	0.67	-0.05	0.10
superior frontal	L	-0.01	0.79	-0.02	0.46
	R	-0.01	0.79	-0.01	0.67
superior parietal	L	-0.01	0.83	-0.08	0.02
	R	0.05	0.09	-0.03	0.41
superior temporal	L	-0.02	0.43	0.03	0.37
	R	0.03	0.34	0.04	0.25
supramarginal gyrus	L	0.04	0.13	0.03	0.42
	R	0.06	0.03	0.01	0.67
frontal pole	L	0.01	0.69	-0.03	0.42
	R	-0.02	0.45	0.04	0.27
temporal pole	L	0.03	0.36	2.99E-03	0.93

© 2023 American Medical Association. All rights reserved.

		Positive Symptoms		IQ	
Region	Hemi	β Estimate	Uncorrected P values	β Estimate	Uncorrected P values
	R	0.02	0.42	0.04	0.25
transverse temporal	L	-0.01	0.83	-0.03	0.39
	R	0.03	0.23	0.01	0.84
insula	L	-3.09E-04	0.99	-0.02	0.57
	R	-2.04E-03	0.94	0.07	0.03
**Surface Area**					
banks superior temporal	L	0.02	0.53	-0.01	0.83
sulcus	R	0.02	0.54	-0.07	0.05
caudal anterior cingulate	L	-0.01	0.59	0.03	0.39
	R	-0.01	0.66	0.05	0.17
caudal middle frontal	L	-0.06	0.04	0.04	0.28
	R	-3.80E-03	0.89	0.01	0.80
cuneus	L	-2.72E-03	0.92	0.11	5.64E-04[a]
	R	0.01	0.76	0.02	0.53
entorhinal cortex	L	1.22E-03	0.96	0.02	0.63
	R	-0.01	0.80	4.72E-03	0.89
fusiform gyrus	L	0.03	0.24	0.09	0.01
	R	0.03	0.27	0.05	0.15
inferior parietal	L	0.05	0.08	-0.03	0.30
	R	-0.01	0.80	-0.01	0.83
inferior temporal	L	0.02	0.53	0.04	0.23
	R	-3.74E-03	0.89	0.07	0.03
isthmus cingulate	L	-3.82E-03	0.89	-0.04	0.24
	R	0.03	0.22	-0.02	0.56
lateral occipital	L	0.01	0.66	0.01	0.66
	R	-1.66E-03	0.95	0.06	0.06
lateral orbitofrontal	L	-6.00E-04	0.98	4.52E-03	0.89
	R	0.01	0.67	0.02	0.58
lingual gyrus	L	-0.01	0.84	0.07	0.03
	R	-0.02	0.46	0.04	0.18
medial orbitofrontal	L	-0.02	0.51	0.04	0.22
	R	-0.04	0.13	0.04	0.21
middle temporal	L	0.02	0.45	-0.01	0.79
	R	0.03	0.31	0.02	0.50
parahippocampal	L	-0.03	0.35	0.02	0.53
	R	-0.05	0.06	4.24E-03	0.90
paracentral	L	0.03	0.30	-0.06	0.06
	R	-0.05	0.08	-0.08	0.02
pars opercularis	L	-0.01	0.78	0.03	0.37
	R	-2.56E-03	0.93	0.04	0.25
pars orbitalis	L	-0.06	0.04	0.05	0.11

© 2023 American Medical Association. All rights reserved.

		Positive Symptoms		IQ	
Region	Hemi	β Estimate	Uncorrected P values	β Estimate	Uncorrected P values
	R	-0.05	0.10	0.06	0.06
pars triangularis	L	-0.02	0.44	0.03	0.36
	R	-0.02	0.46	0.01	0.67
pericalcarine	L	0.01	0.81	0.08	0.02
	R	3.51E-03	0.90	0.02	0.61
postcentral	L	-0.03	0.28	0.01	0.70
	R	-0.03	0.25	-0.02	0.61
posterior cingulate	L	-2.80E-03	0.92	-0.01	0.68
	R	0.01	0.76	0.01	0.70
precentral	L	-0.04	0.16	0.02	0.61
	R	-0.01	0.60	-1.18E-03	0.97
precuneus	L	0.01	0.75	-0.09	0.00
	R	-0.02	0.44	0.02	0.63
rostral anterior cingulate	L	-0.04	0.20	0.09	0.01
	R	-0.04	0.13	8.64E-04	0.98
rostral middle frontal	L	0.02	0.52	0.04	0.29
	R	0.02	0.39	0.03	0.44
superior frontal	L	0.01	0.78	-3.30E-04	0.99
	R	-0.03	0.27	-0.01	0.84
superior parietal	L	-0.02	0.48	-0.02	0.50
	R	-0.01	0.84	-0.04	0.19
superior temporal	L	-0.03	0.31	-0.03	0.30
	R	0.03	0.29	-0.04	0.27
supramarginal gyrus	L	-0.03	0.21	-0.03	0.36
	R	-0.03	0.21	-0.03	0.45
frontal pole	L	-0.03	0.33	-0.01	0.81
	R	0.01	0.82	-0.01	0.74
temporal pole	L	0.02	0.43	-0.01	0.67
	R	4.22E-03	0.88	-0.06	0.09
transverse temporal	L	-0.03	0.22	-0.03	0.41
	R	-0.01	0.78	-0.04	0.26
insula	L	-0.02	0.55	-0.01	0.69
	R	-0.05	0.08	-0.04	0.23

[a] significant at $P_{FDR}<0.05$; hemi = hemisphere; CHR-P = clinical high-risk for psychosis; IQ = intelligence quotient; L = left; R = right

© 2023 American Medical Association. All rights reserved.

eTable 13. Associations Between Observed Brain Morphometric Measures With Positive Symptoms and IQ in CHR-P

		Positive Symptoms Score		IQ	
**Region**	**Hemi**	**β Estimate**	**Uncorrected P values**	**β Estimate**	**Uncorrected P values**
**Subcortical Volume**					
nucleus accumbens	L	-4.90E-03	0.85	0.01	0.83
	R	-0.02	0.46	0.01	0.74
amygdala	L	-0.01	0.76	0.02	0.39
	R	-0.01	0.80	0.01	0.67
caudate	L	-0.01	0.55	0.09	1.24E-03
	R	-0.03	0.19	0.08	3.42E-03
hippocampus	L	-0.04	0.06	-0.02	0.44
	R	-0.04	0.08	-0.01	0.61
pallidum	L	3.14E-03	0.90	0.03	0.37
	R	-0.02	0.45	-0.01	0.83
putamen	L	-5.93E-04	0.98	-0.02	0.53
	R	-0.02	0.40	-0.01	0.75
thalamus	L	-0.03	0.10	0.05	0.07
	R	-0.03	0.19	0.03	0.17
**Cortical Thickness**					
banks superior temporal sulcus	L	0.01	0.68	-4.95E-03	0.86
	R	0.01	0.58	-0.03	0.33
caudal anterior cingulate	L	-0.01	0.81	-0.03	0.41
	R	-0.01	0.65	0.03	0.39
caudal middle frontal	L	-0.04	0.04	0.02	0.30
	R	-0.01	0.77	0.03	0.23
cuneus	L	-0.03	0.25	0.06	0.04
	R	0.01	0.82	0.04	0.17
entorhinal cortex	L	-0.01	0.64	0.06	0.08
	R	0.05	0.07	0.03	0.34
fusiform gyrus	L	-0.02	0.25	0.03	0.25
	R	-0.01	0.59	0.01	0.75
inferior parietal	L	8.91E-04	0.96	-0.04	0.04
	R	4.69E-03	0.78	-0.05	0.01
inferior temporal	L	-0.01	0.57	-0.01	0.72
	R	-0.02	0.34	0.02	0.53
isthmus cingulate	L	0.01	0.79	-0.02	0.48
	R	-0.03	0.25	0.02	0.50
lateral occipital	L	-0.05	0.01	-0.03	0.21
	R	-0.04	0.06	-0.01	0.58
lateral orbitofrontal	L	-0.02	0.41	-0.02	0.53

© 2023 American Medical Association. All rights reserved.

		Positive Symptoms Score		IQ	
Region	Hemi	β Estimate	Uncorrected P values	β Estimate	Uncorrected P values
	R	-4.99E-03	0.82	0.03	0.34
lingual gyrus	L	0.01	0.56	0.08	0.01
	R	-0.01	0.60	0.05	0.06
medial orbitofrontal	L	-3.64E-03	0.88	-0.03	0.26
	R	0.01	0.82	-0.02	0.57
middle temporal	L	0.01	0.74	-0.01	0.64
	R	0.02	0.29	-0.04	0.10
parahippocampal	L	0.02	0.48	0.04	0.23
	R	0.01	0.80	-0.01	0.84
paracentral	L	-0.01	0.64	-0.01	0.71
	R	0.03	0.15	-0.04	0.09
pars opercularis	L	0.01	0.74	4.25E-04	0.99
	R	-0.01	0.79	0.05	0.05
pars orbitalis	L	-0.01	0.65	-0.02	0.56
	R	-0.02	0.38	-0.04	0.17
pars triangularis	L	0.02	0.43	0.01	0.78
	R	0.03	0.15	-0.02	0.33
pericalcarine	L	0.01	0.64	0.03	0.29
	R	4.48E-03	0.87	0.02	0.46
postcentral	L	-0.02	0.44	0.03	0.29
	R	0.05	0.03	0.03	0.18
posterior cingulate	L	0.04	0.07	-0.02	0.54
	R	-0.02	0.51	-0.03	0.32
precentral	L	-0.01	0.53	0.03	0.15
	R	1.98E-03	0.92	0.01	0.70
precuneus	L	0.01	0.40	0.03	0.19
	R	0.01	0.46	-0.03	0.20
rostral anterior cingulate	L	0.01	0.58	-0.02	0.54
	R	1.13E-03	0.96	-0.02	0.60
rostral middle frontal	L	-0.03	0.08	-0.01	0.66
	R	-0.01	0.56	-0.04	0.07
superior frontal	L	-0.01	0.45	-0.03	0.16
	R	-0.01	0.42	-0.02	0.29
superior parietal	L	-0.01	0.74	-0.05	0.01
	R	0.03	0.09	-0.02	0.26
superior temporal	L	-0.02	0.29	0.02	0.47
	R	0.02	0.35	0.02	0.44
supramarginal gyrus	L	0.02	0.24	0.01	0.61
	R	0.04	0.02	0.01	0.79
frontal pole	L	0.01	0.76	-0.03	0.27
	R	-0.03	0.28	0.03	0.34

© 2023 American Medical Association. All rights reserved.

		Positive Symptoms Score		IQ	
Region	Hemi	β Estimate	Uncorrected P values	β Estimate	Uncorrected P values
temporal pole	L	0.03	0.30	4.79E-03	0.88
	R	0.03	0.26	0.04	0.18
transverse temporal	L	1.23E-04	1.00	-0.02	0.42
	R	0.03	0.27	4.82E-03	0.87
insula	L	-0.01	0.59	-0.01	0.84
	R	-0.01	0.64	0.07	0.01
**Surface Area**					
banks superior temporal sulcus	L	0.01	0.71	-2.35E-03	0.94
	R	0.01	0.56	-0.06	0.03
caudal anterior cingulate	L	-0.01	0.62	0.01	0.67
	R	-0.01	0.72	0.02	0.49
caudal middle frontal	L	-0.04	0.05	0.02	0.44
	R	-1.95E-05	1.00	0.02	0.54
cuneus	L	2.41E-03	0.92	0.09	9.12E-04
	R	0.01	0.60	0.02	0.58
entorhinal cortex	L	0.01	0.72	0.02	0.62
	R	0.01	0.65	-3.46E-03	0.91
fusiform gyrus	L	0.03	0.11	0.05	0.03
	R	0.03	0.12	0.02	0.43
inferior parietal	L	0.05	0.03	-0.02	0.44
	R	-0.01	0.62	-0.02	0.48
inferior temporal	L	0.02	0.40	0.01	0.64
	R	0.01	0.58	0.03	0.14
isthmus cingulate	L	-0.01	0.67	-0.02	0.36
	R	0.02	0.31	-0.02	0.55
lateral occipital	L	0.01	0.50	-2.82E-03	0.91
	R	0.01	0.74	0.03	0.19
lateral orbitofrontal	L	0.01	0.77	-3.43E-03	0.87
	R	4.74E-03	0.80	2.60E-03	0.91
lingual gyrus	L	2.16E-04	0.99	0.05	0.08
	R	-0.01	0.73	0.04	0.14
medial orbitofrontal	L	-0.01	0.72	0.02	0.47
	R	-0.02	0.36	0.01	0.58
middle temporal	L	0.01	0.45	-0.02	0.48
	R	0.02	0.16	0.01	0.73
parahippocampal	L	-0.02	0.42	0.01	0.72
	R	-0.04	0.14	-0.01	0.79
paracentral	L	0.03	0.24	-0.05	0.09
	R	-0.04	0.09	-0.06	0.02
pars opercularis	L	-3.97E-03	0.86	0.01	0.71
	R	-2.81E-03	0.91	0.04	0.14

© 2023 American Medical Association. All rights reserved.

		Positive Symptoms Score		IQ	
Region	Hemi	β Estimate	Uncorrected P values	β Estimate	Uncorrected P values
pars orbitalis	L	-0.04	0.06	0.04	0.14
	R	-0.03	0.16	0.05	0.08
pars triangularis	L	-0.01	0.61	0.03	0.28
	R	-0.02	0.34	0.02	0.50
pericalcarine	L	0.01	0.76	0.07	0.02
	R	2.92E-04	0.99	0.02	0.54
postcentral	L	-0.01	0.41	1.78E-03	0.94
	R	-0.01	0.62	-0.02	0.46
posterior cingulate	L	-3.34E-03	0.88	-0.02	0.51
	R	0.01	0.63	-0.01	0.81
precentral	L	-0.02	0.27	0.01	0.80
	R	-0.01	0.76	0.01	0.82
precuneus	L	0.01	0.76	-0.06	3.67E-03
	R	-0.01	0.54	0.01	0.81
rostral anterior cingulate	L	-0.02	0.29	0.06	0.01
	R	-0.03	0.15	-0.01	0.63
rostral middle frontal	L	0.01	0.59	0.01	0.48
	R	0.01	0.65	0.01	0.81
superior frontal	L	0.01	0.74	5.10E-04	0.98
	R	-0.01	0.38	-4.34E-03	0.83
superior parietal	L	-0.01	0.59	-0.01	0.63
	R	-4.29E-03	0.83	-0.03	0.17
superior temporal	L	-0.01	0.49	-0.03	0.26
	R	0.02	0.19	-0.03	0.20
supramarginal gyrus	L	-0.02	0.32	-0.03	0.23
	R	-0.02	0.22	-0.01	0.74
frontal pole	L	-0.01	0.77	0.01	0.74
	R	0.01	0.73	-5.70E-05	1.00
temporal pole	L	0.02	0.48	-0.01	0.65
	R	0.02	0.45	-0.06	0.05
transverse temporal	L	-0.02	0.39	-0.03	0.35
	R	0.01	0.80	-0.03	0.33
insula	L	0.01	0.77	-0.01	0.54
	R	-0.02	0.29	-0.03	0.22

[a] significant at P_{FDR}<0.05; Hemi = hemisphere; CHR-P = clinical high-risk for psychosis; IQ = intelligence quotient; L = left; R = right

© 2023 American Medical Association. All rights reserved.

eTable 14. Associations Between Either Regional *z* Scores or Observed Brain Morphometric Measures With IQ in Healthy Individuals

		Associations with normative z-scores		Associations with observed values	
**Region**	**Hemi**	**β Estimate**	**Uncorrected P values**	**β Estimate**	**Uncorrected P values**
**Subcortical Volume**					
nucleus accumbens	L	-0.01	0.81	-0.02	0.50
	R	0.01	0.85	4.61E-03	0.89
amygdala	L	-0.01	0.69	-0.02	0.62
	R	-0.02	0.52	-0.01	0.66
caudate	L	0.03	0.47	0.02	0.51
	R	0.03	0.34	0.02	0.42
hippocampus	L	0.02	0.56	0.02	0.53
	R	0.05	0.20	0.03	0.26
pallidum	L	-0.03	0.33	-0.01	0.66
	R	0.03	0.40	0.04	0.18
putamen	L	2.87E-04	0.99	9.12E-04	0.98
	R	-3.88E-03	0.91	-2.65E-03	0.93
thalamus	L	0.02	0.61	0.02	0.54
	R	0.06	0.08	0.04	0.14
**Cortical Thickness**					
banks superior temporal sulcus	L	0.01	0.85	0.01	0.85
	R	-0.04	0.29	-0.03	0.29
caudal anterior cingulate	L	-0.05	0.19	-0.04	0.19
	R	-0.08	0.02	-0.08	0.02
caudal middle frontal	L	0.03	0.39	0.03	0.24
	R	0.05	0.21	0.04	0.13
cuneus	L	0.10	0.01	0.08	0.01
	R	0.07	0.04	0.06	0.06
entorhinal cortex	L	0.02	0.65	0.02	0.59
	R	-4.52E-03	0.90	-1.31E-03	0.97
fusiform gyrus	L	-0.04	0.22	-0.02	0.50
	R	-0.04	0.28	-0.02	0.60
inferior parietal	L	-0.10	3.71E-03	-0.06	0.01
	R	-0.07	0.04	-0.04	0.05
inferior temporal	L	-0.03	0.34	-0.03	0.34
	R	-4.64E-03	0.90	-0.01	0.76
isthmus cingulate	L	-0.02	0.57	-0.01	0.80
	R	-0.04	0.24	-0.02	0.61
lateral occipital	L	-0.10	0.01	-0.06	0.04
	R	-4.14E-04	0.99	0.01	0.76

© 2023 American Medical Association. All rights reserved.

		Associations with normative z-scores		Associations with observed values	
Region	Hemi	β Estimate	Uncorrected P values	β Estimate	Uncorrected P values
lateral orbitofrontal	L	0.02	0.66	0.01	0.81
	R	-0.02	0.49	-0.02	0.45
lingual gyrus	L	0.11	1.37E-03	0.09	2.50E-03
	R	0.08	0.02	0.07	0.02
medial orbitofrontal	L	-0.01	0.82	-0.02	0.58
	R	0.01	0.71	0.01	0.84
middle temporal	L	-0.05	0.16	-0.04	0.19
	R	-0.06	0.07	-0.05	0.04
parahippocampal	L	-0.03	0.39	-0.03	0.36
	R	-0.01	0.82	-0.01	0.72
paracentral	L	0.04	0.29	0.03	0.27
	R	-0.01	0.82	-0.02	0.51
pars opercularis	L	0.01	0.84	0.02	0.58
	R	0.06	0.12	0.05	0.09
pars orbitalis	L	-0.02	0.55	-0.02	0.63
	R	-0.04	0.31	-0.03	0.23
pars triangularis	L	-0.01	0.77	5.32E-04	0.98
	R	0.02	0.64	0.01	0.73
pericalcarine	L	0.10	0.01	0.07	0.04
	R	0.08	0.02	0.06	0.09
postcentral	L	0.09	0.02	0.06	0.02
	R	0.09	0.01	0.08	0.01
posterior cingulate	L	-2.35E-03	0.95	0.01	0.86
	R	0.01	0.73	0.02	0.53
precentral	L	0.05	0.14	0.03	0.22
	R	0.04	0.33	0.01	0.63
precuneus	L	-0.01	0.83	0.01	0.79
	R	-0.06	0.10	-0.02	0.33
rostral anterior cingulate	L	-4.14E-03	0.91	-0.01	0.86
	R	-0.05	0.19	-0.03	0.31
rostral middle frontal	L	-0.03	0.40	-0.01	0.71
	R	-0.03	0.47	-0.02	0.49
superior frontal	L	-0.07	0.04	-0.04	0.07
	R	-0.08	0.02	-0.04	0.04
superior parietal	L	0.01	0.77	8.89E-04	0.97
	R	-2.07E-04	1.00	4.24E-03	0.85
superior temporal	L	0.02	0.53	0.02	0.43
	R	0.04	0.28	0.03	0.32
supramarginal gyrus	L	-0.02	0.55	-0.02	0.50
	R	0.05	0.20	0.04	0.14

© 2023 American Medical Association. All rights reserved.

		Associations with normative z-scores		Associations with observed values	
Region	Hemi	β Estimate	Uncorrected P values	β Estimate	Uncorrected P values
frontal pole	L	-0.03	0.44	-0.01	0.83
	R	-0.04	0.30	-0.04	0.26
temporal pole	L	0.06	0.08	0.06	0.09
	R	0.03	0.44	0.02	0.56
transverse temporal	L	0.01	0.85	0.01	0.76
	R	0.05	0.14	0.05	0.09
insula	L	0.07	0.05	0.07	0.02
	R	0.04	0.25	0.05	0.11
**Surface Area**					
banks superior temporal sulcus	L	-0.03	0.40	-0.02	0.45
	R	-0.03	0.40	-0.02	0.63
caudal anterior cingulate	L	0.04	0.27	0.06	0.09
	R	-0.02	0.56	-0.02	0.48
caudal middle frontal	L	0.09	0.01	0.07	0.01
	R	-9.58E-04	0.98	3.75E-03	0.90
cuneus	L	-0.02	0.62	-0.01	0.72
	R	-0.01	0.73	-0.02	0.40
entorhinal cortex	L	0.05	0.13	0.03	0.30
	R	0.02	0.66	0.01	0.69
fusiform gyrus	L	0.01	0.87	4.26E-03	0.87
	R	-0.02	0.50	-0.02	0.49
inferior parietal	L	-1.32E-03	0.97	2.26E-03	0.94
	R	-0.05	0.13	-0.04	0.15
inferior temporal	L	0.03	0.47	-0.01	0.80
	R	-0.01	0.81	-0.03	0.25
isthmus cingulate	L	-1.60E-06	1.00	1.46E-03	0.96
	R	0.03	0.34	0.02	0.48
lateral occipital	L	-0.02	0.62	-0.02	0.36
	R	0.04	0.26	0.01	0.68
lateral orbitofrontal	L	-0.01	0.76	-0.01	0.63
	R	0.02	0.65	0.02	0.53
lingual gyrus	L	0.02	0.54	0.02	0.49
	R	-0.01	0.82	1.48E-03	0.96
medial orbitofrontal	L	0.02	0.66	2.92E-03	0.91
	R	-4.35E-03	0.90	-0.02	0.53
middle temporal	L	0.01	0.76	-3.68E-03	0.88
	R	0.03	0.47	0.01	0.81
parahippocampal	L	0.08	0.03	0.08	0.01
	R	-0.01	0.72	2.47E-04	0.99
paracentral	L	1.19E-03	0.97	3.49E-06	1.00

© 2023 American Medical Association. All rights reserved.

Region	Hemi	Associations with normative z-scores		Associations with observed values	
		β Estimate	Uncorrected P values	β Estimate	Uncorrected P values
	R	3.84E-03	0.91	3.03E-03	0.92
pars opercularis	L	0.01	0.80	0.01	0.76
	R	-4.79E-03	0.89	-6.62E-04	0.98
pars orbitalis	L	-0.04	0.22	-0.04	0.11
	R	0.01	0.79	0.01	0.75
pars triangularis	L	0.02	0.51	0.02	0.46
	R	0.05	0.20	0.04	0.24
pericalcarine	L	-3.03E-03	0.93	-3.48E-03	0.91
	R	-0.01	0.71	-0.01	0.77
postcentral	L	0.08	0.02	0.06	0.01
	R	0.06	0.12	0.04	0.09
posterior cingulate	L	0.05	0.13	0.04	0.20
	R	0.02	0.49	0.01	0.71
precentral	L	-3.51E-03	0.92	-0.01	0.82
	R	0.02	0.57	0.01	0.56
precuneus	L	-0.03	0.45	-0.01	0.59
	R	0.05	0.18	0.03	0.18
rostral anterior cingulate	L	0.01	0.80	4.90E-03	0.85
	R	-0.03	0.46	-0.02	0.39
rostral middle frontal	L	0.05	0.19	0.01	0.59
	R	0.01	0.81	-1.52E-04	0.99
superior frontal	L	0.01	0.83	-4.96E-03	0.82
	R	9.11E-04	0.98	-0.01	0.76
superior parietal	L	0.01	0.74	0.01	0.69
	R	-6.40E-04	0.99	3.44E-03	0.90
superior temporal	L	-0.06	0.11	-0.03	0.15
	R	1.24E-03	0.97	0.01	0.80
supramarginal gyrus	L	-0.04	0.27	-0.03	0.21
	R	-0.06	0.12	-0.03	0.22
frontal pole	L	0.06	0.10	0.04	0.16
	R	-0.02	0.62	-0.02	0.53
temporal pole	L	0.01	0.81	-7.80E-04	0.98
	R	-0.01	0.70	-0.01	0.77
transverse temporal	L	-0.06	0.12	-0.04	0.17
	R	-2.75E-04	0.99	-1.78E-03	0.95
insula	L	-0.02	0.51	-0.02	0.33
	R	-0.02	0.57	-0.02	0.52

[a] significant at P_{FDR}<0.05; Hemi = hemisphere; IQ = intelligence quotient; L = left; R = right

© 2023 American Medical Association. All rights reserved.

eTable 15. Associations Between Regional *z* Scores and Average Deviation Scores With Positive Symptoms and IQ in CHR-P Adding Site as a Random Effect

		Positive Symptoms		IQ	
**Region**	**Hemi**	**β Estimate**	**Uncorrected P values**	**β Estimate**	**Uncorrected P values**
**Subcortical Volume**					
nucleus accumbens	L	6.88E-04	0.98	0.02	0.63
	R	-0.02	0.45	0.01	0.76
amygdala	L	-0.01	0.73	0.03	0.32
	R	1.81E-03	0.95	0.02	0.55
caudate	L	-0.02	0.53	0.11	6.00E-04[a]
	R	-0.03	0.23	0.10	3.27E-03
hippocampus	L	-0.04	0.20	-0.01	0.73
	R	-0.03	0.21	-0.01	0.79
pallidum	L	-0.01	0.68	0.03	0.43
	R	-0.02	0.49	-0.01	0.79
putamen	L	0.01	0.78	-0.02	0.56
	R	-0.01	0.61	1.92E-03	0.95
thalamus	L	-0.03	0.32	0.07	0.02
	R	-0.02	0.38	0.06	0.07
**Cortical Thickness**					
banks superior temporal sulcus	L	0.01	0.82	0.82	0.82
	R	0.01	0.73	0.73	0.73
caudal anterior cingulate	L	-0.02	0.57	0.57	0.57
	R	-0.01	0.73	0.73	0.73
caudal middle frontal	L	-0.04	0.19	0.19	0.19
	R	0.01	0.64	0.64	0.64
cuneus	L	-0.03	0.28	0.28	0.28
	R	0.01	0.69	0.69	0.69
entorhinal cortex	L	-0.02	0.49	0.49	0.49
	R	0.03	0.22	0.22	0.22
fusiform gyrus	L	-0.04	0.16	0.16	0.16

© 2023 American Medical Association. All rights reserved.

		Positive Symptoms		IQ	
Region	Hemi	β Estimate	Uncorrected P values	β Estimate	Uncorrected P values
	R	-0.02	0.46	0.46	0.46
inferior parietal	L	0.01	0.63	0.63	0.63
	R	0.01	0.82	0.82	0.82
inferior temporal	L	-0.02	0.58	0.58	0.58
	R	-0.03	0.26	0.26	0.26
isthmus cingulate	L	-5.49E-05	1.00	1.00	1.00
	R	-0.03	0.23	0.23	0.23
lateral occipital	L	-0.07	0.02	0.02	0.02
	R	-0.04	0.11	0.11	0.11
lateral orbitofrontal	L	-0.03	0.30	0.30	0.30
	R	-3.76E-03	0.89	0.89	0.89
lingual gyrus	L	0.02	0.52	0.52	0.52
	R	-0.01	0.74	0.74	0.74
medial orbitofrontal	L	1.69E-03	0.95	0.95	0.95
	R	0.02	0.45	0.45	0.45
middle temporal	L	-3.07E-03	0.91	0.91	0.91
	R	0.02	0.43	0.43	0.43
parahippocampal	L	0.01	0.71	0.71	0.71
	R	-1.02E-03	0.97	0.97	0.97
paracentral	L	-0.01	0.79	0.79	0.79
	R	0.04	0.11	0.11	0.11
pars opercularis	L	0.02	0.46	0.46	0.46
	R	-0.01	0.80	0.80	0.80
pars orbitalis	L	-0.01	0.73	0.73	0.73
	R	-0.03	0.30	0.30	0.30
pars triangularis	L	0.02	0.48	0.48	0.48
	R	0.04	0.17	0.17	0.17
pericalcarine	L	0.01	0.67	0.67	0.67
	R	0.01	0.81	0.81	0.81

© 2023 American Medical Association. All rights reserved.

		Positive Symptoms		IQ	
**Region**	**Hemi**	**β Estimate**	**Uncorrected P values**	**β Estimate**	**Uncorrected P values**
postcentral	L	-0.02	0.47	0.47	0.47
	R	0.06	0.04	0.04	0.04
posterior cingulate	L	0.04	0.12	0.12	0.12
	R	-0.02	0.54	0.54	0.54
precentral	L	-0.02	0.59	0.59	0.59
	R	0.01	0.74	0.74	0.74
precuneus	L	0.02	0.38	0.38	0.38
	R	0.02	0.37	0.37	0.37
rostral anterior cingulate	L	0.02	0.57	0.57	0.57
	R	3.15E-03	0.91	0.91	0.91
rostral middle frontal	L	-0.03	0.25	0.25	0.25
	R	-0.01	0.67	0.67	0.67
superior frontal	L	-0.01	0.79	0.79	0.79
	R	-0.01	0.79	0.79	0.79
superior parietal	L	-0.01	0.85	0.85	0.85
	R	0.05	0.09	0.09	0.09
superior temporal	L	-0.02	0.43	0.43	0.43
	R	0.02	0.41	0.41	0.41
supramarginal gyrus	L	0.04	0.13	0.13	0.13
	R	0.06	0.03	0.03	0.03
frontal pole	L	0.01	0.69	0.69	0.69
	R	-0.02	0.45	0.45	0.45
temporal pole	L	0.03	0.36	0.36	0.36
	R	0.02	0.42	0.42	0.42
transverse temporal	L	-0.01	0.83	0.83	0.83
	R	0.03	0.23	0.23	0.23
insula	L	-1.80E-05	1.00	1.00	1.00
	R	-2.04E-03	0.94	0.94	0.94
**Surface Area**					

© 2023 American Medical Association. All rights reserved.

		Positive Symptoms		IQ	
Region	Hemi	β Estimate	Uncorrected P values	β Estimate	Uncorrected P values
banks superior temporal sulcus	L	0.02	0.52	-0.01	0.77
	R	0.02	0.56	-0.07	0.04
caudal anterior cingulate	L	-0.01	0.59	0.03	0.39
	R	-0.01	0.66	0.05	0.17
caudal middle frontal	L	-0.06	0.04	0.04	0.28
	R	-3.80E-03	0.89	0.01	0.80
cuneus	L	-2.72E-03	0.92	0.12	4.91E-04[a]
	R	0.01	0.76	0.02	0.54
entorhinal cortex	L	1.22E-03	0.96	0.02	0.63
	R	-0.01	0.80	4.72E-03	0.89
fusiform gyrus	L	0.04	0.21	0.08	0.01
	R	0.03	0.25	0.05	0.17
inferior parietal	L	0.05	0.08	-0.03	0.30
	R	-0.01	0.80	-0.01	0.83
inferior temporal	L	0.02	0.53	0.04	0.23
	R	-3.74E-03	0.89	0.07	0.03
isthmus cingulate	L	-3.82E-03	0.89	-0.04	0.24
	R	0.03	0.22	-0.02	0.56
lateral occipital	L	0.01	0.66	0.01	0.66
	R	-1.66E-03	0.95	0.06	0.06
lateral orbitofrontal	L	-6.00E-04	0.98	4.52E-03	0.89
	R	0.01	0.76	0.02	0.60
lingual gyrus	L	-0.01	0.84	0.07	0.03
	R	-0.02	0.46	0.04	0.18
medial orbitofrontal	L	-0.02	0.51	0.04	0.22
	R	-0.04	0.15	0.04	0.21
middle temporal	L	0.02	0.45	-0.01	0.79
	R	0.03	0.31	0.02	0.50
parahippocampal	L	-0.03	0.35	0.02	0.53

© 2023 American Medical Association. All rights reserved.

		Positive Symptoms		IQ	
Region	Hemi	β Estimate	Uncorrected P values	β Estimate	Uncorrected P values
	R	-0.05	0.08	3.33E-06	1.00
paracentral	L	0.03	0.30	-0.06	0.06
	R	-0.05	0.08	-0.08	0.02
pars opercularis	L	-0.01	0.78	0.03	0.37
	R	-2.56E-03	0.93	0.04	0.25
pars orbitalis	L	-0.06	0.04	0.05	0.13
	R	-0.05	0.10	0.06	0.06
pars triangularis	L	-0.02	0.44	0.03	0.36
	R	-0.02	0.46	0.01	0.67
pericalcarine	L	0.01	0.81	0.08	0.02
	R	3.51E-03	0.90	0.02	0.63
postcentral	L	-0.03	0.28	0.01	0.71
	R	-0.03	0.25	-0.02	0.61
posterior cingulate	L	-2.80E-03	0.92	-0.01	0.68
	R	0.01	0.76	0.01	0.70
precentral	L	-0.04	0.17	0.02	0.60
	R	-0.01	0.62	-1.27E-03	0.97
precuneus	L	0.01	0.75	-0.09	4.88E-03
	R	-0.02	0.44	0.02	0.63
rostral anterior cingulate	L	-0.04	0.20	0.09	0.01
	R	-0.04	0.13	-8.73E-04	0.98
rostral middle frontal	L	0.02	0.52	0.04	0.29
	R	0.02	0.39	0.03	0.44
superior frontal	L	0.01	0.78	-3.30E-04	0.99
	R	-0.03	0.27	-0.01	0.84
superior parietal	L	-0.02	0.48	-0.02	0.50
	R	-0.01	0.84	-0.04	0.19
superior temporal	L	-0.03	0.31	-0.03	0.30
	R	0.03	0.29	-0.04	0.27

© 2023 American Medical Association. All rights reserved.

		Positive Symptoms		IQ	
**Region**	**Hemi**	**β Estimate**	**Uncorrected P values**	**β Estimate**	**Uncorrected P values**
supramarginal gyrus	L	-0.03	0.21	-0.03	0.36
	R	-0.03	0.21	-0.03	0.45
frontal pole	L	-0.03	0.36	-4.44E-03	0.89
	R	2.85E-03	0.92	-0.01	0.78
temporal pole	L	0.02	0.43	-0.01	0.67
	R	4.22E-03	0.88	-0.06	0.09
transverse temporal	L	-0.03	0.22	-0.03	0.41
	R	-0.01	0.78	-0.04	0.26
insula	L	-0.02	0.56	-0.01	0.79
	R	-0.05	0.09	-0.04	0.25
**Average Deviation Scores**					
ADS_{G}		-0.04	0.18	0.09	0.01[a]
ADS_{CT}		0.01	0.83	0.04	0.25
ADS_{SA}		-0.05	0.07	0.08	0.01[a]
ADS_{SV}		-0.03	0.31	0.05	0.13

[a] significant at $P_{FDR}<0.05$; hemi = hemisphere; CHR-P = clinical high-risk for psychosis; IQ = intelligence quotient; L = left; R = right

© 2023 American Medical Association. All rights reserved.

eTable 16. Associations Between Average Deviation Scores With Positive Symptoms and IQ in CHR-P and Healthy Individuals

	CHR-P individuals				Healthy individuals	
**Average Deviation Scores**	**Positive Symptoms (N = 1340)**		**IQ (N = 924)**		**IQ (N = 797)**	
	**β Estimate**	**Uncorrected P-value**	**β Estimate**	**Uncorrected P-value**	**β Estimate**	**Uncorrected P-value**
**ADS in the main analyses**						
**ADS_G**	-0.05	0.08	0.10	3.25E-03[a]	0.05	0.14
**ADS_{SV}**	-0.03	0.31	0.05	0.13	0.02	0.57
**ADS_{CT}**	0.01	0.61	0.04	0.25	0.02	0.65
**ADS_{SA}**	-0.08	0.01[a]	0.09	0.01[a]	0.05	0.13
**Alternative ADS definitions**						
**P-ADS_G**	0.02	0.54	2.41E-03	0.94	-0.04	0.26
**P-ADS_{SV}**	-0.02	0.61	0.06	0.07	9.95E-03	0.80
**P-ADS_{CT}**	0.02	0.51	-0.09	7.96E-03[a]	-0.03	0.34
**P-ADS_{SA}**	0.01	0.67	0.69	0.04	-0.01	0.72
**N-ADS_G**	-0.02	0.38	0.06	0.05	0.03	0.46
**N-ADS_{SV}**	-0.04	0.14	0.04	0.27	0.05	0.18
**N-ADS_{CT}**	-0.01	0.71	0.10	1.37E-03[a]	0.01	0.72
**N-ADS_{SA}**	5.49E-03	0.84	-0.03	0.37	8.16E-03	0.82
**$\|ADS_G\|$**	0.02	0.38	-0.04	0.19	-0.04	0.29
**$\|ADS_{SV}\|$**	0.03	0.26	-0.02	0.61	-0.07	0.05
**$\|ADS_{CT}\|$**	0.02	0.52	-0.11	7.18E-04[a]	-0.03	0.40
**$\|ADS_{SA}\|$**	5.00E-03	0.86	0.05	0.10	7.52E-04	0.98

[a] significant at $P_{FDR}<0.05$; ADS_G=Average deviation score-global; ADS_{SA}=average deviation score-surface area; ADS_{SV}=average deviation score-subcortical volume; CHR-P = clinical high-risk for psychosis; ASD values preceded by P denoted sums of positive z-scores, ASD values preceded by N denote sums of negative z-scores and |ADS| denotes the sum of the absolute values of the z-score

© 2023 American Medical Association. All rights reserved.

eTable 17. Percentage of CHR-P With Infra- or Supranormal Normative Regional *z* Score According to Conversion Status

		CHR-PC (N = 157)		CHR-PNC (N = 940)	
**Region**	**Hemi**	**infranormal z-scores (%)**	**supranormal z-scores (%)**	**infranormal z-scores (%)**	**supranormal z-scores (%)**
**Subcortical Volume**					
nucleus accumbens	L	5.73	0.00	4.89	0.53
	R	8.28	0.00	6.17	0.11
amygdala	L	4.46	1.27	3.72	0.53
	R	5.10	3.82	3.19	1.70
caudate	L	8.92	0.64	5.96	0.53
	R	5.73	1.27	5.11	1.17
hippocampus	L	7.64	1.91	4.57	0.53
	R	5.73	1.91	3.83	0.53
pallidum	L	7.64	0.00	4.68	0.43
	R	10.19	0.00	5.21	1.28
putamen	L	8.28	0.64	10.00	0.32
	R	11.46	0.00	11.70	0.21
thalamus	L	9.55	0.64	8.51	0.96
	R	12.74	0.64	8.09	0.32
**Cortical Thickness**					
banks superior temporal sulcus	L	4.46	0.64	2.23	1.38
	R	4.46	1.91	3.19	1.81
caudal anterior cingulate	L	4.46	3.18	2.02	3.30
	R	3.18	3.82	3.51	4.57
caudal middle frontal	L	1.91	0.00	2.45	1.17
	R	1.91	1.91	3.30	1.70
cuneus	L	1.91	3.82	1.70	2.02
	R	3.82	0.64	1.81	2.98
entorhinal cortex	L	3.82	4.46	2.34	4.57
	R	2.55	1.91	2.45	4.26
fusiform gyrus	L	0.64	0.00	1.60	3.51
	R	2.55	1.91	1.70	3.51
inferior parietal	L	0.64	1.91	1.60	2.66
	R	3.18	1.91	1.81	1.81
inferior temporal	L	0.64	4.46	1.28	3.62
	R[a]	5.10	3.82	1.28	4.47
isthmus cingulate	L	5.10	1.27	5.11	1.91
	R	5.10	3.18	4.79	1.38
lateral occipital	L	0.64	1.91	0.64	4.57
	R	0.00	5.10	0.64	4.57
lateral orbitofrontal	L	1.91	1.91	1.49	2.02
	R	0.00	2.55	1.81	2.13

© 2023 American Medical Association. All rights reserved.

		CHR-PC (N = 157)		CHR-PNC (N = 940)	
**Region**	**Hemi**	**infranormal z-scores (%)**	**supranormal z-scores (%)**	**infranormal z-scores (%)**	**supranormal z-scores (%)**
lingual gyrus	L	5.10	2.55	3.19	1.81
	R	3.82	1.91	2.23	1.81
medial orbitofrontal	L	1.27	6.37	2.02	2.87
	R	1.27	3.82	1.49	2.87
middle temporal	L	1.27	2.55	2.23	2.13
	R	1.91	1.27	2.23	1.60
parahippocampal	L	3.18	0.64	2.45	2.02
	R	2.55	3.18	1.91	2.98
paracentral	L	2.55	1.27	1.49	2.23
	R	3.82	1.27	1.60	1.17
pars opercularis	L	3.82	0.64	3.83	1.28
	R	3.82	0.64	3.94	0.96
pars orbitalis	L	3.18	1.91	2.66	1.70
	R	0.64	1.27	1.60	2.66
pars triangularis	L	6.37	2.55	2.98	2.02
	R	3.18	0.64	3.83	1.28
pericalcarine	L	4.46	4.46	4.15	3.51
	R	1.91	5.73	2.77	4.47
postcentral	L	1.91	0.64	0.74	2.55
	R	1.27	0.64	1.28	1.60
posterior cingulate	L	3.18	1.91	5.85	2.23
	R	4.46	0.64	4.26	1.70
precentral	L	1.91	1.91	3.09	0.85
	R	1.27	0.00	2.87	0.74
precuneus	L	1.27	1.27	1.70	2.34
	R	1.27	3.18	2.66	1.49
rostral anterior cingulate	L	1.91	2.55	3.83	2.87
	R	4.46	2.55	3.19	2.77
rostral middle frontal	L	1.27	2.55	1.60	1.81
	R	1.27	3.18	1.49	2.13
superior frontal	L	1.27	2.55	3.94	1.60
	R	2.55	0.64	3.19	1.70
superior parietal	L	0.64	3.82	0.53	2.55
	R	0.64	5.10	0.74	2.13
superior temporal	L	4.46	0.64	2.87	0.74
	R	4.46	0.00	3.40	1.28
supramarginal gyrus	L	4.46	1.91	2.13	0.96
	R	3.82	1.91	4.36	1.38
frontal pole	L	3.82	4.46	2.34	3.72
	R	3.18	6.37	2.34	3.40

© 2023 American Medical Association. All rights reserved.

Region	Hemi	CHR-PC (N = 157)		CHR-PNC (N = 940)	
		infranormal z-scores (%)	supranormal z-scores (%)	infranormal z-scores (%)	supranormal z-scores (%)
temporal pole	L	1.27	2.55	0.85	2.66
	R	4.46	1.91	2.45	1.91
transverse temporal	L	1.27	0.64	3.09	1.49
	R	2.55	1.27	1.38	1.60
insula	L	4.46	0.64	4.15	0.64
	R	1.27	0.64	4.36	0.43
**Surface Area**					
banks superior temporal sulcus	L	1.91	1.27	2.55	1.91
	R[e]	7.01	2.55	1.38	2.23
caudal anterior cingulate	L	0.00	1.27	0.11	3.62
	R	0.64	3.18	0.43	3.19
caudal middle frontal	L	2.55	2.55	1.49	2.55
	R	0.64	2.55	1.28	3.09
cuneus	L	3.82	1.91	2.66	2.77
	R	1.27	1.91	1.38	3.09
entorhinal cortex	L	4.46	6.37	2.45	5.11
	R	0.00	7.01	1.60	4.68
fusiform gyrus	L	4.46	3.82	2.45	1.91
	R	1.91	1.91	2.45	1.60
inferior parietal	L	1.27	3.18	3.51	1.70
	R	2.55	0.64	3.09	3.19
inferior temporal	L	1.91	1.27	2.45	2.45
	R	1.91	3.18	2.77	1.91
isthmus cingulate	L	1.27	1.27	0.74	3.72
	R	1.27	3.18	0.85	4.26
lateral occipital	L	1.91	3.82	2.13	3.09
	R	3.18	2.55	1.38	2.34
lateral orbitofrontal	L	3.82	1.91	2.55	2.55
	R	1.91	3.82	2.66	3.62
lingual gyrus	L	1.91	4.46	3.19	1.60
	R	3.18	1.27	2.55	3.30
medial orbitofrontal	L	0.64	2.55	2.13	2.98
	R	4.46	3.82	2.23	2.45
middle temporal	L	5.73	1.91	3.09	3.30
	R	3.18	1.27	2.66	2.87
parahippocampal	L	0.64	3.18	2.66	1.60
	R	5.73	2.55	3.30	2.98
paracentral	L	1.27	3.82	0.96	3.19
	R	0.64	1.27	0.85	3.51
pars opercularis	L	1.27	3.82	1.70	2.45
	R	1.27	2.55	0.96	3.19

© 2023 American Medical Association. All rights reserved.

		CHR-PC (N = 157)		CHR-PNC (N = 940)	
Region	Hemi	infranormal z-scores (%)	supranormal z-scores (%)	infranormal z-scores (%)	supranormal z-scores (%)
pars orbitalis	L	0.64	3.82	2.77	2.98
	R	1.27	1.91	1.38	2.34
pars triangularis	L	1.27	4.46	3.19	1.28
	R	1.91	1.27	1.81	2.02
pericalcarine	L	0.64	0.64	1.81	3.30
	R	3.18	1.27	2.23	2.13
postcentral	L	1.27	6.37	0.96	5.53
	R	2.55	3.18	1.70	3.40
posterior cingulate	L	1.91	3.82	1.38	2.66
	R	0.00	3.82	1.38	2.55
precentral	L	1.91	3.82	1.28	4.47
	R	0.64	6.37	0.43	3.62
precuneus	L	3.82	5.73	2.02	1.81
	R	2.55	2.55	2.02	3.19
rostral anterior cingulate	L	0.64	3.18	1.60	3.62
	R	0.00	1.27	0.74	2.55
rostral middle frontal	L	2.55	3.18	2.55	2.87
	R	3.18	3.18	2.23	3.19
superior frontal	L	5.10	1.27	3.30	3.72
	R	0.64	2.55	2.66	2.13
superior parietal	L	1.91	1.91	1.81	2.13
	R	1.91	3.18	2.34	2.45
superior temporal	L	0.64	3.82	2.77	3.40
	R	2.55	1.91	1.60	3.40
supramarginal gyrus	L	3.18	1.27	2.34	4.47
	R	0.00	1.27	2.13	2.13
frontal pole	L	1.91	0.64	1.81	1.28
	R	1.27	2.55	1.28	1.17
temporal pole	L	0.64	1.91	0.85	2.13
	R	0.64	3.82	1.06	2.66
transverse temporal	L	1.91	2.55	0.85	4.26
	R	0.00	4.46	0.74	2.45
insula	L	1.27	3.82	1.49	6.70
	R	1.27	7.64	0.96	6.91
Average Deviation Scores					
ADS_G[a,c]		5.73	1.91	3.51	2.13
ADS_{SV}[a]		5.10	3.18	2.87	2.34
ADS_{CT}		4.46	3.18	2.98	1.91
ADS_{SA}[c]		3.18	2.55	2.98	3.19

[a] significant two-proportion z-tests difference in infranormal z-scores between CHR-PC and healthy individuals at $P_{FDR} < 0.05$; [b] significant two-proportion z-tests difference in supranormal z-scores between CHR-PC and healthy individuals at $P_{FDR} < 0.05$; [c] significant two-proportion z-tests

© 2023 American Medical Association. All rights reserved.

Region	Hemi	CHR-PC (N = 157)		CHR-PNC (N = 940)	
		infranormal z-scores (%)	supranormal z-scores (%)	infranormal z-scores (%)	supranormal z-scores (%)
difference in infranormal z-scores between CHR-PNC and healthy individuals at $P_{FDR} < 0.05$; [d] significant two-proportion z-tests difference in supranormal z-scores between CHR-PNC and healthy individuals at $P_{FDR} < 0.05$; [e] significant two-proportion z-tests difference in infranormal z-scores between CHR-PC and CHR-PNC at $P_{FDR} < 0.05$; [f] significant two-proportion z-tests difference in supranormal z-scores between CHR-PC and CHR-PNC at $P_{FDR} < 0.05$; ASD_{CT}=average deviation score-cortical thickness; ADS_{G}=Average deviation score-global; ADS_{SA}=average deviation score-surface area; ADS_{SV}=average deviation score-subcortical volume; CHR-PC = clinical high-risk for psychosis individuals that converted to a psychotic disorder; CHR-PNC = clinical high-risk individuals that did not convert to a psychotic disorder; Hemi = hemisphere; L = left; R = right.					

© 2023 American Medical Association. All rights reserved.

eTable 18. Associations Between Regional *z* Scores and Average Deviation Scores With Positive Symptoms and IQ Based on Conversion Status

	Hemi	All CHR-P N=1340	CHR-PC N=157	CHR-PNC N=940	All CHR-P N=1340	CHR-PC N=157	CHR-PNC N=940
		***β* Estimate for Positive Symptoms**			***β* Estimate for IQ**		
**Subcortical volumes**							
nucleus accumbens	L	1.53E-03	-0.12	0.03	0.02	0.12	-0.01
	R	-0.02	0.01	-0.03	0.01	0.02	0.02
amygdala	L	-0.01	-0.14	0.02	0.03	0.09	0.03
	R	1.81E-03	-0.13	0.02	0.02	0.05	0.02
caudate	L	-0.02	-0.13	-0.02	0.11[a]	0.23	0.09
	R	-0.03	-0.14	-0.05	0.10	0.20	0.09
hippocampus	L	-0.04	-0.12	-0.02	-0.01	-0.01	0.01
	R	-0.03	-0.16	-0.01	-0.01	3.45E-03	0.02
pallidum	L	-0.01	-0.04	-0.03	0.03	0.17	0.03
	R	-0.02	-0.02	-0.04	-0.01	0.07	-0.01
putamen	L	0.01	-0.07	0.01	-0.02	0.10	-0.05
	R	-0.01	-0.06	-0.01	1.92E-03	0.09	-0.02
thalamus	L	-0.03	-0.07	-0.02	0.07	0.20	0.06
	R	-0.02	-0.11	-0.03	0.06	0.26	0.03
**Cortical Thickness**							
banks superior temporal sulcus	L	0.01	0.04	0.01	0.01	-0.03	4.25E-03
	R	0.01	-0.11	0.05	-0.03	-0.16	4.34E-04
caudal anterior cingulate	L	-0.02	-0.01	-0.01	-0.01	-0.25	0.02
	R	-0.01	0.13	-0.02	0.03	0.01	0.04

© 2023 American Medical Association. All rights reserved.

	Hemi	All CHR-P N=1340	CHR-PC N=157	CHR-PNC N=940	All CHR-P N=1340	CHR-PC N=157	CHR-PNC N=940
		β Estimate for Positive Symptoms			*β* Estimate for IQ		
caudal middle frontal	L	-0.04	-0.07	-0.05	0.05	0.17	0.05
	R	0.01	-0.09	0.02	0.05	0.27	0.01
cuneus	L	-0.03	-0.04	-0.02	0.06	0.09	0.04
	R	0.01	1.57E-03	0.03	0.04	0.03	0.03
entorhinal cortex	L	-0.02	-0.01	-0.03	0.05	-0.04	0.03
	R	0.03	-0.05	0.05	0.04	-4.68E-03	0.04
fusiform gyrus	L	-0.04	-0.06	-0.02	0.03	0.04	0.01
	R	-0.02	0.03	4.92E-03	-3.01E-03	-4.77E-03	-0.04
inferior parietal	L	0.01	-0.02	0.01	-0.06	-0.06	-0.09
	R	0.01	-0.02	1.98E-03	-0.07	-0.07	-0.06
inferior temporal	L	-0.02	-0.01	-0.02	-3.98E-03	-0.09	-0.02
	R	-0.03	-0.02	-0.02	0.03	-0.06	0.02
isthmus cingulate	L	-5.49E-05	0.09	0.01	-0.02	0.04	-0.02
	R	-0.03	0.06	-0.04	0.03	0.16	0.01
lateral occipital	L	-0.07	-0.02	-0.08	-0.06	-3.10E-04	-0.05
	R	-0.04	0.09	-0.07	-0.02	-0.03	-0.04
lateral orbitofrontal	L	-0.03	-0.03	3.23E-03	-0.02	-0.19	-0.03
	R	-3.76E-03	0.05	0.01	0.03	-0.11	0.03
lingual gyrus	L	0.02	0.05	0.02	0.08	0.03	0.09
	R	-0.01	0.02	2.73E-03	0.05	0.08	0.05
medial orbitofrontal	L	1.69E-03	0.10	-3.03E-03	-0.04	-0.07	-0.06

© 2023 American Medical Association. All rights reserved.

	Hemi	All CHR-P N=1340	CHR-PC N=157	CHR-PNC N=940	All CHR-P N=1340	CHR-PC N=157	CHR-PNC N=940
		**β Estimate for Positive Symptoms**			**β Estimate for IQ**		
	R	0.02	0.18	3.54E-03	-0.02	-0.28	-2.61E-03
middle temporal	L	-3.07E-03	-0.07	8.68E-04	-0.01	-0.17	-0.01
	R	0.02	0.04	0.03	-0.04	-0.05	-0.05
parahippocampal	L	0.01	0.02	1.47E-03	0.04	0.02	0.05
	R	-1.02E-03	-0.07	0.01	-0.01	0.01	-0.02
paracentral	L	-0.01	-0.01	-0.02	-0.01	-0.02	-0.01
	R	0.04	0.07	0.03	-0.05	-0.19	-0.03
pars opercularis	L	0.02	0.17	0.01	3.03E-03	-0.05	0.05
	R	-0.01	0.05	-0.01	0.08	0.03	0.10
pars orbitalis	L	-0.01	0.05	-0.01	-0.02	-0.03	-0.01
	R	-0.03	-0.02	-0.01	-0.03	0.11	-0.07
pars triangularis	L	0.02	0.04	1.07E-03	0.02	0.10	0.02
	R	0.04	0.02	0.06	-0.03	0.03	-0.02
pericalcarine	L	0.01	-0.01	0.01	0.03	0.11	0.02
	R	0.01	-0.05	2.41E-03	0.02	0.09	0.01
postcentral	L	-0.02	-0.03	-0.02	0.05	0.11	0.04
	R	0.06	0.09	0.04	0.05	0.09	0.05
posterior cingulate	L	0.04	0.01	0.07	-0.01	-0.05	0.05
	R	-0.02	0.03	-0.02	-0.02	-0.22	0.01
precentral	L	-0.01	3.60E-03	-0.03	0.05	-0.02	0.08
	R	0.01	0.12	-0.02	0.02	-0.02	0.06

© 2023 American Medical Association. All rights reserved.

	Hemi	All CHR-P N=1340	CHR-PC N=157	CHR-PNC N=940	All CHR-P N=1340	CHR-PC N=157	CHR-PNC N=940
		***β* Estimate for Positive Symptoms**			***β* Estimate for IQ**		
precuneus	L	0.02	0.05	0.01	0.06	0.04	0.04
	R	0.02	4.61E-03	0.01	-0.03	0.05	-0.04
rostral anterior cingulate	L	0.02	-0.08	0.04	-0.01	-0.11	-0.02
	R	3.15E-03	0.14	5.68E-05	-0.03	-0.13	-0.01
rostral middle frontal	L	-0.03	0.07	-0.05	-0.02	0.20	-0.07
	R	-0.01	0.03	-0.02	-0.05	0.03	-0.09
superior frontal	L	-0.01	0.10	-0.03	-0.02	-0.02	-0.02
	R	-0.01	-0.06	-1.61E-03	-0.01	0.05	-0.04
superior parietal	L	-0.01	-0.18	-0.02	-0.08	-0.06	-0.08
	R	0.05	0.05	0.01	-0.03	-4.99E-03	-0.01
superior temporal	L	-0.02	-0.11	0.03	0.03	0.01	0.03
	R	0.03	-0.09	0.08	0.04	0.10	0.05
supramarginal gyrus	L	0.04	0.01	0.04	0.03	0.03	0.05
	R	0.06	0.10	0.05	0.01	0.04	4.32E-03
frontal pole	L	0.01	0.08	0.01	-0.03	-0.16	-0.02
	R	-0.02	0.02	-0.01	0.04	0.14	-0.01
temporal pole	L	0.03	-0.08	0.06	2.99E-03	0.14	-0.03
	R	0.02	-0.06	0.05	0.04	0.22	0.02
transverse temporal	L	-0.01	0.02	0.01	-0.03	-0.05	-0.03
	R	0.03	-0.04	0.04	0.01	0.05	0.04
insula	L	-3.09E-04	0.13	-0.01	-0.02	-0.13	0.03

© 2023 American Medical Association. All rights reserved.

	Hemi	All CHR-P N=1340	CHR-PC N=157	CHR-PNC N=940	All CHR-P N=1340	CHR-PC N=157	CHR-PNC N=940
		**β Estimate for Positive Symptoms**			**β Estimate for IQ**		
	R	-2.04E-03	0.13	-0.01	0.07	-0.14	0.12
**Cortical Surface Area**							
banks superior temporal sulcus	L	0.02	-0.07	0.02	-0.01	0.05	0.01
	R	0.02	0.10	0.01	-0.07	9.42E-05	-0.04
caudal anterior cingulate	L	-0.01	-0.09	0.01	0.03	0.10	0.02
	R	-0.01	0.06	-0.03	0.05	0.05	0.04
caudal middle frontal	L	-0.06	6.91E-04	-0.07	0.04	0.06	0.03
	R	-3.80E-03	0.07	-0.03	0.01	-0.20	0.03
cuneus	L	-2.72E-03	-0.14	0.03	0.11[a]	0.21	0.10
	R	0.01	-0.04	0.02	0.02	0.12	-0.03
entorhinal cortex	L	1.22E-03	0.10	-0.04	0.02	0.19	0.01
	R	-0.01	0.06	-0.04	4.72E-03	0.14	-0.01
fusiform gyrus	L	0.03	0.03	0.04	0.09	0.19	0.08
	R	0.03	0.02	0.04	0.05	0.16	0.06
inferior parietal	L	0.05	0.16	0.05	-0.03	0.10	-0.03
	R	-0.01	0.05	-0.02	-0.01	0.17	2.00E-03
inferior temporal	L	0.02	-0.09	0.03	0.04	0.06	0.08
	R	-3.74E-03	-0.09	-0.01	0.07	-0.02	0.07
isthmus cingulate	L	-3.82E-03	0.05	-0.01	-0.04	0.03	-0.08
	R	0.03	0.06	0.03	-0.02	0.01	-0.02
lateral occipital	L	0.01	0.02	-2.75E-03	0.01	-0.02	0.03

© 2023 American Medical Association. All rights reserved.

	Hemi	All CHR-P N=1340	CHR-PC N=157	CHR-PNC N=940	All CHR-P N=1340	CHR-PC N=157	CHR-PNC N=940
		β Estimate for Positive Symptoms			β Estimate for IQ		
	R	-1.66E-03	0.02	-2.88E-03	0.06	0.12	0.05
lateral orbitofrontal	L	-6.00E-04	-0.10	0.01	4.52E-03	0.03	-0.02
	R	0.01	-0.10	0.02	0.02	0.09	-0.01
lingual gyrus	L	-0.01	0.04	-0.01	0.07	0.11	0.07
	R	-0.02	-0.02	-0.02	0.04	0.12	0.02
medial orbitofrontal	L	-0.02	-0.05	-0.02	0.04	-0.03	0.06
	R	-0.04	-0.21	-0.01	0.04	0.28	0.01
middle temporal	L	0.02	0.01	0.02	-0.01	0.04	0.02
	R	0.03	-0.03	0.05	0.02	0.02	0.05
parahippocampal	L	-0.03	-0.17	-0.01	0.02	0.16	0.03
	R	-0.05	-0.02	-0.05	4.24E-03	0.09	0.02
paracentral	L	0.03	0.18	0.02	-0.06	-0.12	-0.06
	R	-0.05	-0.03	-0.03	-0.08	-0.19	-0.05
pars opercularis	L	-0.01	-0.11	-0.01	0.03	0.06	-1.93E-03
	R	-2.56E-03	-0.04	-0.02	0.04	-0.05	0.04
pars orbitalis	L	-0.06	-0.04	-0.07	0.05	-0.04	0.06
	R	-0.05	0.04	-0.08	0.06	0.11	0.03
pars triangularis	L	-0.02	0.01	-0.02	0.03	0.09	0.02
	R	-0.02	-0.05	-0.02	0.01	-0.08	0.01
pericalcarine	L	0.01	-0.06	0.02	0.08	0.11	0.06
	R	3.51E-03	-0.14	0.03	0.02	0.14	-0.04

© 2023 American Medical Association. All rights reserved.

	Hemi	All CHR-P N=1340	CHR-PC N=157	CHR-PNC N=940	All CHR-P N=1340	CHR-PC N=157	CHR-PNC N=940
		**β Estimate for Positive Symptoms**			**β Estimate for IQ**		
postcentral	L	-0.03	0.02	-0.02	0.01	-0.01	3.05E-03
	R	-0.03	0.03	-0.02	-0.02	0.07	-0.04
posterior cingulate	L	-2.80E-03	0.06	-0.03	-0.01	-0.06	-0.03
	R	0.01	0.12	-0.02	0.01	0.10	0.01
precentral	L	-0.04	-0.12	-0.03	0.02	0.08	-0.02
	R	-0.01	-0.13	-0.01	-1.18E-03	-0.09	0.02
precuneus	L	0.01	-0.04	-0.01	-0.09	-0.02	-0.09
	R	-0.02	-0.07	-0.03	0.02	-0.04	0.05
rostral anterior cingulate	L	-0.04	-0.08	2.32E-03	0.09	0.28	0.07
	R	-0.04	-0.09	-0.04	8.64E-04	0.03	-0.02
rostral middle frontal	L	0.02	0.09	0.01	0.04	-0.11	0.07
	R	0.02	0.04	0.02	0.03	0.12	0.01
superior frontal	L	0.01	0.03	5.56E-05	-3.30E-04	-0.05	2.16E-03
	R	-0.03	-0.01	-0.03	-0.01	-0.18	0.02
superior parietal	L	-0.02	0.01	-0.02	-0.02	-0.09	-0.04
	R	-0.01	-0.05	-4.86E-03	-0.04	-0.04	-0.05
superior temporal	L	-0.03	-0.04	-0.03	-0.03	-0.05	-0.06
	R	0.03	0.10	0.04	-0.04	-0.14	-0.03
supramarginal gyrus	L	-0.03	0.11	-0.08	-0.03	-0.13	-0.02
	R	-0.03	0.13	-0.05	-0.03	-0.12	-0.03
frontal pole	L	-0.03	0.02	-0.07	-0.01	-0.13	0.03

© 2023 American Medical Association. All rights reserved.

	Hemi	All CHR-P N=1340	CHR-PC N=157	CHR-PNC N=940	All CHR-P N=1340	CHR-PC N=157	CHR-PNC N=940
		**β Estimate for Positive Symptoms**			**β Estimate for IQ**		
	R	0.01	-0.01	2.96E-03	-0.01	-0.16	-0.02
temporal pole	L	0.02	0.03	0.01	-0.01	0.13	-0.04
	R	4.22E-03	-0.08	-7.68E-04	-0.06	-0.15	-0.08
transverse temporal	L	-0.03	-0.05	-0.03	-0.03	-0.10	-0.05
	R	-0.01	0.04	-4.31E-03	-0.04	-0.26	-0.05
insula	L	-0.02	-0.08	-0.01	-0.01	0.10	-0.04
	R	-0.05	-0.16	-0.03	-0.04	0.07	-0.08
**Average Deviation Scores**							
ADS_G		-0.05	-0.07	-0.04	0.10[a]	0.21[a]	0.07
ADS_{SV}		-0.03	-0.15	-0.02	0.05	0.20[a]	0.04
ADS_{CT}		0.01	0.12	0.03	0.04	-0.03	0.04
ADS_{SA}		-0.08[a]	-0.12	-0.09[a]	0.08[a]	0.26[a]	0.04

[a] significant associations at $P_{FDR}<0.05$; ADS_G=Average deviation score-global; ASD_{CT}=average deviation score-cortical thickness; ADS_{SA}=average deviation score-surface area; ADS_{SV}=average deviation score-subcortical volume; CHR-P = clinical high-risk for psychosis; CHR-PC = clinical high-risk for psychosis converters; CHR-PNC = clinical high-risk for psychosis non-converters; L=left; R=right

© 2023 American Medical Association. All rights reserved.

eFigure 1. Flow Diagram for Study Sample Selection of Individuals at Clinical High-Risk for Psychosis (CHR-P) and Healthy Individuals (HI) From the Sample Available Through the ENIGMA CHR-P Working Group

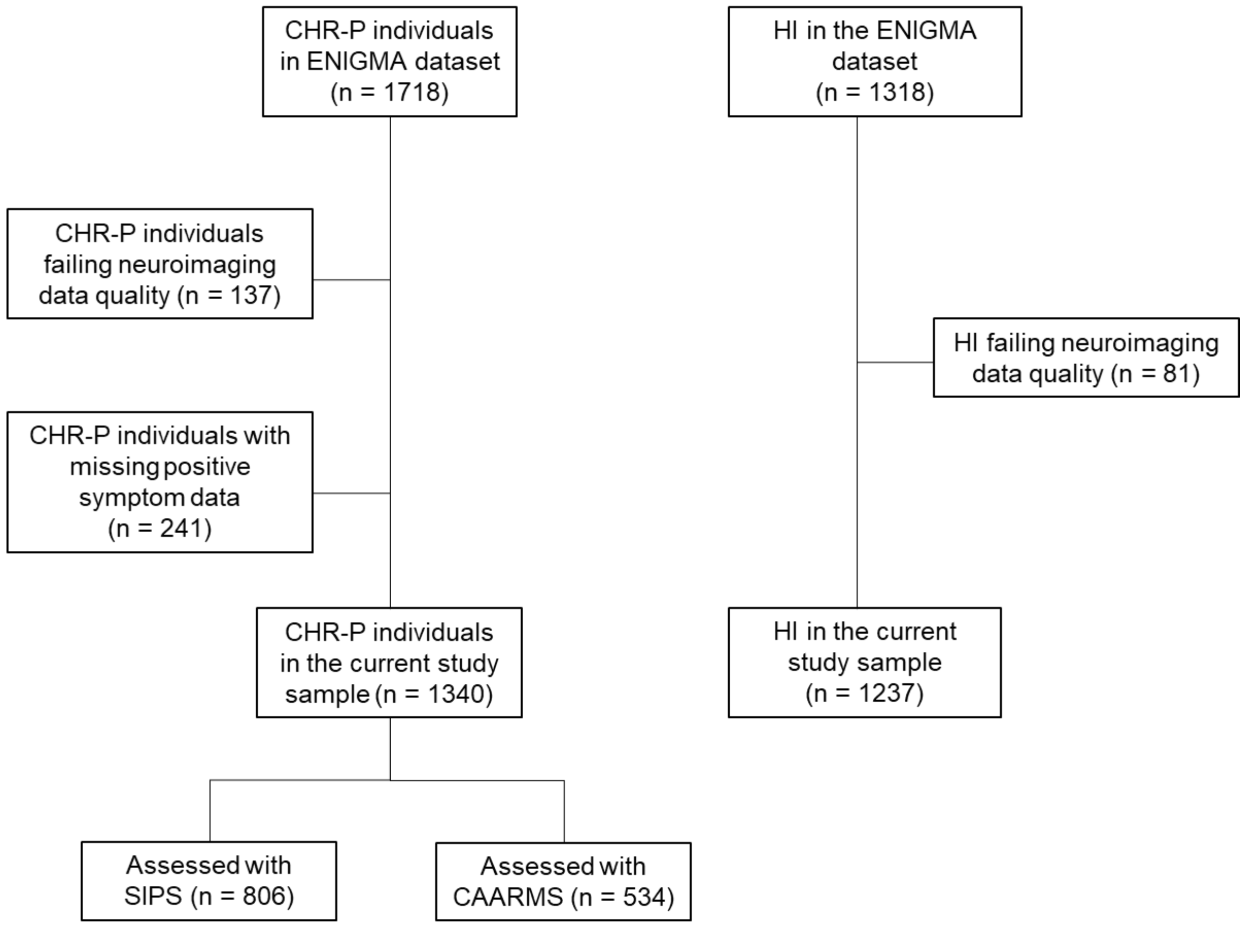

© 2023 American Medical Association. All rights reserved.

eFigure 2. Age Distribution in the Entire Sample and Within Each Site

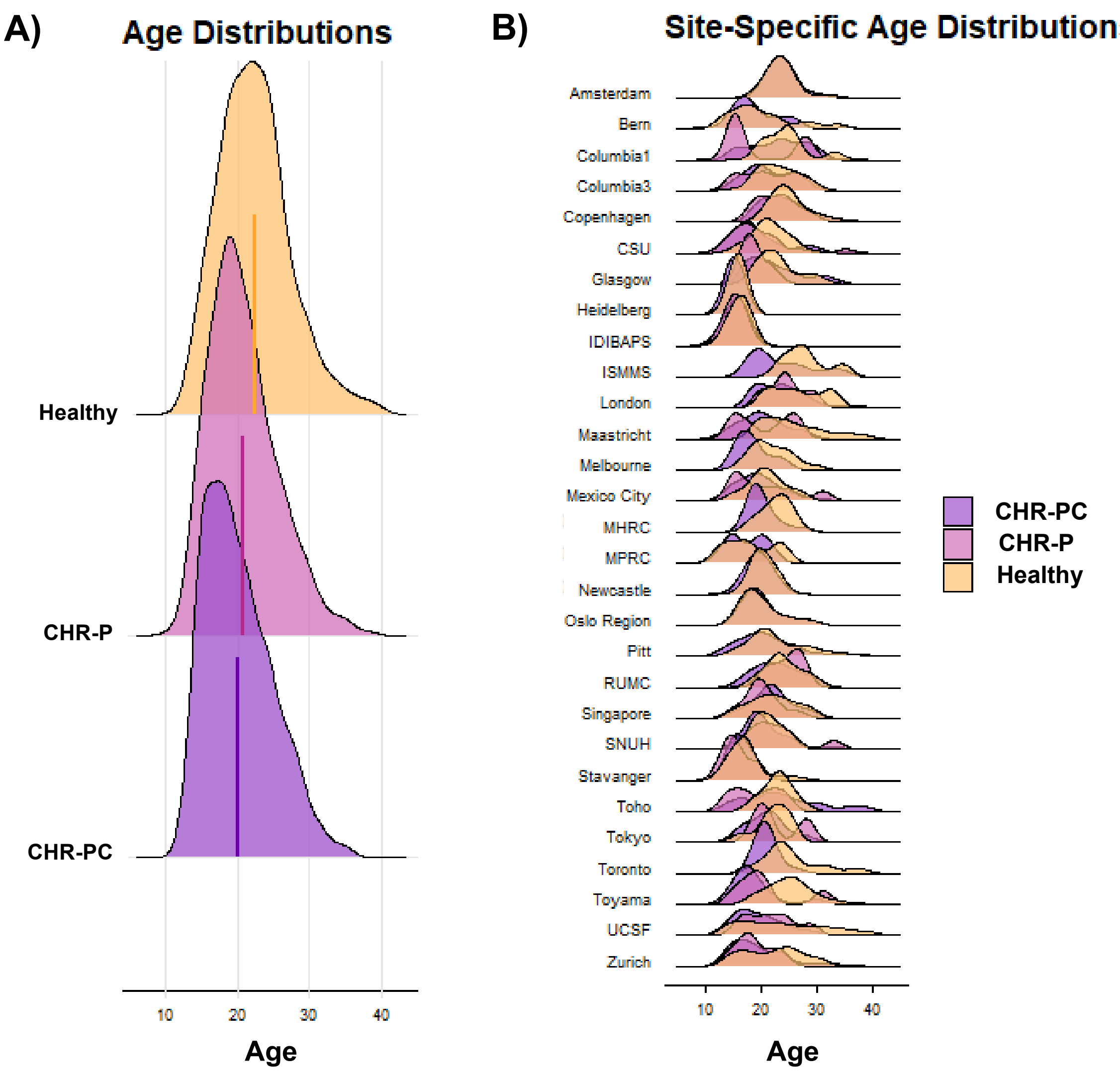

The figure illustrates the distribution of age in clinical high risk for psychosis (CHR-P), CHR-P converters (CHR-PC), and healthy individuals (Panel A) in the entire study sample and in each site separately (panel B).

© 2023 American Medical Association. All rights reserved.

eFigure 3. Computation of Regional Normative Deviation Scores (*z* Scores)

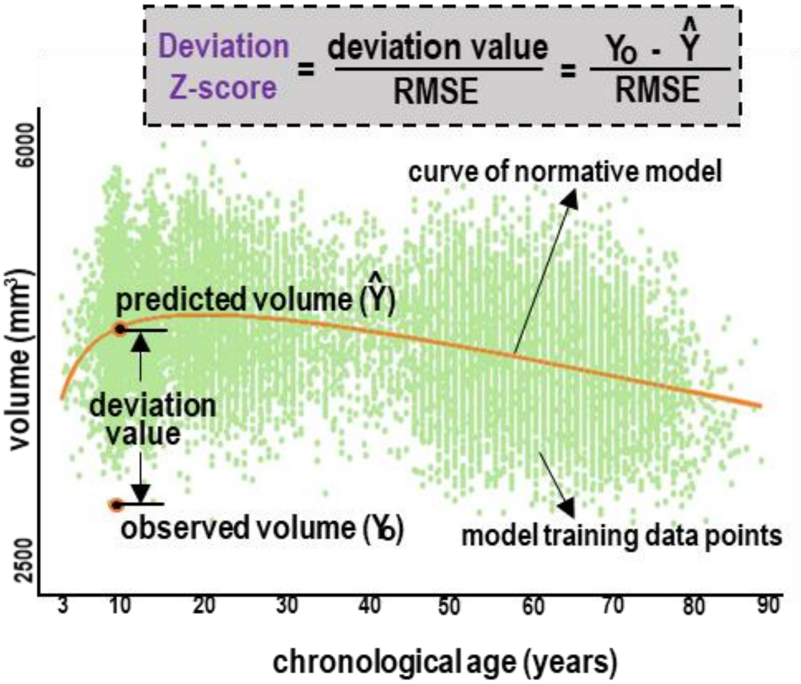

The figure illustrates the computation of the deviation score (z-score) using the right hippocampus as an example. The green dots represent the observed hippocampal volume, and the orange curve is the MFPR estimated mean.

© 2023 American Medical Association. All rights reserved.

eFigure 4. Distribution of Normative Regional *z* Scores for FreeSurfer-Derived Brain Morphometric Measures in CHR-P and Healthy Individuals in the Entire Study Sample

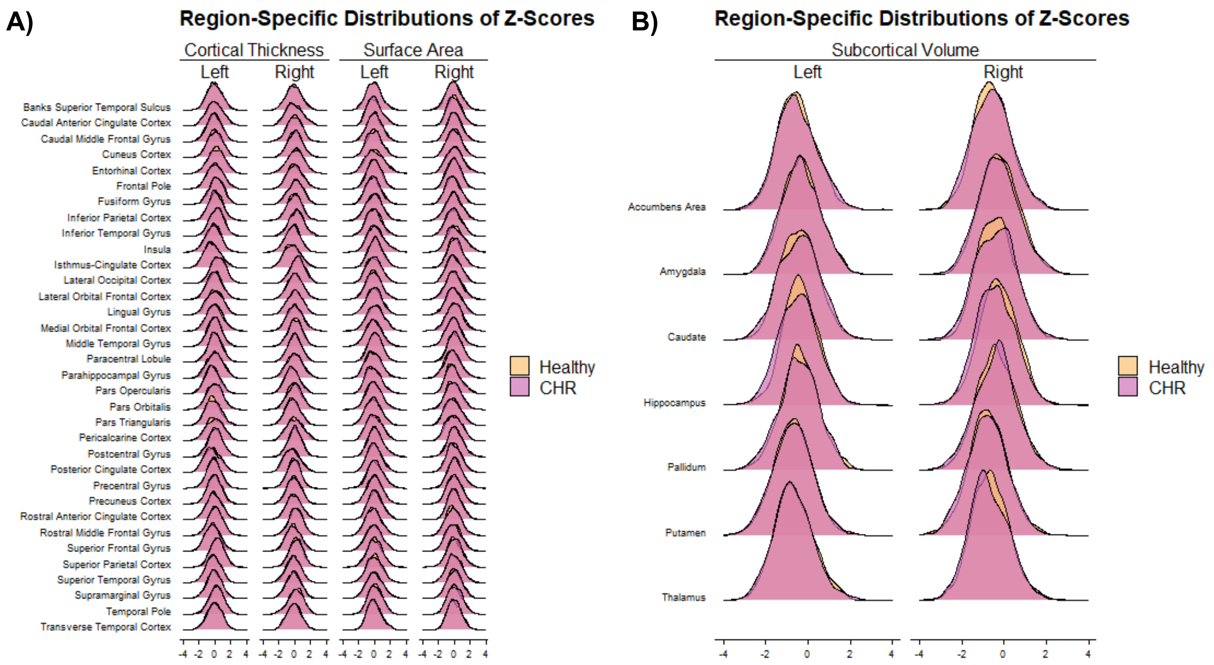

The figure illustrates the overlap of the distribution of deviation scores per region in clinical high-risk for psychosis (CHR-P) and healthy individuals for cortical thickness and surface area (panel A) and subcortical volumes (panel B).

© 2023 American Medical Association. All rights reserved.

eFigure 5. Distribution of Normative Regional *z* Scores and Average Deviation Scores of Freesurfer-Derived Brain Morphometric Measures in CHR-P and Healthy Individuals by Site

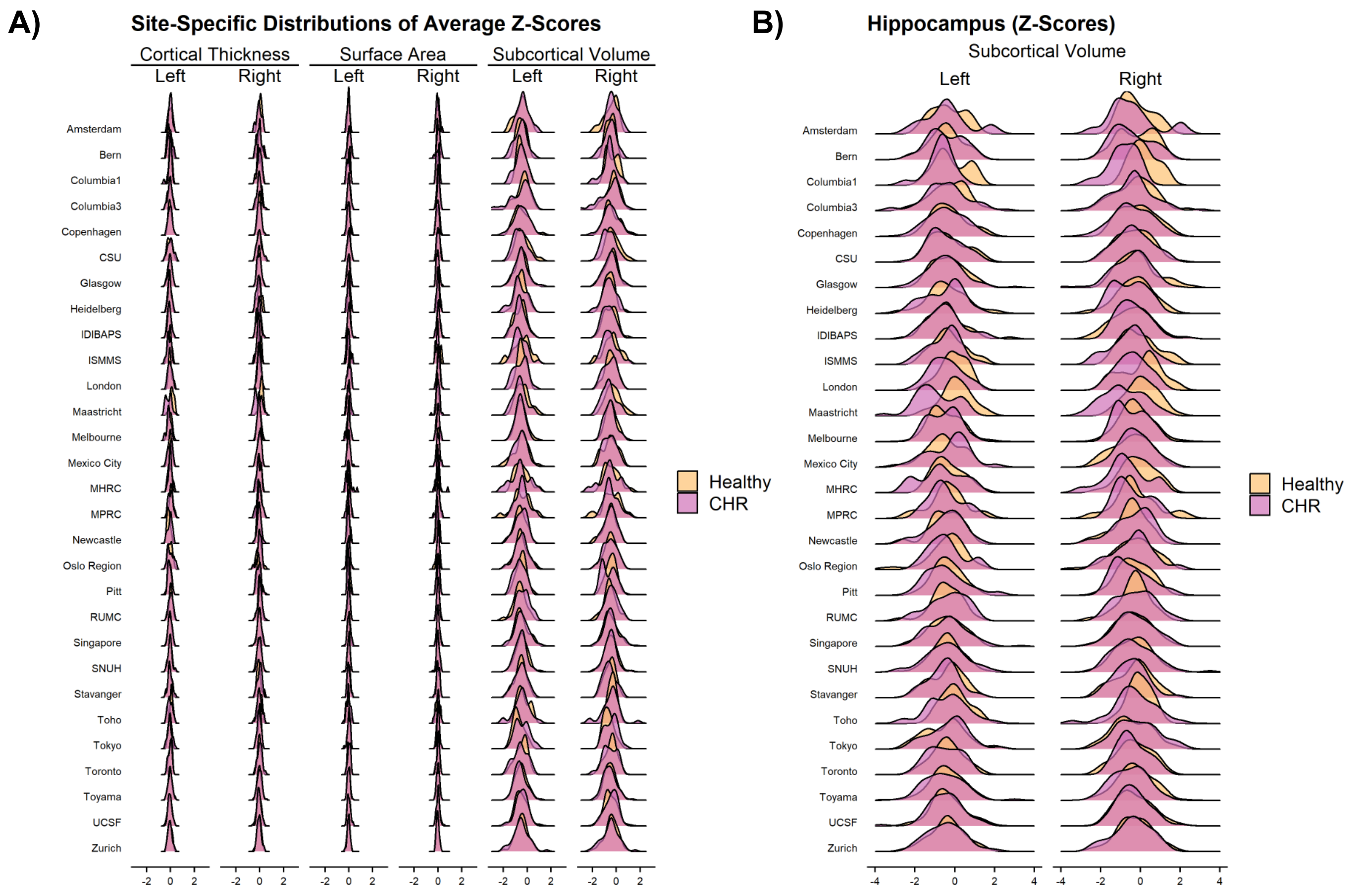

The figure illustrates the overlap of the distribution of deviation scores in clinical high risk for psychosis (CHR-P) and healthy individuals for average deviation scores and B) regional deviations in the left and right hippocampus as an example. Data for the remaining distributions of regional brain morphometric deviations are available upon request.

© 2023 American Medical Association. All rights reserved.

eFigure 6. Distribution of Normative Regional *z* Scores for Cortical Thickness Based on the Schaefer 400 Parcellation Distribution for the Left (Panel A) and the Right Hemisphere (Panel B)

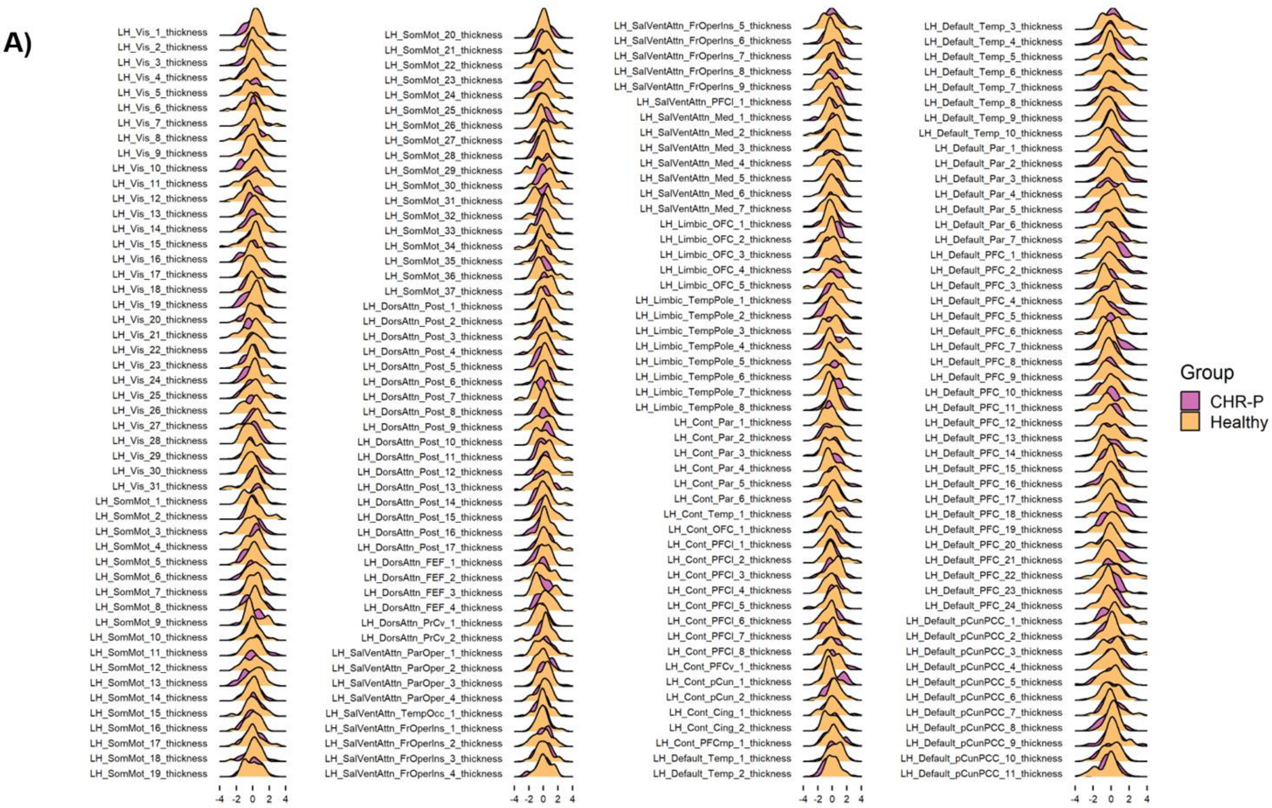

© 2023 American Medical Association. All rights reserved.

B)

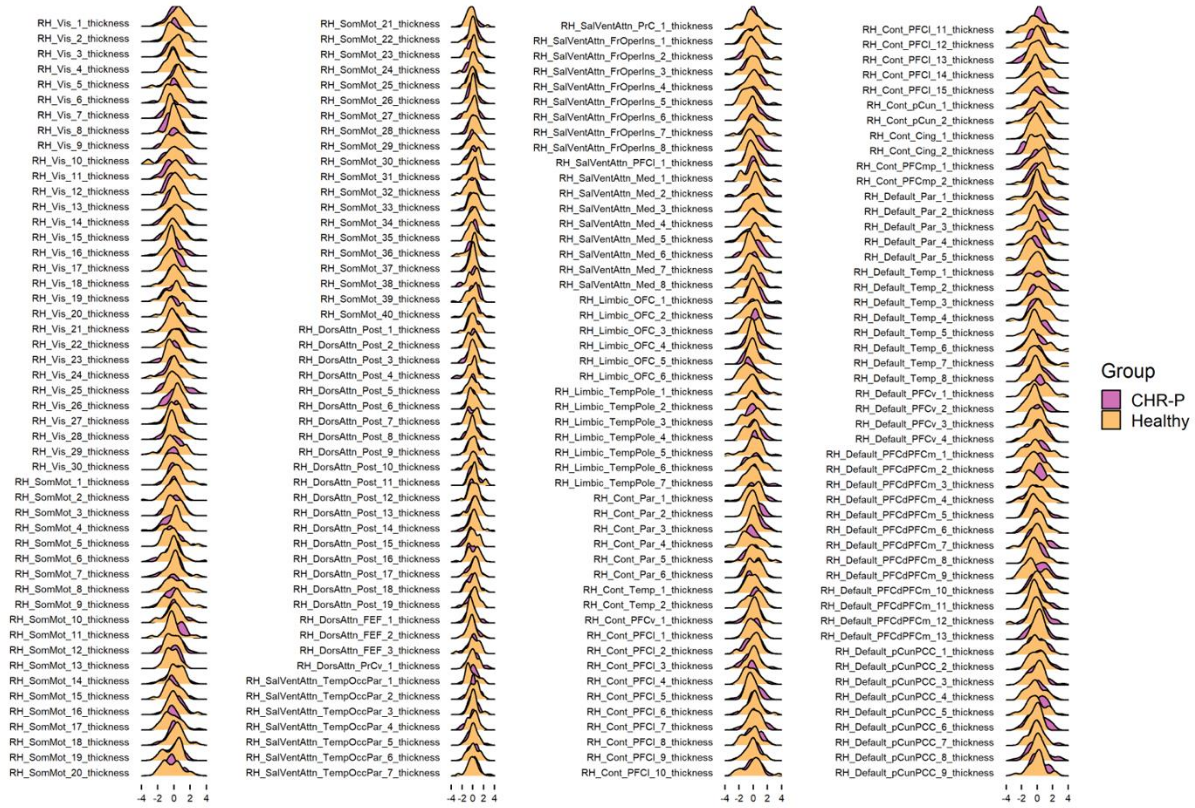

© 2023 American Medical Association. All rights reserved.

eFigure 7. Distribution of Normative Regional *z* Scores for Cortical Surface Area Based on the Schaefer 400 Parcellation for the Left (Panel A) and the Right Hemisphere (Panel B)

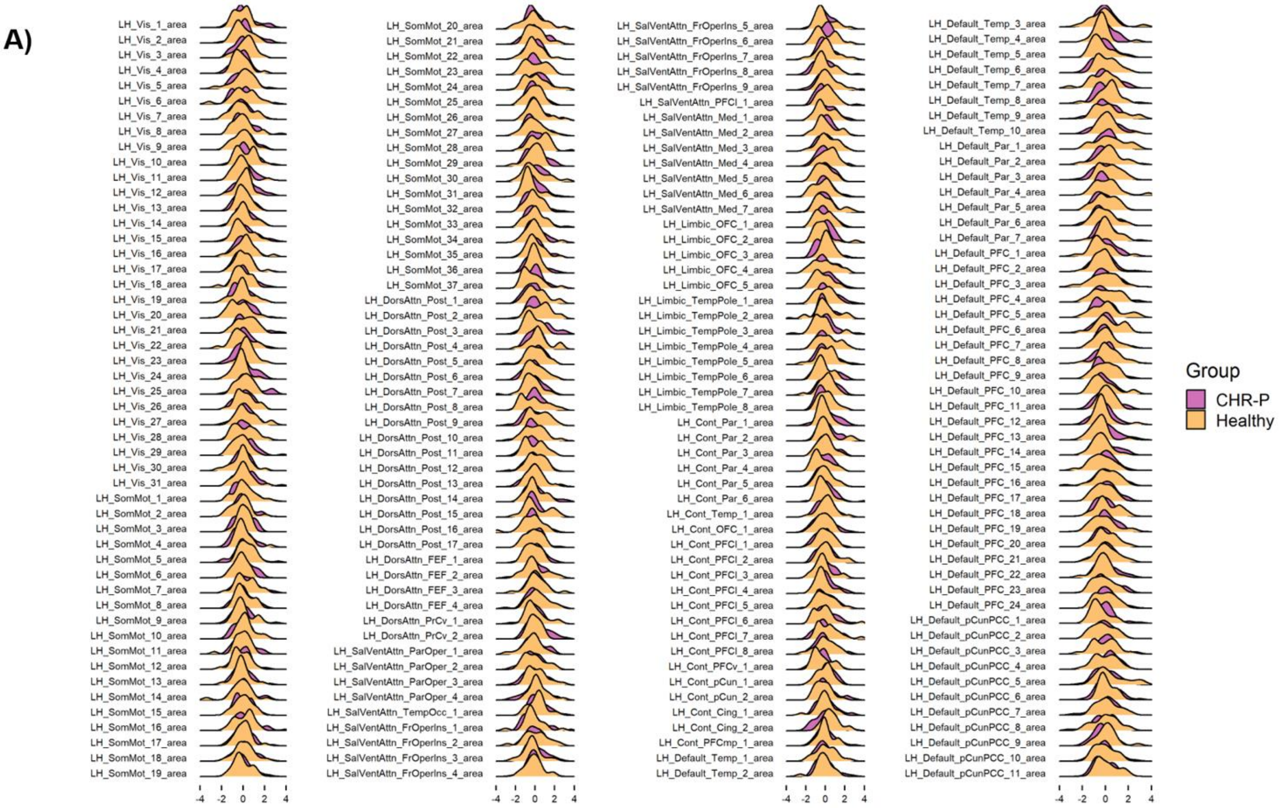

© 2023 American Medical Association. All rights reserved.

B)

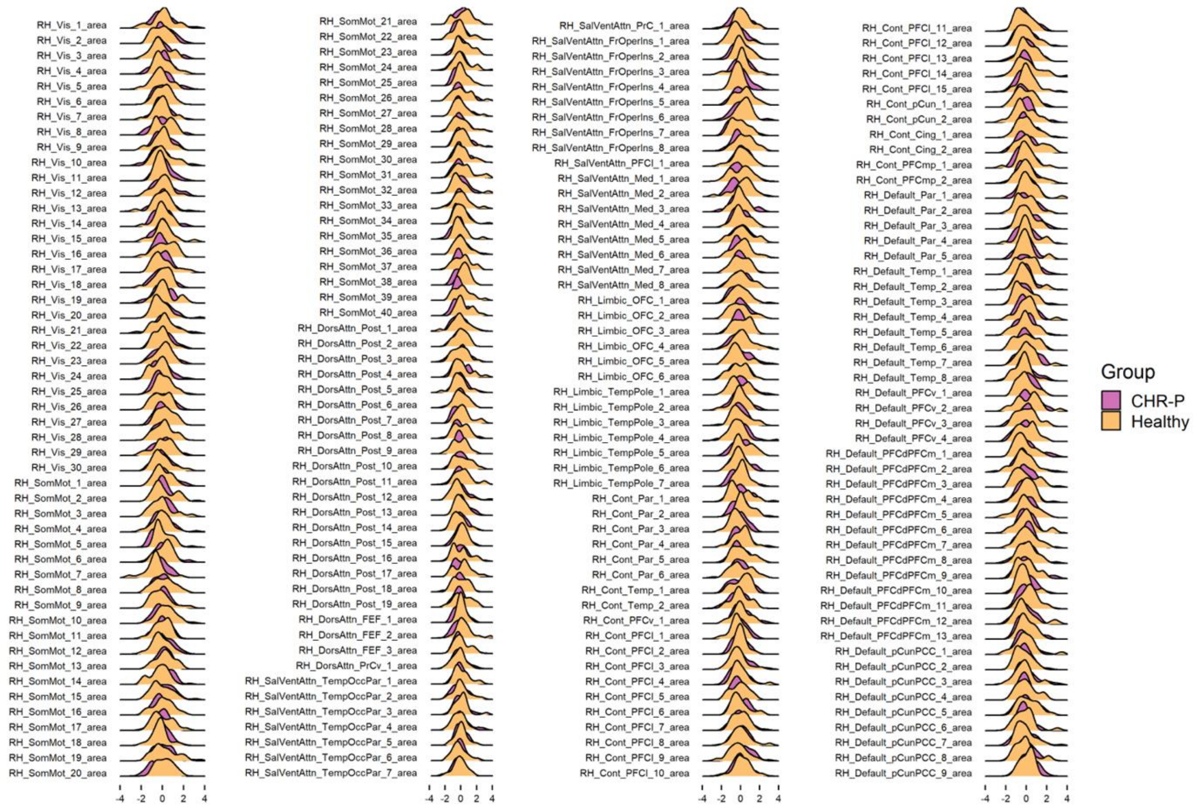

© 2023 American Medical Association. All rights reserved.

eFigure 8. Percentage of subjects with infra- or supranormal regional normative z-scores for subcortical volume

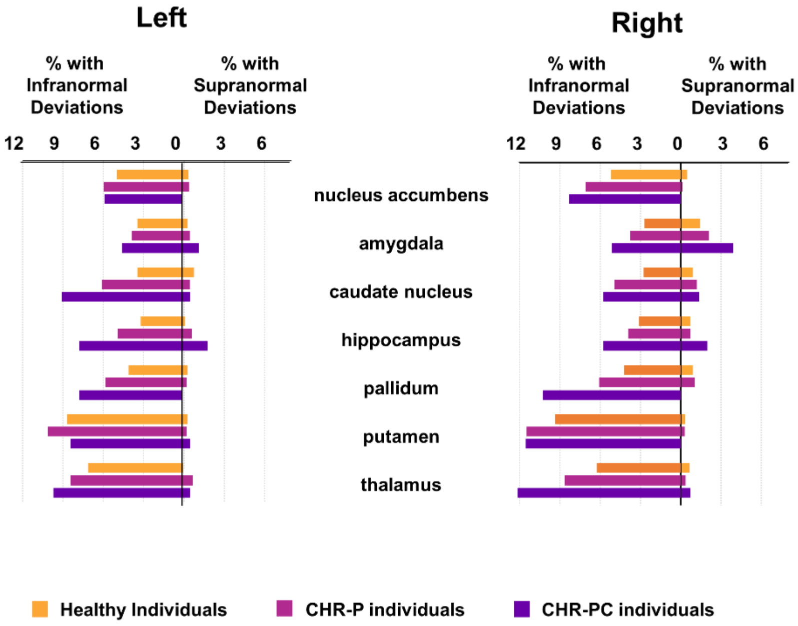

The proportion of healthy individuals, clinical high-risk for psychosis (CHR-P), and clinical high-risk for psychosis converters (CHR-PC) with infranormal (left panel) and supranormal deviations (right panel) are presented for each hemisphere for subcortical volume. Results for cortical thickness and surface area are presented in Figure 2 in the manuscript.

© 2023 American Medical Association. All rights reserved.

eFigure 9. Distributions of the Average Deviation Scores and Percentage of Subjects With Infra- or Supranormal Regional Normative *z* Scores

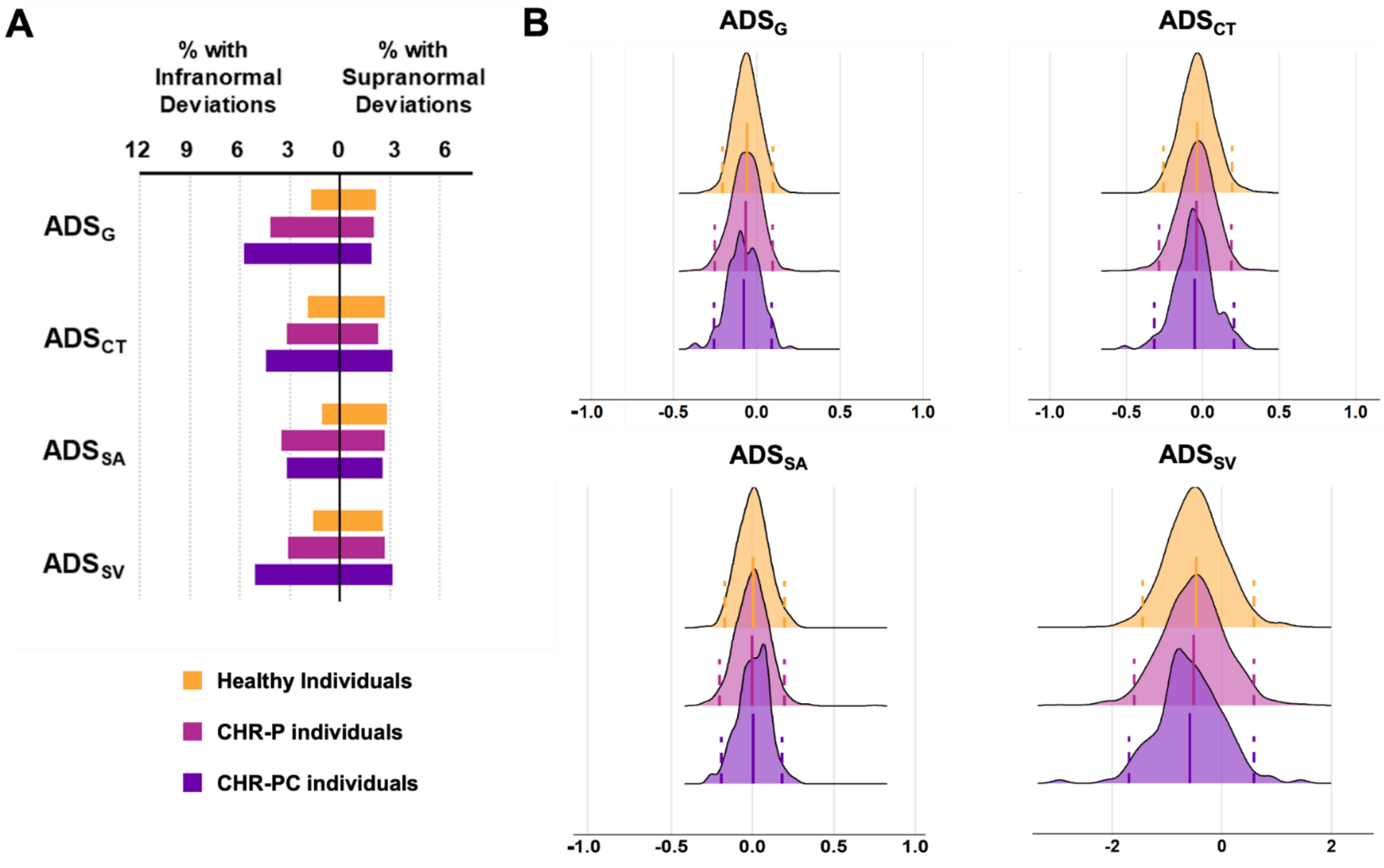

A) Percentage of healthy, clinical high-risk for psychosis (CHR-P), and clinical high-risk for psychosis converters (CHR-PC) with supra- or infranormal global average deviation score (ADSG), the average deviation score for cortical thickness (ADSCT), average deviation score for cortical surface area (ADSSA), and average deviation score for subcortical volumes (ADSSV). B) The distributions of the average deviation scores in CHR-P (magenta color), CHR-PC (purple color), and healthy individuals (yellow color) for ADSG, ADSCT, ADSSA, and ADSSV. The dotted lines represent the cutoffs for infranormal and supranormal values at z = |1.96|.

© 2023 American Medical Association. All rights reserved.

eFigure 10. Associations Between Regional and Average Deviation Scores and Clinical Measures in CHR-P Individuals

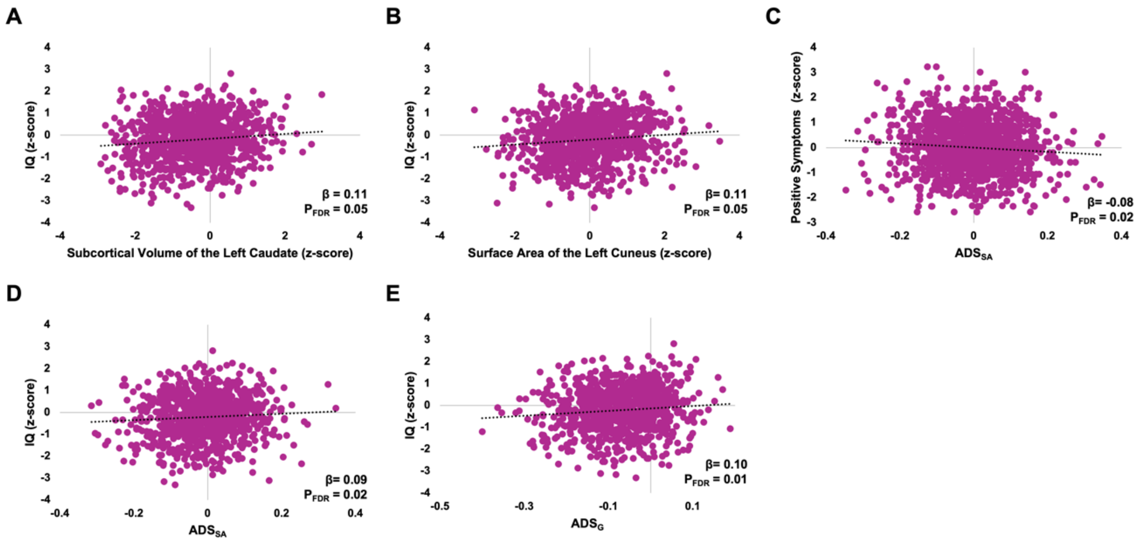

Panels A-B, The associations between IQ and regional normative z-scores of the left caudate volume (panel A) the surface area of the left cuneus (panel B) in individuals at clinical high-risk for psychosis (CHR-P). Panel C: The association between positive symptoms and cortical surface area average deviation score (ADS_{SA}) in CHR-P. Panels D-E: The association between IQ (panel D) and ADS_{SA} and global average deviation score (ADS_G) (panel E) in CHR-P individuals.

© 2023 American Medical Association. All rights reserved.

eFigure 11. Associations Between Average Deviation Scores With the Positive Symptoms and IQ Based on Medication Exposure and Removing 1 Site at a Time

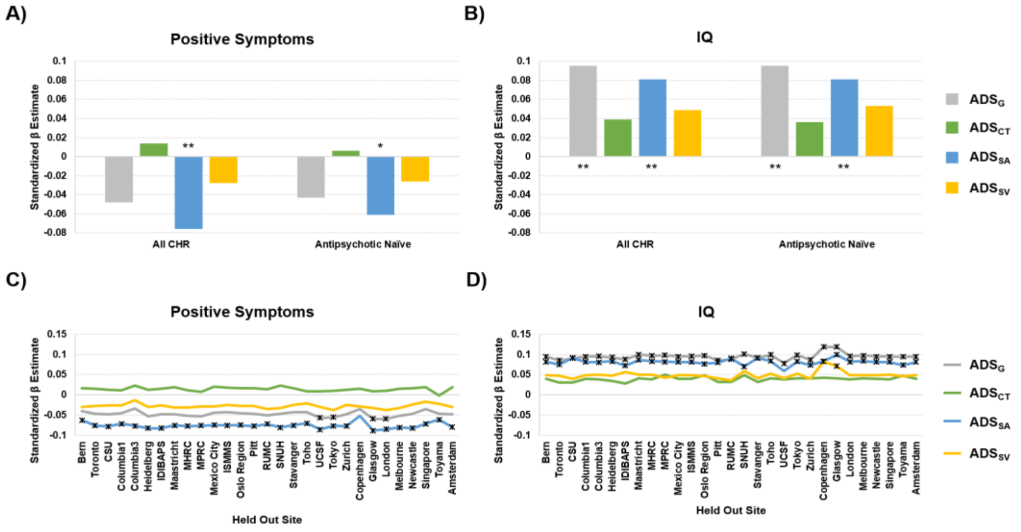

Standardized beta (β) estimates are presented in the y-axis; The effect of antipsychotic medication exposure is shown on the x-axis in panels A and B, the site left-out is shown on the x-axis in panels C and D; ADS_G=Average deviation score-global; ADS_{CT}=Average deviation score-cortical thickness; ADS_{SA}=Average deviation score-cortical surface area; ADS_{SV}= Average deviation score-subcortical volume; IQ = intelligence quotient, *significant at uncorrected P < 0.05; ** significant at P_{FDR} < 0.05.

© 2023 American Medical Association. All rights reserved.

eReferences

1. Miller TJ, McGlashan TH, Woods SW, et al. Symptom Assessment in Schizophrenic Prodromal States. *Psychiatr Q*. 1999;70(4):273-287. doi:10.1023/A:1022034115078

2. Miller TJ, McGlashan TH, Rosen JL, et al. Prodromal Assessment with the Structured Interview for Prodromal Syndromes and the Scale of Prodromal Symptoms: Predictive Validity, Interrater Reliability, and Training to Reliability. *Schizophr Bull.* 2003;29(4):703-715. doi:10.1093/oxfordjournals.schbul.a007040

3. Yung AR, Yung AR, Pan Yuen H, et al. Mapping the Onset of Psychosis: The Comprehensive Assessment of At-Risk Mental States. *Aust N Z J Psychiatry*. 2005;39(11-12):964-971. doi:10.1080/j.1440-1614.2005.01714.x

4. Royston, P., & Altman, D. G. Regression using fractional polynomials of continuous covariates: parsimonious parametric modelling. *Journal of the Royal Statistical Society: Series C (Applied Statistics)*. 1994;43(3):429-453. doi: 10.2307/2986270

5. Pomponio R, Erus G, Habes M, et al. Harmonization of large MRI datasets for the analysis of brain imaging patterns throughout the lifespan. *Neuroimage*. 2020;208:116450. doi:10.1016/j.neuroimage.2019.116450

6. Crawford JR, Garthwaite PH. Comparing patients' predicted test scores from a regression equation with their obtained scores: a significance test and point estimate of abnormality with accompanying confidence limits. *Neuropsychology*. 2006;20(3):259-271. doi:10.1037/0894-4105.20.3.259

7. Crawford JR, Garthwaite PH, Denham AK, Chelune GJ. Using regression equations built from summary data in the psychological assessment of the individual case: extension to multiple regression. *Psychol Assess*. 2012;24(4):801-814. doi:10.1037/a0027699

8. Potvin, O., et al. NOMIS: Quantifying morphometric deviation from normality over the lifetime in the adult human brain. bioRxiv. 2022. doi: 10.1101/2021.01.25.428063

9. Schaefer A, Kong R, Gordon EM, et al. Local-Global Parcellation of the Human Cerebral Cortex from Intrinsic Functional Connectivity MRI. Cereb Cortex. 2018;28(9):3095-3114. doi:10.1093/cercor/bhx179

10. Bryce NV, Flournoy JC, Guassi Moreira JF, et al. Brain parcellation selection: An overlooked decision point with meaningful effects on individual differences in resting-state functional connectivity. Neuroimage. 2021;243:118487. doi:10.1016/j.neuroimage.2021.118487

11. Valk SL, Xu T, Margulies DS, et al. Shaping brain structure: Genetic and phylogenetic axes of macroscale organization of cortical thickness. Sci Adv. 2020;6(39):eabb3417. Published 2020 Sep 25. doi:10.1126/sciadv.abb3417

12. Dinga R., et al. Normative modeling of neuroimaging data using generalized additive models of location scale and shape. bioRxiv. 2021. doi: 10.1101/2021.06.14.448106

13. Wolfers T, Doan NT, Kaufmann T, et al. Mapping the Heterogeneous Phenotype of Schizophrenia and Bipolar Disorder Using Normative Models. JAMA Psychiatry. 2018;75(11):1146-1155. doi:10.1001/jamapsychiatry.2018.2467

14. Sripada C, Angstadt M, Rutherford S, et al. Basic Units of Inter-Individual Variation in Resting State Connectomes. Sci Rep. 2019;9(1):1900. Published 2019 Feb 13. doi:10.1038/s41598-018-38406-5

15. Sripada C, Rutherford S, Angstadt M, et al. Prediction of neurocognition in youth from resting state fMRI. Mol Psychiatry. 2020;25(12):3413-3421. doi:10.1038/s41380-019-0481-6

© 2023 American Medical Association. All rights reserved.
