# Supplementary material for: Normative Modeling of Brain Morphometry in Clinical High Risk for Psychosis
Source: JAMA Psychiatry. 2023 Oct 11;81(1):77–88. doi: 10.1001/jamapsychiatry.2023.3850 (PMC10568447; doi:10.1001/jamapsychiatry.2023.3850)
Supplement: Supplement 2. — Nonauthor Collaborators. The ENIGMA Clinical High Risk for Psychosis Working Group Collaborators [file jamapsychiatry-e233850-s002.pdf]

Supplemental Online Content: Nonauthor Collaborators
*First name, last name, and suffix (if applicable) are required and will appear in PubMed.

*Group Name(s): ENIGMA Clinical High Risk for Psychosis Working Group

*First Name and Middle Initial(s)	*Last Name	*Suffix (eg, Jr, III)	Academic Degrees	Institution	Location (city, state/province, country)	Role or Contribution, eg, chair, principal investigator	Group (if more than 1 Group listed in the byline) and/or Subgroup (eg, Steering Committee)
Paul	Allen		PhD	Department of Psychology, University of Roehampton	London, UK		
				Department of Psychosis Studies, Institute of Psychiatry, Psychology and Neuroscience, King's College London	London, UK		
Helen	Baldwin		MSc	Department of Psychosis Studies, Institute of Psychiatry, Psychology and Neuroscience, King's College London	London, UK		
				NIHR Maudsley Biomedical Research Centre, South London and Maudsley NHS Foundation	London, UK		
Cali F	Bartholomeusz		PhD	Orygen	Melbourne, VIC, Australia		
				Centre for Youth Mental Health, University of Melbourne	Melbourne, VIC, Australia		
Michael WL	Chee		MBBS	Centre for Sleep and Cognition, Yong Loo Lin School of Medicine, National University of Singapore	Singapore		
				Centre for Translational Magnetic Resonance Research,Yong Loo Lin School of Medicine, National University of Singapore	Singapore		
Xiaogang	Chen		MD, PhD	National Clinical Research Center for Mental Disorders and Department of Psychiatry, The Second Xiangya Hospital of Central South University	Changsha, China		
				National Clinical Research Center for Geriatric Disorders, Xiangya Hospital, Central South University	Changsha, China		
Rebecca E	Cooper		BBmed(hons)	Melbourne Neuropsychiatry Centre, Department of Psychiatry, University of Melbourne & Melbourne Health	Melbourne, VIC, Australia		
Lieuwe	de Haan		MD, PhD	Department of Psychiatry, Amsterdam University Medical Centre	Amsterdam, The Netherlands		
				Arkin	Amsterdam, The Netherlands		

Supplemental Online Content: Nonauthor Collaborators
*First name, last name, and suffix (if applicable) are required and will appear in PubMed.

***First Name and Middle Initial(s)**	***Last Name**	***Suffix (eg, Jr, III)**	Academic Degrees	Institution	Location (city, state/province, country)	Role or Contribution, eg, chair, principal investigator	Group (if more than 1 Group listed in the byline) and/or Subgroup (eg, Steering Committee)
Holly K	Hamilton		PhD	Department of Psychiatry and Behavioral Sciences, University of California San Francisco	San Francisco, CA, USA		
				San Francisco Veterans Affairs Health Care System	San Francisco, CA, USA		
Ying	He		MD, PhD	National Clinical Research Center for Mental Disorders and Department of Psychiatry, The Second Xiangya Hospital of Central South University	Changsha, China		
Wenche ten Velden	Hegelstad		PhD	Faculty of Social Sciences, University of Stavanger	Stavanger, Norway		
				TIPS Centre for Clinical Research in Psychosis, Stavanger University Hospital	Stavanger, Norway		
Leslie E	Horton		PhD	Department of Psychiatry, University of Pittsburgh	Pittsburgh, PA, USA		
Daniela	Hubl		MD	Translational Research Center, University Hospital of Psychiatry and Psychotherapy, University of Bern	Bern, Switzerland		
Mallory J	Klaunig		PhD	Department of Psychology, University of Maryland	College Park, MD, USA		
Alex	Koppel		HBSc	Department of Pharmacology and Toxicology, University of Toronto	Toronto, Canada		
Yoo Bin	Kwak		BA	Department of Brain and Cognitive Sciences, Seoul National University College of Natural Sciences	Seoul, Republic of Korea		
Pablo	León-Ortiz		MD, PhD	Laboratory of Experimental Psychiatry, Instituto Nacional de Neurología y Neurocirugía	Mexico City, Mexico		
Rachel L	Loewy		PhD	Department of Psychiatry and Behavioral Sciences, University of California San Francisco	San Francisco, CA, USA		
Patrick	McGorry		MD, PhD	Centre for Youth Mental Health, University of Melbourne	Melbourne, VIC, Australia		
				Orygen	Melbourne, VIC, Australia		

Supplemental Online Content: Nonauthor Collaborators
*First name, last name, and suffix (if applicable) are required and will appear in PubMed.

***First Name and Middle Initial(s)**	***Last Name**	***Suffix (eg, Jr, III)**	Academic Degrees	Institution	Location (city, state/province, country)	Role or Contribution, eg, chair, principal investigator	Group (if more than 1 Group listed in the byline) and/or Subgroup (eg, Steering Committee)
Lijun	Ouyang		MD	National Clinical Research Center for Mental Disorders and Department of Psychiatry, The Second Xiangya Hospital of Central South University	Changsha, China		
				Hunan Key Laboratory of Psychiatry and Mental Health, The Second Xiangya Hospital, Central South University	Changsha, China		
				National Clinical Research Center for Geriatric Disorders, Xiangya Hospital, Central South University	Changsha, China		
Paul E	Rasser		MSc	Priority Centre for Brain and Mental Health Research, The University of Newcastle	Newcastle, NSW, Australia		
				Priority Research Centre for Stroke and Brain Injury, The University of Newcastle	Newcastle, NSW, Australia		
Franz	Resch		MD	Clinic for Child and Adolescent Psychiatry, University Hospital of Heidelberg	Heidelberg, Germany		
Jason	Schiffman		PhD	Department of Psychological Science, University of California Irvine	Irvine, CA, USA		
				Department of Psychology, University of Maryland	College Park, MD, USA		
Mikkel E	Sørensen		MSc	Centre for Neuropsychiatric Schizophrenia Research (CNSR) & Centre for Clinical Intervention and Neuropsychiatric Schizophrenia Research (CINS), Mental Health Centre Glostrup	Glostrup, Denmark		
Jinsong	Tang		MD, PhD	Department of Psychiatry, Sir Run Run Shaw Hospital, School of Medicine, Zhejiang University	Hangzhou, China		
				Key Laboratory of Medical Neurobiology of Zhejiang Province, School of Medicine, Zhejiang University	Hangzhou, China		
Dennis	Velakoulis		MD	Melbourne Neuropsychiatry Centre, Department of Psychiatry, University of Melbourne & Melbourne Health	Melbourne, VIC, Australia		

Supplemental Online Content: Nonauthor Collaborators
*First name, last name, and suffix (if applicable) are required and will appear in PubMed.

***First Name and Middle Initial(s)**	***Last Name**	***Suffix (eg, Jr, III)**	Academic Degrees	Institution	Location (city, state/province, country)	Role or Contribution, eg, chair, principal investigator	Group (if more than 1 Group listed in the byline) and/or Subgroup (eg, Steering Committee)
				Neuropsychiatry, The Royal Melbourne Hospital	Melbourne, VIC, Australia		
Sophia	Vinogradov		MD	Department of Psychiatry & Behavioral Sciences, University of Minnesota	Minneapolis, MN, USA		
Hidenori	Yamasue		MD, PhD	Department of Psychiatry, Hamamatsu University School of Medicine	Hamamatsu, Japan		
Liu	Yuan		MD	National Clinical Research Center for Mental Disorders and Department of Psychiatry, The Second Xiangya Hospital of Central South University	Changsha, China		
				Hunan Key Laboratory of Psychiatry and Mental Health, The Second Xiangya Hospital, Central South University	Changsha, China		
				National Clinical Research Center for Geriatric Disorders, Xiangya Hospital, Central South University	Changsha, China		
Alison R	Yung		PhD	Centre for Youth Mental Health, University of Melbourne	Melbourne, VIC, Australia		
				Orygen	Melbourne, VIC, Australia		
