# Supplementary material for: Normative Modeling of Brain Morphometry in Clinical High Risk for Psychosis
Source: JAMA Psychiatry. 2023 Oct 11;81(1):77–88. doi: 10.1001/jamapsychiatry.2023.3850 (PMC10568447; doi:10.1001/jamapsychiatry.2023.3850)
Supplement: Supplement 3. — Data Sharing Statement [file jamapsychiatry-e233850-s003.pdf]

Data Sharing Statement

Haas. Normative Modeling of Brain Morphometry in Clinical High Risk for Psychosis. *JAMA Psychiatry*. Published October 11, 2023. doi:10.1001/jamapsychiatry.2023.3850

Data
Data available: No

Additional Information
Explanation for why data not available: The data used within this paper is based on a pooled analysis using a limited set of anonymized variables that were made available by each participating site. Data requests are considered from members of the ENIGMA Clinical High Risk for Psychosis Working Group (https://enigma.ini.usc.edu/ongoing/enigma-clinical-high-risk/), and any researcher with the ability to contribute neuroimaging and/or clinical data of CHR participants are encouraged to join the working group. Please contact the Co-Chairs Maria Jalbrzikowski (Maria.Jalbrzikowski@childrens.harvard.edu) and Dennis Hernaus (dennis.hernaus@maastrichtuniversity.nl) of the ENIGMA Clinical High Risk for Psychosis Working Group if you are interested in joining. The normative model used to compute individual-level brain morphometric deviations is openly accessible here: https://centilebrain.org/#/model. Code for additional analyses described within this manuscript are available here: https://github.com/shalailahaas/ENIGMA-CHR-P-Normative-Modeling.
